# Secondary Metabolites with Antimycobacterial Activities from One Actinobacteria: *Herbidospora yilanensis*

**DOI:** 10.3390/molecules26206236

**Published:** 2021-10-15

**Authors:** Ming-Jen Cheng, Ming-Der Wu, Jih-Jung Chen, Yung-Shun Su, Yueh-Hsiung Kuo

**Affiliations:** 1 Bioresource Collection and Research Center, Food Industry Research and Development Institute, Hsinchu 300, Taiwan; 2 Department of Pharmacy, School of Pharmaceutical Sciences, National Yang Ming Chiao Tung University, Taipei 112, Taiwan; 3 Department of Medical Research, China Medical University Hospital, Taichung 404, Taiwan; yhkuo800@gmail.com; 4 Graduate Institute of Medicine, College of Medicine, Kaohsiung Medical University, Kaohsiung 807, Taiwan; mariussu@gmail.com; 5 Department of Dermatology, Kaohsiung Medical University Chung-Ho Memorial Hospital, Kaohsiung 807, Taiwan; 6 Department of Chemistry, National Taiwan University, Taipei 106, Taiwan; 7 Department of Biotechnology, Asia University, Taichung 413, Taiwan; 8 Department of Chinese Pharmaceutical Sciences and Chinese Medicine Resources, College of Pharmacy, China Medical University, Taichung 404, Taiwan

**Keywords:** *Herbidospora* *yilanensis*, Streptosporangiaceae, diterpenoid, antimycobacterial activities

## Abstract

The cultivation of one actinobacteria strain, Herbidospora yilanensis, was isolated from sediment samples collected from Yilan County City in Taiwan, resulting in the isolation of five previously undescribed compounds: herbidosporayilanensins A–E (**1**–**5**), and four compounds isolated from nature for the first time: herbidosporayilanensins F–I (**6**–**9**). Their structures were elucidated by spectroscopic analyses, including 1D- and 2D-NMR experiments with those of known analogues, and on the basis of HR-EI-MS mass spectrometry, their antimycobacterial activities were also evaluated.

## 1. Introduction

Actinobacteria have the ability to produce a variety of physiologically active products, so they play a very important role in the food industry, pharmaceutical industry, and environmental protection. Our team has also separated and collected actinobacteria resources from all over Taiwan and various environments over the years, except for common chains. In addition to molds, there are many rare genera. Based on the concept of “new species and new compounds”, it is hoped that special compounds can be found from these new species. In recent years, studies have also found that these new species of actinobacteria can produce many active secondary metabolites. In order to further explore the efficacy of different strains of actinobacteria and expand the application range of actinobacteria, this study focused on a strain from central Taiwan, and the new strain *H*. *yilanensis* isolated from the sediments of northern rivers and lakes is very novel and worthy of in-depth research and discussion.

**Figure 1:**
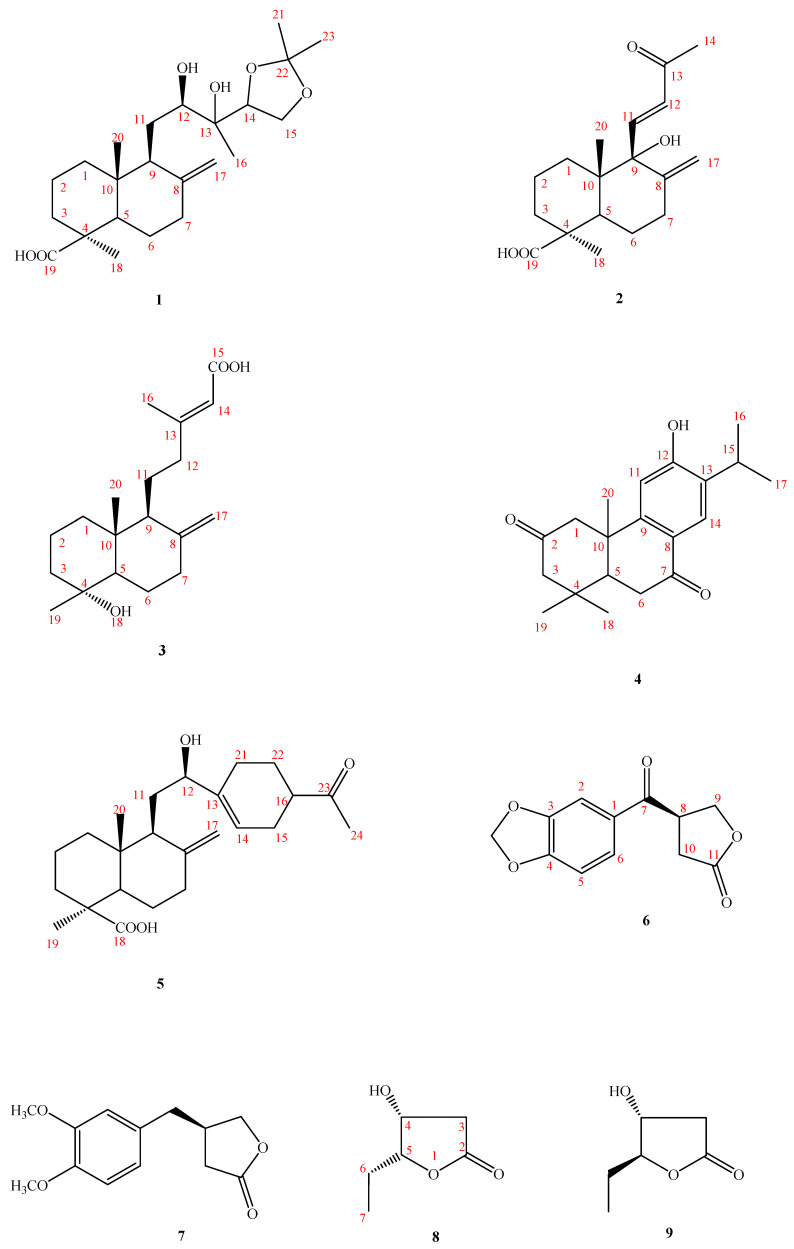
Compounds **1–9**, isolated from *Herbidospora yilanensis*.

Actinobacteria are widely distributed in nature, and they are very useful in the pharmaceutical industry due to their seemingly unlimited capacity to produce secondary metabolites with diverse chemical structures and biological activities [1,2,3]. They are Gram positive, free-living saprophytic bacteria that are widely distributed in soil, water, and colonizing plants. Actinobacteria inhabitants have been identified as one of the major groups of soil populations [2,3,4,5], which may vary with the soil type. With the plan to investigate the diversity of cultivable actinobacteria associated with soil from Taiwan, we isolated a strain named 0351M-12^T^, which was isolated from a sediment sample collected from the Yilan County of Taiwan with a unique morphology [6]. This strain was determined to be *Herbidospora yilanensis* (Family: Streptosporangiaceae) based on its phenotypic and genotypic data [6]. Strains of the genus *Herbidospora* have grass-like vegetative hyphae, which develop from vegetative hyphae into spore chains, without obvious aerial hyphae. Although it was officially published in 1993, only seven species of bacteria have been published thus far, so there are few related development studies. Mainly isolated from soil, river bottom mud, rotted leaves, petals, and tree trunks, most strains of the genus *Herbidospora* growth requires vitamin B. *H*. *yilanensis* is a new strain isolated from the sediments of rivers or lakes in northern Taiwan. It is very novel and worthy of in-depth research. The current study was carried out on the bioactive metabolites of the *n*-BuOH-soluble fraction of the 95% ethanolic extract of rice fermented with the actinobacteria *H*. *yilanensis*. This led to the isolation of five previously undescribed compounds: herbidosporayilanensins A–E (**1**–**5**), and four compounds: herbidosporayilanensins F–I (**6**–**9**), isolated for the first time from nature (Figure 1). The structures of these isolates were established by means of spectral experiments. We hereby report the isolation, structure determination/identification, and the antimycobacterial activity of some isolates (Supplemental Figures S1–S63).

## 2. Results and Discussion

### Structure Elucidation of Compounds

Compound **1** was isolated as an optically active oil with $[\alpha {]}_{D}^{25}$: + 10.6 (*c* 0.071, CHCl_3_). The HR-EI-MS gave an [M]^+^ ion peak at *m*/*z* 410.2667 (calcd 410.2666), consistent with a molecular formula of C_2__3_H_3__8_O_6_. The IR absorption bands suggested the presence of a COOH and OH (2500–3400 cm^−1^), a terminal double bond (1664, 858 cm^−1^), and a CO (1698 cm^−1^) group. The ^13^C-NMR (DEPT) spectrum of 1 (Table 1) exhibited 23 signals: four Me, eight CH_2_ (one being oxygenated and one exocyclic double bond), and four CH groups (two being oxygenated), as well as five quaternary C-atoms, including a C=O function (δ_C_ 182.6).

Five indices of hydrogen deficiency (IHD) were determined from the molecular formula. After subtracting one double bonds and carboxyl groups, the remaining unsaturation degree is 3, and then the three typical methyl groups at δH 1.25 (s), 1.22 (s), and 0.59 (s) in 1H-NMR, it was deduced that compound 1 is a diterpenoid containing two six-membered labdane-type structures, and the basic structure of labdane is a C20 compound, so it was deduced that it contains a three-carbon derivative functional groups, which contain the two methyl signals δH 1.39 (s) and 1.35 (s).

**Figure 2:**
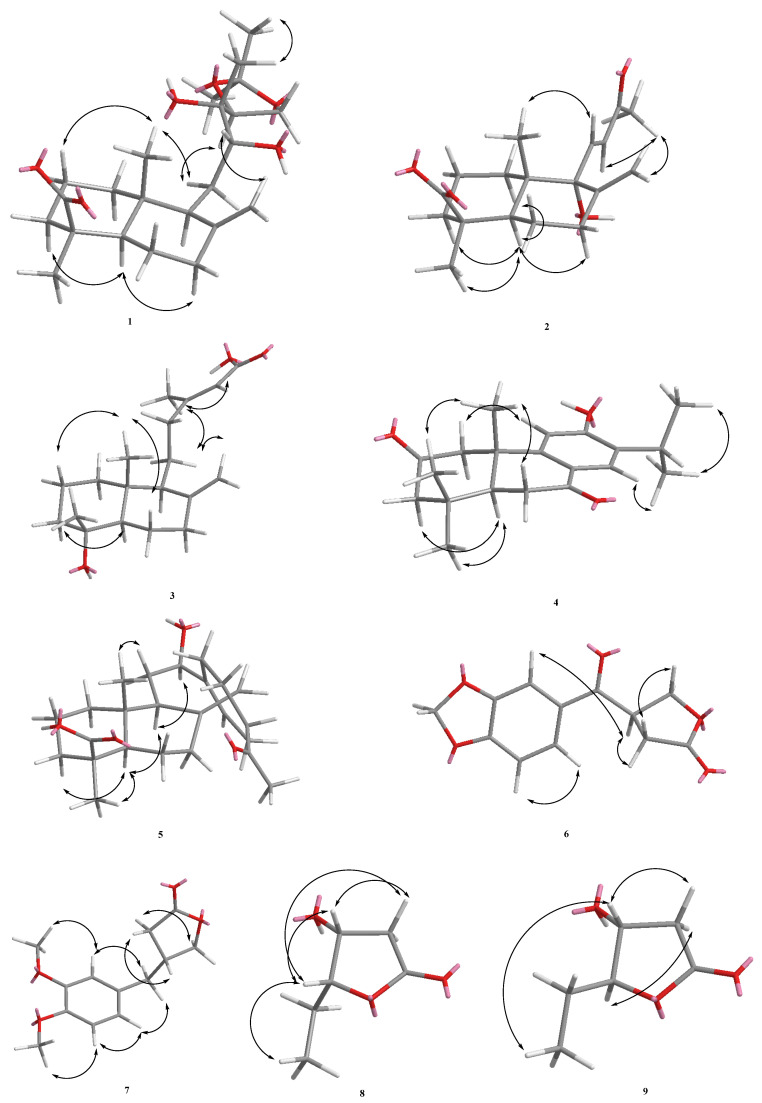
Major NOESY (↔) contacts of **1**–**9**.

From the ^13^C-NMR spectrum at δ_C_ 108.4, a signal was found and confirmed by DEPT as a quaternary carbon. Furthermore, the HMBC spectrum showed that H-21/H-23 were correlated with C-22, along with no COSY correlations between H-21 and H-23, and established that two methyl groups are located at C-22 (Figure 1). The quaternary carbon (δ_C_ 108.4) had such a low field that it must be a conjugated double bond, but this quaternary carbon did not have other conjugated double bonds and was connected to two methyl groups. Therefore, it was deduced that this quaternary carbon is an acetonide functional group with two oxygens. In combination with the HMBC correlations of H-15, H-21, and H-23 to C-22, this led to the establishment of acetonide being located on C-14 and C-15 instead of the C-12/C-13 or C-13/C-14 position. From the HSQC spectrum, it can be seen that the proton signal corresponding to δ_C_ 74.4 is δ_H_ 3.56 (1H, d, *J =* 10.5 Hz), and it has an HMBC correlation with C-9, C-11, and C-13. It was inferred that δ_C_ 74.4 is C-12, and that C-12 is connected to a hydroxyl group (-OH). In addition, δ_C_ 73.9 has an HMBC correlation with H-12, H-14, and H-16 and from the IR, it was found that there was an OH signal at 3418 cm^−1^, so it was inferred that δ_C_ 73.9 is C-13, which is connected to a hydroxyl group (-OH), and δ_H_ 1.25 (s) is correlated to C-12, C-13, and C-14, so this methyl group was deduced as being located on C-13. From the NOESY results, H-11 is correlated to H-20, it can be confirmed that C-20 is in the axial. The presence of NOESY correlations between H-11 and H-20, between H-18 and H-3/5, and the absence between H-18 and H-20 indicated that Me-18 was α-orientated at C-4 in equatorial (Figure 2).

According to the labdane-type analogues mentioned in the literature [7], if the absolute configuration of (12*R*) is determined, the ^1^H-NMR chemical shift of one of the protons on C-17 will be close to about δ_H_ 4.50 (1H, br s, H-17a). In compound **1**, one of the hydrogens at the C-17 position was δ_H_ 4.53 (br s) and is similar to the reference analog, (12*R*)-Hydroxylabda-8(17),13(*Z*)-dien-12,19-dioic acid (δ_H_ 4.50 (1H, br s, H-17a); in an opposite reference compound (12*S*)-hydroxylabda-8(17),13(*Z*)-dien-12,19-dioic acid, its H-17a is about δ_H_ 4.71 (1H, br s, H-17a)) [7], and it was deduced that the hydroxyl group at C-12 is *R*-form. Thus, from the above data, the structure of compound **1** was established as 12*R*,13-diol-13,14-acetonide-8(17)-ene-19-oic acid, namely, herbidosporayilanensin A.

Compound **2** was obtained as colorless oil. The molecular formula was determined as C_18_H_26_O_4_ on the basis of HR-EI-MS and NMR data. The NMR data of **2** (Table 1) were similar to those of 15,16-bisnor-13-oxo-8(17),11(*E*)-labdadien-19-oic acid [8], except that **2** contained one more OH group at C-9 (δ_C_ 70.9) as inferred from HMBC correlations between H-11, 12, 17, 20 and C-9. The configuration of **2** was determined based on the same NOSEY correlations as in 15,16-bisnor-13-oxo-8(17),11(E)-labdadien-19-oic acid. From the above data, the structure of compound **2** was, thus, determined as 15,16-bisnor-9-hydroxyl-13-oxo79 8(17),11(E),-labdadien-19-oic acid and it was named herbidosporayilanensin B.

**Figure 3:**
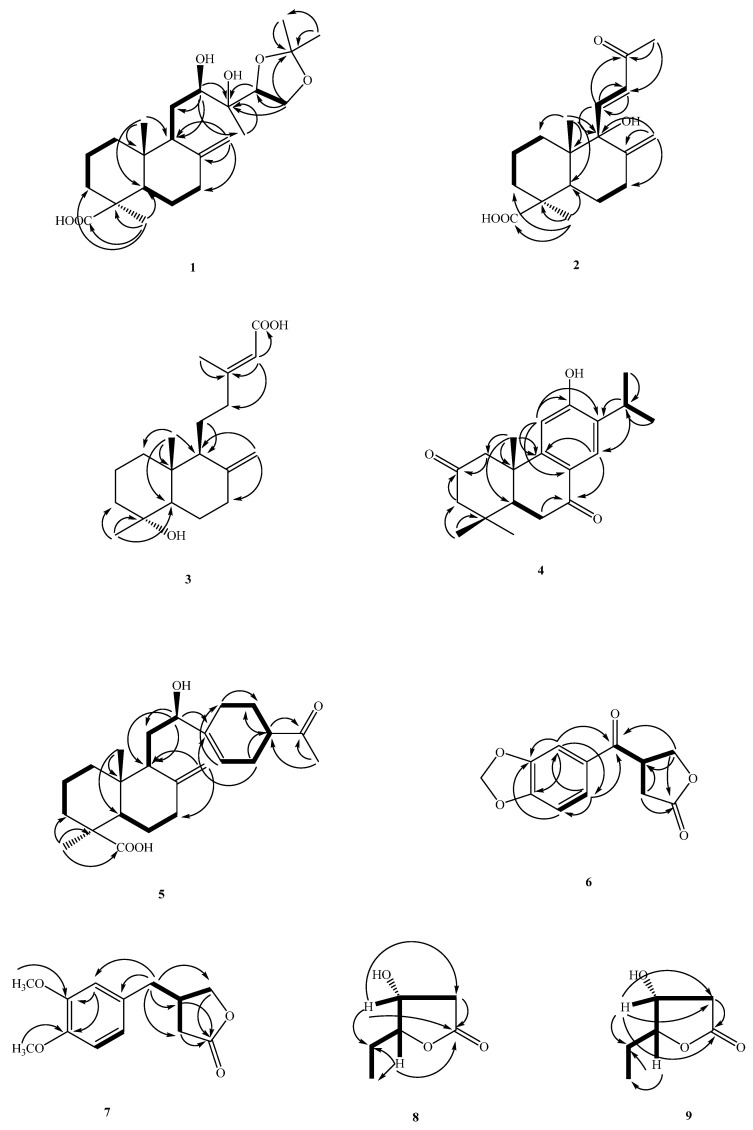
Key COSY (▬) and HMBC (→) correlations of **1**–**9**.

Compound **3** was obtained as a colorless oil with a specific rotation similar to that of labda-8(17),13(*E*)-diene-15-oic acid. The molecular formula was determined to be C_19_H_30_O_3_ from the HR-EI-MS mass spectrum (*m*/*z* 306.2193 ([M]^+^; calc. 306.2195)). The ^1^H-, and ^13^C-NMR spectrum of **3** was similar to labda-8(17),13(*E*)-diene-15-oic acid [9], and both had the same labdane moiety [9]. The major difference was the presence of an OH group attached to C-4 [δ_C_ 72.5)] in **3**, instead of a Me group (*δ*_H_ 0.86 (Me-19); *δ*_C_ 72.5 (C-19)) at C-4 (δ_C_ 33.6) in labda-8(17),13(*E*)-diene-15-oic acid. The ^1^H-NMR, ^13^C-NMR (Table 2), COSY, NOESY (Figure 3), HSQC, and HMBC (Figure 3) data confirmed the structure as 4α-Hydroxylabda-8(17),13(*E*) -triene-15-oic acid, named herbidosporayilanensin C.

Compound **4** was isolated as colorless oil with $[\alpha {]}_{D}^{25}$: +60.6 (*c* 0.011, MeOH). The HR-EI-MS data determined the molecular formula to be C_20_H_26_O_3_ (*m*/*z* 314.1884 ([M]^+^; calc. 314.1882)). The UV absorption of **4** at 280 nm suggested the presence of a benzenoid nucleus. The IR spectrum showed absorption bands for a hydroxyl group at 3400 cm^−1^ and an aromatic ring at 1597 and 1505 cm^−1^, respectively.

The ^1^H-NMR and ^13^C-NMR spectra (Table 2) of **4** were similar to those of sugiol [10], except that a keto group [δ_C_ 209.9 (C-2)] of **4** replaced a methylene group [δ_C_ 19.4 (C-2)] at C-2 of sugiol. The ^1^H- and ^13^C-NMR (Table 2), HMBC (Figure 2), COSY (Figure 2), and NOESY (Figure 3) were compatible with the structure of **4** as 6-hydroxy-7-isopropyl-1,1,4a-trimethyl-1,2,4,4a,10,10a-hexahydrophenanthrene-3,9-dione, named herbidosporayilanensin D.

Compound **5**, an oil, has a molecular formula of C_24_H_36_O_4_ as determined by HR-EI-MS at *m*/*z* 388.2618 [M]^+^ (calcd. for C_24_H_36_O_4_, 388.2618), corresponding to seven degrees of unsaturation. The IR spectrum of **5** showed absorption bands at 3410; 1696 cm^−1^ ascribable to hydroxyl, and C=O groups. The ^1^H and ^13^C NMR spectral data of **5** showed signals for a terminal vinyl group, one acetyl, a hydroxyl-substituted methine, one carboxylic acid, and two singlet methyl groups. A careful comparison of the ^13^C NMR data of **5** (Table 2) with those of 12,13-dihydroxylabda-8(17),14-dien-19-oic acid [11] indicated that the two compounds shared the same skeleton and had minor differences in C-12 side chain.

A major difference was that a 4-acetylcyclohex-1-en-1-yl group at C-12 [δ_H_ 2.07/2.12 (each 1H, m, H-21), 1.57/2.00 (each 1H, m, H-22), 2.56 (1H, m, H-16), 2.19 (1H, m, H-15), 5.65 (1H, br. s, H-14)] in **5** replaced a but-3-en-2-ol moiety (δ_H_ 1.34 (3H, s, Me-16), 5.19 (1H, d, *J =* 10.8, H-15), 5.33 (1H, d, *J =* 18.0, H-15), 5.91(1H, dd, J=18.0, 10.8Hz, H-14)) in the C-12 position in 12,13-dihydroxylabda-8(17),14-dien-19-oic acid. A 4-acetylcyclohex-1-en-1-yl group was located at C-12 further confirmed by the HMBC correlations from H–12 ((δ_H_ 3.99) to C-14 ((δ_C_ 119.4), H-14 ((δ_H_ 5.65) to C-12 (δ_C_ 73.9). The ^1^H- and ^13^C-NMR (Table 2), HMBC (Figure 2), COSY (Figure 2), and NOESY (Figure 2) were compatible with the structure of **5** as 5-(2-(4-acetylcyclohex-1-en-1-yl)-2-hydroxyethyl)-1,4a-dimethyl-6-methylenedecahydronaphthalene-1-carboxylic acid, named herbidosporayilanensin E.

Compound **6** was isolated as an amorphous solid with a positive optical rotation $[\alpha {]}_{D}^{25}$ = +6.0 (*c* 0.02, CHCl_3_) and UV λ_max_ at 230, 276, 312 nm revealing the presence of the conjugated system. The EI-MS of **1** showed a molecular ion peak at *m*/*z* 234 [M]^+^, and the molecular formula C_12_H_10_O_5_ of **6** was resolved using HR-EI-MS. The IR (KBr) spectrum of **6** showed absorption bands at 1770; 1668 cm^−1^ ascribable to γ-lactone, and conjugated C=O groups. The ^1^H-NMR spectrum of **6** displayed signals for a ABX-pattern splits benzene ring (δ_H_ 7.48 (1H, dd, *J =* 8.0, 2.0 Hz, H-6), 6.88 (1H, d, *J =* 8.0 Hz, H-5) and 7.40 (1H, d, *J =* 2.0 Hz, H-2)), two methylene groups (δ_H_ 2.75 (1H, dd, *J =* 17.6, 9.2 Hz, H-10), 3.00 (1H, dd, *J =* 17.6, 9.2 Hz, H-10), 4.43 (1H, dd, *J =* 9.2, 6.8 Hz, H-9), 4.57 (1H, t, *J =* 9.2 Hz, H-9), one methine (δ_H_ 4.27 (1H, m, H-8)), and one methylenedioxy at δ_H_ 6.07 (2H, s, OCH_2_O). From the ^1^^3^C-NMR, there was a benzene rings at δ_C_ 108.1 (C-2), 148.8 (C-3), 152.8 (C-4), 108.3 (C-5), 124.9 (C-6), 129.8 (C-1), with two carbonyl groups, one of which was δ_C_ 194.1 (C-7), which is a conjugated carbonyl group. UV absorption at 231 nm showed that it should be adjacent to the benzene ring. The carbon signal at δ_C_ 175.29 (C-11) is a characteristic signal of the γ-lactone, which forms a five-ring lactone with the remaining signals at δ_C_ 69.19 (C-9), 31.06 (C-10), and 41.97 (C-8). This is the framework of C6-C5 type. Compound **6** showed dextrorotatory optical activity with $[\alpha {]}_{D}^{25}$ = +6.0 (*c* 0.02, CHCl_3_). By comparing the reference to the *R*-configuration of (*R*)-(+)-4-(3-Methoxyphenyl)methylbutyrolactone [12] ($[\alpha {]}_{D}^{20}$ = +5.5, CHCl_3_), the absolute configuration at C-8 should be tentatively proposed as *R*. The ^1^H- and ^13^C-NMR (Table 2), COSY (Figure 2), NOESY (Figure 3), HSQC and HMBC (Figure 2) experiments confirmed the structure as (*R*)-4-(benzo[*d*][1,3]dioxole-5-carbonyl)dihydrofuran-2(3*H*)-one, and was designated herbidosporayilanensin F. Compound **6** was first isolated from a natural source, though it has never been synthesized [12].

Compound **7** was isolated as an amorphous solid with positive specific rotation, $[\alpha {]}_{D}^{25}$ = +11.2. The molecular formula of **7** (C_13_H_16_O_4_) was established by the [M]^+^ ion peak at *m*/*z* 236.1044 in the HREIMS. The UV spectrum of **8** showed maximal absorptions between 229–280 nm, indicating the presence of a benzenoid moiety [13]. The IR spectrum exhibited absorption characteristic at 1594, 1463, and 1779 cm^−1^ attributed to aromatic ring and γ-lactone functionalities, respectively.

The ^1^H-NMR and ^13^C-NMR spectra (Table 1 and Table 2) of **7** were similar to those of **6**, except that two methoxyl groups [δ_H_ 3.86 (3H, *s*, OMe–3 and 4)] and the CH_2_ [δ_H_ 2.70 (2H, m, H–7)] of **7** replaced a OCH_2_O group [δ_H_ 6.07 (2H, *s*, OCH_2_O–3 and 4)] at C-3 and 4 and a C=O at C-7 [δ_C_ 194.1 (C-7)] of **6**. The dextrorotatory optical activity {$[\alpha {]}_{D}^{25}$ = +11.2 (*c* 0.18, CHCl_3_} once again indicated the stereochemistry of C-8 as 8*R* [12]. The ^1^H- and ^13^C-NMR (Table 1 and Table 2), COSY (Figure 2), NOESY (Figure 3), HSQC, and HMBC (Figure 2) were compatible with the structure of **7** as (*R*)-4-(3,4-dimethoxybenzyl)dihydrofuran-2(3*H*)-one, named herbidosporayilanensin G. Accordingly, 7 was found in nature for the first time [13].

Compound **8**, a colorless oil, $[\alpha {]}_{D}^{25}$= –33.8, gave the [M]^+^ ion peak at *m*/*z* 131 in EI-MS. The HR-EI-MS data determined the molecular formula to be C_1__0_H_10_O_3_ (*m*/*z* 131.0721 ([M]^+^; calc. 131.0712)). The IR spectrum showed absorption bands for a hydroxyl group at 3422 cm^−1^ and a butyrolactone group at 1769 cm^−1^, along with a resonance signal in the ^13^C-NMR spectrum at δ_C_ 195.0. Moreover, the ^13^C-NMR spectrum, in combination with DEPT and HSQC experiments, showed signals for two oxymethines at δ_C_ 85.9 (C-5), 68.8 (C-4), one methylene at δ_C_ 39.5 (C-3), and one ethyl at δ_C_ 9.9 (C-7 and 21.5 (C-6), respectively. All the above data indicated that **8** was a dihydrofuran-2-one derivative (= butyrolactone)). The ^1^H NMR data of **8** (Table 2) displayed the occurrence of a butyrolactone derivative, with a 5-ethyl-4-hydroxydihydrofuran-2(3*H*)-one moiety [δ_H_ 4.49 (1H, ddd, *J =* 5.2, 4.0, 1.2 Hz, H-4), 4.28 (1H, ddd, *J*= 7.6, 6.8, 4.0 Hz, H-5), 2.79 (1H, dd, *J =* 17.6, 5.2 Hz, H-3α), 2.53 (1H, dd, *J =* 17.6, 1.2 Hz, H-3β), 1.88 (1H, m, H-6), 1.75 (1H, m, H-6), 1.05 (1H, t, *J =* 7.2 Hz, H-7)], and the HMBC spectrum (Figure 2) supported the planar structure as illustrated in Figure 1. The above observations were further confirmed by the NOE correlations between CH_2_-3/H-5, H-4/H-5, and H-5/CH_3_-7. With aid of HMBC and NOESY, the relative configuration could be inferred, and it was further seen that H-4 and H-5 are in a *cis* relationship. The laevorotatory optical activity of **8** indicated the C-4 hydroxyl group in *R*-configuration [14,15], and the chemical shifts of H-4 (ca. *δ*_H_ 4.55) and 7-Me (ca. *δ*_H_ 1.34) of **8** were also similar to those of related analogues. The NOESY correlations were observed for H-4/H-5, indicating that they were on the same side of the molecular plane, assumed to have an *R*-orientation. Therefore, the absolute configuration of C-4 and C-5 of **8** was deduced to be the (4*R*,5*R*) configuration [14,15]. This compound has been synthesized in the literature [16], but it was isolated for the first time as a natural product. Based on the spectral evidence, the structure of **8** was elucidated as (4*R*,5*R*)-5-ethyl-4-hydroxydihydrofuran-2(3*H*)-one, named herbidosporayilanensin H, which was further confirmed by ^1^H- and ^13^C-NMR chemical shifts (Table 1 and Table 2), and the HMBC, ^1^H,^1^H-COSY, and NOESY correlations are shown in Figure 2.

Compound **9** was also isolated as colorless oil. The EI-MS afforded the positive ion at *m*/*z* 278 [M]^+^, implying a molecular formula of C_17_H_26_O_3_. The IR spectrum showed absorption bands for a hydroxyl group at 3424 cm^−1^ and an α,β-unsaturated-γ-lactone at 1731 and 1650 cm^−1^. From the spectral evidence, compound **9** was similar with **8**, and also had the same β-hydroxy-γ-methyl-γ-lactone skeleton. Furthermore, compound **9** showed dextrorotatory optical activity with {[α]_D_: +4.7° (*c* = 0.022, CHCl_3_)}, and the absolute configuration of C-4 and 5 was proposed as (4*R*,5*S*) after comparing the similar analog [litsealiicolide A $[\alpha {]}_{D}^{25}$: +6.7° (*c* = 0.21, CHCl_3_)] [14,15]. No NOESY contacts were observed for H-4/H-5, once it has been indicating that they were on the opposite side of the molecular plane, assumed to have a (4*R*,5*S*)-orientation instead of a (4*R*,5*R*) in **8**. Thus, the structure of **8** was established, as shown in Figure 1 and was named herbidosporayilanensin I.

The biological activities of the isolates present in sufficient amounts (**1**–**7**) in compounds from fermented broth were filtered to separate the mycelium and culture broth. The culture broth was repeatedly extracted three times with EtOAc. The EtOAc-soluble fraction of the fermented broth of the actinobacteria *H*. *yilanensis* was tested in vitro against *M*. *tuberculosis* strain H_37_Rv. The antimycobacterial activity data are shown in Table 3. The clinically used antimycobacterial agent, ethambutol, was used as a positive control. The results of the antimycobacterial activities indicated that herbidosporayilanensin A (**1**), herbidosporayilanensin A (**2**), and herbidosporayilanensin F (**6**) exhibited more potent antimycobacterial activities against *M*. *tuberculosis* strain H_37_Rv in vitro, showing MIC values of 16.6, 19.2, and 18.2 μM, respectively, than did the clinical drug, ethambutol (MIC 6.25 μg/mL). The results showed moderate antimycobacterial activity, indicating that **3** and **4** had MIC values of 40.8 and 50.6 μg/mL, respectively. Compounds **5** and **7** showed no antimycobacterial activities. When comparing the two benzenoids (**6**–**7**), the presence of ketone groups on the 7 position of the side chain in **6** was seen to play an important role in antimycobacterial activity.

## 3. Materials and Methods

### 3.1. General Experimental Procedures

For TLC, we used silica gel 60 F_254_ precoated plates (Merck); for column chromatography (CC), silica gel *60* (70–230 or 230–400 mesh, Merck) and Spherical C18 100A Reversed Phase Silica Gel (RP-18) (particle size: 20–40 μm) (Silicycle). For HPLC, we used spherical C18 column (250 × 10 mm, 5 μm) (Waters) and LDC-Analytical-III apparatus. For the UV spectra, we used a Jasco UV-240 spectrophotometer, λ_max_ (log ε) in nm. For the optical rotation, we used Jasco DIP-370 polarimeter, in CHCl_3_. For the IR spectra, we used a Perkin-Elmer-2000 FT-IR spectrophotometer; ν in cm^−1^. For the ^1^H-, ^13^C- and 2D-NMR spectra, we used Varian-Mercury-500 and Varian-Unity-Plus-400 spectrometers; *δ* in ppm rel. to Me_4_Si, *J* in Hz. For ESI and HRESIMS, we used a Bruker APEX-II mass spectrometer, in *m*/*z*.

### 3.2. Microorganism, Cultivation, and Preparation of the Actinobacteria Strain

The actinobacteria, *Herbidospora yilanensis* (0351M-12^T^), was isolated from sediment collected from the northern area of Taiwan using HVY agar and was then incubated at 45 °C for 7 days. This actinobacteria was identified by Mrs. Min Tseng, and the specimens (0351M-12^T^) were deposited at the Bioresource Collection and Research Center (BCRC) of the Food Industry Research and Development Institute (FIRDI). The strain was maintained on oatmeal agar, and the spores or mycelia suspension were harvested with 20% (*v*/*v*) glycerol and stored at −20 °C. A mature slant culture of strain 0351M-12^T^ was inoculated into a 500 mL flask containing 100 mL of the seed medium consisting of 0.4% glucose, 0.4% yeast extract, and 1% malt extract (pH 7.3). After growing at 30 °C for 4 d on a rotary shaker (200 rpm), the aliquots (2 mL) of the seed culture were transferred into a 500 mL flask containing 200 mL of production medium (Humic acid 1.0 g, Na_2_HPO_4_ 0.5 g, KCl 1.7 g, MgSO_4_ 7H_2_O 0.05 g, FeSO_4_ 7H_2_O 0.01 g, CaCO_3_ 0.02 g, yeast extract 1.0 g, Agar 20.0 g, dist. water 1.0 L, pH 7.4). After 18 days cultivation at 30 °C temperature on a rotary shaker (200 rpm), the culture filtrates were obtained by filtering through filter paper.

### 3.3. Isolation and Characterization of Secondary Metabolites

Fermented broth (3 l) was filtered to separate the mycelium and culture broth. The culture broth was repeatedly extracted three times with EtOAc. The EtOAc layers were combined and dried to give fraction-soluble EtOAc (25.2 g).

The EtOAc fraction (25.2 g) was applied to silica gel column (230–400 mesh, 800 g), eluting with a gradient of *n*-hexane/acetone to give 10 fractions (1–10). Fraction 2 (1237 mg) was applied to a silica gel (230–400 mesh, 30 g), eluting with a gradient of *n*-hexane/acetone to give eight fractions (2-1–2-8). Fraction 2-1 (179 mg) was chromatographed on a Sephadex LH-20 column (MeOH) to give five fractions (2-2-1–2-2-5). Fr. 2-2-3 (19 mg) was applied to an RP-C18 column (10 g), eluting with acetonitrile/H_2_O (2.5:1) to obtain **1** (1.9 mg) and **2** (1.8 mg). Fr. 2-2-4 (89.4 mg) was chromatographed on a silica gel column (230-400 mesh, 1 g) eluting with CHCl_3_/MeOH (20:1) to give seven fractions (2-2-4-1~2-2-4-7). Fr. 2-2-4-3 was applied to an RP-C18 column, eluting with acetonitrile/H_2_O (3:1) to obtain **7** (2.8 mg) and **3** (1.1 mg). Fraction 5 (13.2 mg) was further purified by preparative RP-18 TLC (MeOH-H_2_O, 5:1) to afford **6** (3.8 mg) and **9** (0.53 mg). Fraction 7 (3.20 g) was applied to a silica gel column (230–400 mesh, 90 g), eluting with CH_2_Cl_2_ to obtain seven fractions (7-1–7-7). Fraction 7-3 (85 mg) was chromatographed on an RP-C18 column (1 g), eluting with MeOH/H_2_O (2.5:1) to afford **5** (3.1 mg). Fraction 8 (2.25 g) was chromatographed on a silica gel column (230–400 mesh, 60 g), eluting with a gradient of *n*-hexane/acetone, to give 10 fractions (8-1–8-10). Fr. 8-7 (188.6 mg) was chromatographed on an RP-18 column (2 g), eluting with (acetone/H_2_O, 2:1) to afford **4** (5.1 mg) and **9** (0.41 mg).

Herbidosporayilanensin A (**1**): oil; $[\alpha {]}_{D}^{25}$= +10.6 (*c* 0.01, CHCl_3_); IR (Neat): 3418(-OH), 2500–3400 (COOH), 1698 (COOH), 1644, 858 (C=CH2) cm^−1^; ^1^H NMR (500 MHz, CDCl_3_): see Table 1; ^13^C NMR (125 MHz, CDCl_3_): see Table 2); EIMS (70 eV) *m*/*z* (%):410 ([M]+, 100), 392 (5), 370 (44), 321 (34); HREIMS *m*/*z* 410.2667 [M]^+^ (calcd. for C_22_H_38_O_6_, 410.2666).

Herbidosporayilanensin B (**2**): oil; $[\alpha {]}_{D}^{25}$ = +10.2 (*c* 0.01, CHCl_3_); UV (MeOH): 224 (4.11) nm; IR (Neat): 3462 (OH), 2500–3400 (COOH), 1693 (COOH), 1664, 905 (C=CH_2_) cm^−1^; ^1^H NMR (500 MHz, CDCl_3_): see Table 1; ^13^C NMR (125 MHz, CDCl_3_): see Table 2); EIMS (70 eV) *m*/*z* (%): 306 ([M]^+^, 100), 275 (4); HREIMS *m*/*z* 306.1835 [M]^+^ (calcd. for C_18_H_26_O_4_, 306.1831).

Herbidosporayilanensin C (**3**): oil; $[\alpha {]}_{D}^{30}$= +16.4 (*c* 0.012, CHCl_3_); UV (MeOH): 220 (4.00) nm; IR (Neat): 3444 (OH), 2500–3400 (COOH), 1682 (COOH), 1645, 1660, 894 (C=CH_2_) cm^−1^; ^1^H NMR (500 MHz, CDCl_3_): see Table 1; ^13^C NMR (125 MHz, CDCl_3_): see Table 2); EIMS (70 eV) *m*/*z* (%):332 ([M]^+^, 64), 314 (74), 145 (23), 105 (30); HREIMS *m*/*z* 332.1960 [M]^+^ (calcd. for C_20_H_28_O_4_, 332.1982).

Herbidosporayilanensin D (**4**): oil; $[\alpha {]}_{D}^{25}$ = +60 (*c* 0.01, CHCl_3_); UV (MeOH): 294 (4.31), 280 (4.23), 228 (4.70) nm; IR (Neat): 3400 (OH), 1651 (conjugated C=O), 1597, 1505 (aromatic ring) cm^−1^; ^1^H NMR (500 MHz, CDCl_3_): see Table 1; ^13^C NMR (125 MHz, CDCl_3_): see Table 2); EIMS (70 eV) *m*/*z* (%): 330 ([M]^+^, 41), 312 (9), 285 (12), 121 (98), 55 (100); HREIMS *m*/*z* 314.1884 [M]^+^ (calcd. for C_20_H_26_O_3_, 314.1882).

Herbidosporayilanensin E (**5**): oil; $[\alpha {]}_{D}^{25}$= +21.4 (*c* 0.01, CHCl_3_); IR (Neat): 2500~3400 (COOH), 3403 (OH), 1696 (COOH), 1645, 890 (C=CH_2_) cm^−1^; ^1^H NMR (500 MHz, CDCl_3_): see Table 1; ^13^C NMR (125 MHz, CDCl_3_): see Table 2); EIMS (70 eV) *m*/*z* (%):387([M - H]+, 16), 333 (61), 321 (100), 313 (38), 123 (32); HREIMS *m*/*z* 388.2615 [M]^+^ (calcd. for C_24_H_36_O_4_, 388.2614).

Herbidosporayilanensin F (**6**): amorphous solid.; $[\alpha {]}_{D}^{25}$= +6.0 (*c* 0.01, CHCl_3_); UV (MeOH): 231 (4.20), 276 (3.78), 312 (3.85) nm; IR (KBr): 1770 (OCO), 1668 (CO), 1605,1506, 1419 (aromatic ring) cm^−1^; ^1^H NMR (500 MHz, CDCl_3_): see Table 1; ^13^C NMR (125 MHz, CDCl_3_): see Table 2); EIMS (70 eV) *m*/*z* (%): 234 ([M]^+^, 19), 149 (100), 121 (13); HREIMS *m*/*z* 238.0538 [M]^+^ (calcd. for C_12_H_10_O_5_, 238.0538).

Herbidosporayilanensin G (**7**): amorphous solid.; $[\alpha {]}_{D}^{25}$= +11.2 (*c* 0.01, CHCl_3_); UV (MeOH): 230 (4.02), 280 (3.59) nm; IR (KBr): 1779 (OCO), 1594, 1517, 1463 (aromatic ring) cm^−1^; ^1^H NMR (500 MHz, CDCl_3_): see Table 1; ^13^C NMR (125 MHz, CDCl_3_): see Table 2); EIMS (70 eV) *m*/*z* (%): 234 ([M]^+^, 19), 149 (100), 121 (13); HREIMS *m*/*z* 236.1044 [M]^+^ (calcd. for C_13_H_16_O_4_, 236.1048).

Herbidosporayilanensin H (**8**): oil.; $[\alpha {]}_{D}^{25}$= +33.8 (*c* 0.01, CHCl_3_); IR (Neat): 3422 (OH), 1769 (γ-lactone ring) cm^−1^; ^1^H NMR (500 MHz, CDCl_3_): δ_H_ 1.05 (1H, t, *J =* 7.2 Hz, H-7), 1.75 (1H, m, H-6), 1.88 (1H, m, H-6), 2.53 (1H, dd, *J =* 17.6, 1.2 Hz, H-3β), 2.79 (1H, dd, *J =* 17.6, 5.2 Hz, H-3α), 4.28 (1H, ddd, *J* = 7.6, 6.8, 4.0 Hz, H-5), 4.49 (1H, ddd, *J =* 5.2, 4.0, 1.2 Hz, H-4); ^13^C NMR (125 MHz, CDCl_3_): δ_C_ 9.9 (C-7), 21.5 (C-6), 39.5 (C-3), 68.8 (C-4), 85.9 (C-5), 175.4 (C-2).; EIMS (70 eV) *m*/*z* (%): 131 ([M+H]^+^, 41), 72 (100); HREIMS *m*/*z* 131.0721 [M+H]^+^ (calcd. for C_6_H_10_O_3_, 131.0712).

Herbidosporayilanensin I (**9**): oil.; $[\alpha {]}_{D}^{25}$= +4.7 (*c* 0.022, CHCl_3_); IR (Neat): 3424 (OH), 1772 (γ-lactone ring) cm^−1^; ^1^H NMR (500 MHz, CDCl_3_): δ_H_ 1.01 (1H, t, *J =* 7.6 Hz, H-7), 1.67 (2H, m, H-6), 2.50 (1H, dd, *J =* 18.0, 4.0 Hz, H-3β), 2.81 (1H, dd, *J =* 18.0, 6.0 Hz, H-3α), 4.27–4.31 (2H, m, H-4, 5); ^13^C NMR (125 MHz, CDCl_3_): δ_C_ 9.5 (C-7), 26.1 (C-6), 37.7 (C-3), 71.2 (C-4), 89.1 (C-5), 175.3 (C-2).; EIMS (70 eV) *m*/*z* (%): 131 ([M+H]^+^, 41), 72 (10); HREIMS *m*/*z* 131.0721 [M+H]^+^ (calcd. for C_6_H_10_O_3_, 131.0722).

### 3.4. Antitubercular Activity Assay

The in vitro antitubercular activity of each tested compound (1–7) was evaluated using *Mycobacterium tuberculosis* H_37_Rv. Middlebrook 7H10 agar was used to determine the MICs, as recommended by the proportion method [17]. Briefly, each test compound was added to Middlebrook 7H10 agar supplemented with OADC (oleic acid–albumin–dextrose–catalase) at 50–56 °C by serial dilution to yield a final concentration of 100 to 0.8 μg/mL. Ten milliliters of each concentration of test compound-containing medium was dispensed into plastic quadrant Petri dishes. Several colonies of a test isolate of *M. tuberculosis* were selected to make a suspension with Middlebrook 7H9 broth and used as the initial inoculum. The inoculum of test isolate of *M. tuberculosis* was prepared by diluting the initial inoculum in Middlebrook 7H9 broth until turbidity was reduced to the equivalent of the McFarland no. 1 standard. Final suspensions were prepared by adding Middlebrook 7H9 broth and preparing 10^−2^ dilutions of the standardized bacterial suspensions. After solidification of the Middlebrook 7H10 medium, 33 μL of the 10^−2^ dilution of the standardized bacterial suspensions was placed on each quadrant of the agar plates. The agar plates were then incubated at 35 °C with 10% CO_2_ for 2 weeks. The minimal inhibitory concentration (MIC) is the lowest concentration of test compounds that completely inhibits the growth of the test isolate of *M. tuberculosis*, as detected by the unaided eye.

## 4. Conclusions

*Herbidospora yilanensis* is a new strain isolated from the sediment of rivers in northern Taiwan. It is very novel and worthy of further study. This strain has been published in the International Journal of Systematic and Evolutionary Microbiology (IJSEM). Based on the concept of “new species and new compounds”, it is expected that special compounds will be found from these new strains. The current study was conducted on the biologically active metabolites of the EtOAc soluble fraction of by the actinomycetes *H*. *yilanensis*. This led to the isolation of five previously undescribed compounds, namely, herbidosporayilanensin A-E (**1**–**5**), and four compounds isolated from nature for the first time, namely, herbidosporayilanensins F–I (**6**–**9**) (Figure 1). The structure of these isolates was determined by spectroscopic experiments. The EtOAc soluble fraction from the *H*. *yilanensis* fermentation broth was tested in vitro against *Mycobacterium tuberculosis* strain H37Rv. See Table 3 for the anti-mycobacterial activity data. The clinically used anti-mycobacterial agent ethambutol was used as a positive control. The results of anti-mycobacterial activity showed that the herbidosporayilanensins A (**1**), B (**2**), and F (**6**) showed more effective resistance to *Mycobacterium tuberculosis* strain H37Rv in vitro with MIC values of 16.6, 19.2, and 18.2 μM, respectively, than did the clinical drug, ethambutol (MIC 6.25 μg/mL). Compounds **5** and **7** did not show anti-mycobacterial activity. When comparing the two benzene analogues (**6** and **7**), it was observed that the presence of the ketone group at position **7** of the side chain in **6** played an important role in the anti-mycobacterial activity.

In previous surveys, there have been many reports on the activity of actinomycete metabolites in the literature, but there are some reports of active natural products against *Mycobacterium tuberculosis* in vitro. Therefore, it is still worth continuing to study the active substance against different *Mycobacterium tuberculosis* strains.

## Figures and Tables

**Table 1:** ^1^H NMR data for Compounds **1**–**7** in CDCl_3_ (*δ* in ppm, *J* in Hz, 500 MHz in CDCl_3_).

No	1	2	3	4	5	6	7
1	1.12 (m) 1.79 (m)	1.11 (m) 1.63 (m)	1.05 (d, *J =* 3.4) 1.74 (m)	2.67 (d, *J =* 13.7, H-α) 2.90 (dd, *J =* 13.7, 2.0, H-β)	1.12 (m) 1.80 (m)		
2	1.51 (m) 1.83 (m)	1.46 (m) 1.77 (m)	1.45 (td, *J =* 13.4, 3.4) 1.63 (m)		1.51 (m) 1.82 (m)	7.40 (d, *J =* 2.0)	6.64 (d, *J =* 1.8)
3	1.04 (m) 2.13 (m)	1.06 (m) 2.11 (m)	1.35 (m) 1.77 (m)	2.38 (dd, *J =* 13.1, 2.0, H-β) 2.43 (d, *J =* 13.1, H-α)	1.05 (m) 2.14 (m)		
4							
5	1.38 (dd, *J =* 12.0, 2.6)	2.04 (dd, *J =* 12.6, 3.3)	1.37 (m)	2.42 (dd, *J =* 13.7, 3.8)	1.38 (dd, *J =* 12.5, 2.6)	6.88 (d, *J =* 8.0)	6.79 (d, *J =* 8.1)
6	1.86 (m) 2.00 (m)	1.87 (m) 1.93 (m)	1.36 (m) 1.95 (m)	2.64 (dd, *J =* 17.9, 13.7, H-β) 2.79 (d, *J =* 17.9, 3.8, H-α)	1.86 (m) 1.97 (m)	7.48 (dd, *J =* 8.0, 2.0)	6.67 (dd, *J =* 8.1, 1.8
7	1.94 (m) 2.39 (m)	2.29 (m) 2.49 (m)	1.98 (m) 2.41 (m)		1.95 (m) 2.39 (m)		2.70 (m)
8						4.27 (m)	2.81 (m)
9	1.98 (m)		1.64 (m)		1.99 (m)	4.43 (dd, *J =* 9.2, 6.8) 4.57 (t, *J =* 9.2)	4.02 (dd, *J =* 9.1, 6.0) 4.31 (dd, *J =* 9.1, 7.0)
10						2.75 (dd, *J =* 17.6, 9.2) 3.00 (dd, *J =* 17.6, 7.6)	2.27(dd, *J =* 17.5, 6.9) 2.58 (dd, *J =* 17.5, 8.2)
11	1.42 (m) 1.67 (m)	7.17 (d, *J =* 15.8)	1.52 (m) 1.64 (m)	6.57 (s)	1.52 (m) 1.55 (m)		
12	3.56 (d, *J =* 10.5)	6.43 (d, *J =* 15.8)	1.99 (m) 2.31 (m)	6.37 (d, *J =* 16.0)	3.99 (t, *J =* 5.6)		
OH-12				6.18 (br s)			
13							
14	4.08 (M)	2.28 (s)	5.66 (s)	7.92 (*s*)	5.65 (br s)		
15	3.92 (m) 3.99 (m)			3.15 (sep, *J =* 6.8)	2.19 (m)		
16	1.25 (s)		2.15 (s)	1.25 (d, *J =* 6.8)	2.56 (m)		
17	4.53 (br. s) 4.84 (br s)		4.51 (br s) 4.87 (br s)	1.25 (d, *J =* 6.8)	4.44 (br s) 4.84 (br s)		
18	1.22 (s)	1.27 (s)		1.11 (s)	1.22 (s)		
19			1.10 (s)	1.01 (s)			
20	0.59 (s)	0.89 (s)	0.64 (s)	1.23 (s)	0.57 (s)		
21	1.39 (s)				2.07 (m) 2.12 (m)		
22					1.57 (m) 2.00 (m)		
23	1.35 (s)						
24					2.16 (s)		
OCH_2_O						6.07 (s)	
OMe-3							3.85 (s)
OMe-4							3.85 (s)

**Table 2:** ^13^C NMR data for Compounds **1**–**7** (*δ* in ppm, 125 MHz for ^13^C NMR in CDCl_3_).

No	1	2	3	4	5	6	7
1	39.0	32.8	38.2	53.2	39.1	129.8	130.7
2	19.9	19.2	20.5	209.2	19.8	108.1	111.8
3	37.9	37.6	42.9	56.0	37.9	148.8	149.1
4	44.2	44.1	72.5	38.7	44.1	152.8	147.9
5	56.2	74.4	56.9	49.0	56.2	108.3	111.4
6	26.0	24.2	23.4	35.9	26.0	124.9	120.0
7	38.7	32.5	37.8	196.9	38.6	194.1	38.6
8	148.0	149.5	147.6	124.0	148.5	41.9	37.3
9	51.6	79.9	55.9	153.5	51.8	69.2	72.6
10	40.1	43.1	40.3	42.8	40.1	31.1	34.2
11	25.4	148.9	21.8	109.6	30.5	175.3	176.8
12	74.4	130.2	40.0	158.9	73.9		
13	73.9	197.8	163.7	133.8	140.9		
14	79.5	28.2	114.3	126.9	119.4		
15	65.1		169.8	26.8	26.5		
16	20.7		19.2	22.2	47.3		
17	107.0		107.1	22.4	106.6		
18	29.0	29.0		32.4	29.0		
19	182.6	183.1	23.1	22.5	182.6		
20	12.9	16.5	13.9	24.3	12.9		
21	26.4				23.9		
22	108.4				24.6		
23	25.4				211.4		
24					28.0		
OCH_2_O						102.2	
OMe-3							55.9
OMe-4							55.89

**Table 3:** Antimycobacterial effects of some isolates (**1**–**7**) from the cultures of one actinobacterium harbored in *H*. *yilanensis* on *M. tuberculosis* H37Rv.

Compounds	MIC (μM) ^a^
herbidosporayilanensin A (**1**)	16.6
herbidosporayilanensin B (**2**)	19.2
herbidosporayilanensin C (**3**)	40.8
herbidosporayilanensin D (**4**)	50.6
herbidosporayilanensin E (**5**)	>621
herbidosporayilanensin F (**6**)	18.2
herbidosporayilanensin G (**7**)	>543
Ethambutol ^b^	30.6

^a^ The minimal inhibitory concentration (MIC) is the lowest concentration of test compounds that completely inhibits the growth of the test isolate of *M*. *tuberculosis*. The MIC values represent the average of three independent experiments. ^b^ Positive control.

## Data Availability

The data presented in this study are available in the article and Supplementary Material.

## Supplementary Materials

The following are available online, Figure S1. 1H NMR spectrum of 1; Figure S2. 13C NMR spectrum of 1; Figure S3. 1H-1H COSY spectrum of 1; Figure S4. HMBC spectrum of 1; Figure S5. NOESY spectrum of 1; Figure S6. HSQC spectrum of 1; Figure S7. EI-MS spectrum of 1; Figure S8. 1H NMR spectrum of 2; Figure S9. 13C NMR spectrum of 2; Figure S10. 1H-1H COSY spectrum of 2; Figure S11. HMBC spectrum of 2; Figure S12. NOESY spectrum of 2; Figure S13. HSQC spectrum of 2; Figure S14. EI-MS spectrum of 2; Figure S15. 1H NMR spectrum of 3; Figure S16. 13C NMR spectrum of 3; Figure S17. 1H-1H COSY spectrum of 3; Figure S18. HMBC spectrum of 3; Figure S19. NOESY spectrum of 3; Figure S20. HSQC spectrum of 3; Figure S21. EI-MS spectrum of 3; Figure S22. 1H NMR spectrum of 4; Figure S23. 13C NMR spectrum of 4; Figure S24. 1H-1H COSY spectrum of 4; Figure S25. HMBC spectrum of 4; Figure S26. NOESY spectrum of 4; Figure S27. HSQC spectrum of 4; Figure S28. EI-MS spectrum of 4; Figure S29. 1H NMR spectrum of 5; Figure S30. 13C NMR spectrum of 5; Figure S31. 1H-1H COSY spectrum of 5; Figure S32. HMBC spectrum of 5; Figure S33. NOESY spectrum of 5; Figure S34. HSQC spectrum of 5; Figure S35. EI-MS spectrum of 5; Figure S36. 1H NMR spectrum of 6; Figure S37. 13C NMR/DEPT spectra of 6; Figure S38. 1H-1H COSY spectrum of 6; Figure S39. HMBC spectrum of 6; Figure S40. NOESY spectrum of 6; Figure S41. HSQC spectrum of 6; Figure S42. EI-MS spectrum of 6; Figure S43. 1H NMR spectrum of 7; Figure S44. 13C NMR/DEPT spectra of 7; Figure S45. 1H-1H COSY spectrum of 7; Figure S46. HMBC spectrum of 7; Figure S47. NOESY spectrum of 7; Figure S48. HSQC spectrum of 7; Figure S49. EI-MS spectrum of 7; Figure S50. 1H NMR spectrum of 8; Figure S51. 13C NMR/DEPT spectra of 8; Figure S52. 1H-1H COSY spectrum of 8; Figure S53. HMBC spectrum of 8; Figure S54. NOESY spectrum of 8; Figure S55. HSQC spectrum of 8; Figure S56. EI-MS spectrum of 8; Figure S57. 1H NMR spectrum of 9; Figure S58. 13C NMR/DEPT spectra of 9; Figure S59. 1H-1H COSY spectrum of 9; Figure S60. HMBC spectrum of 9; Figure S61. NOESY spectrum of 9; Figure S62. HSQC spectrum of 9; Figure S63. EI-MS spectrum of 9.
