# Supplementary material for: Secondary Metabolites with Antimycobacterial Activities from One Actinobacteria: Herbidospora yilanensis
Source: Molecules. 2021 Oct 15;26(20):6236. doi: 10.3390/molecules26206236 (PMC8570327; doi:10.3390/molecules26206236)
Supplement: Supplementary file 1 [file molecules-26-06236-s001.zip › molecules-1391374-supply.pdf]

Secondary metabolites with Antimycobacterial Activities from one Actinobacteria *Herbidospora yilanensis*

Ming-Jen Cheng[1*], Mind-Der Wu[1†], Jih-Jung Chen[2,3*†], Yung-Shun Su[4,5], and Yueh-Hsiung Kuo[3,6,7,8†]

[1] Bioresource Collection and Research Center (BCRC), Food Industry Research and Development Institute (FIRDI), Hsinchu 300, Taiwan, e-mail: chengfirdi@gmail.com
[2] Department of Pharmacy, School of Pharmaceutical Sciences, National Yang Ming Chiao Tung University (NYCU), Taipei 112, Taiwan, e-mail: jjungchen@nycu.edu.tw
[3] Department of Medical Research, China Medical University Hospital, Taichung 404, Taiwan
[4] Graduate Institute of Medicine, College of Medicine, Kaohsiung Medical University (KMU), Kaohsiung 807, Taiwan, e-mail: mariussu@gmail.com
[5] Department of Dermatology, Kaohsiung Medical University Chung-Ho Memorial Hospital, Kaohsiung 807, Taiwan
[6] Department of Chemistry, National Taiwan University, Taipei 106, Taiwan
[7] Department of Biotechnology, Asia University, Taichung 413, Taiwan
[8] Department of Chinese Pharmaceutical Sciences and Chinese Medicine Resources, College of Pharmacy, China Medical University, Taichung 404, Taiwan

[†] Equal contribution as correspondence
*Correspondences: chengfirdi@gmail.com (M.-J.C.); jjungchen@nycu.edu.tw (J.-J. Chen)

Contents

Figure S1. ^{1}H NMR spectrum of **1** 5
Figure S2. ^{13}C NMR spectrum of **1** 5
Figure S3. ^{1}H-^{1}H COSY spectrum of **1** 6
Figure S4. HMBC spectrum of **1** 6
Figure S5. NOESY spectrum of **1** 7
Figure S6. HSQC spectrum of **1** 7
Figure S7. EI-MS spectrum of **1** 8

Figure S8. ^{1}H NMR spectrum of **2** 9
Figure S9. ^{13}C NMR spectrum of **2** 9
Figure S10. ^{1}H-^{1}H COSY spectrum of **2** 10
Figure S11. HMBC spectrum of **2** 10
Figure S12. NOESY spectrum of **2** 11
Figure S13. HSQC spectrum of **2** 11
Figure S14. EI-MS spectrum of **2** 12

Figure S15. ^{1}H NMR spectrum of **3** 13
Figure S16. ^{13}C NMR spectrum of **3** 13
Figure S17. ^{1}H-^{1}H COSY spectrum of **3** 14
Figure S18. HMBC spectrum of **3** 14
Figure S19. NOESY spectrum of **3** 15
Figure S20. HSQC spectrum of **3** 15
Figure S21. EI-MS spectrum of **3** 16

Figure S22. ^{1}H NMR spectrum of **4** 17

Figure S23. ^{13}C NMR spectrum of **4** 17
Figure S24. ^{1}H-^{1}H COSY spectrum of **4** 18
Figure S25. HMBC spectrum of **4** 18
Figure S26. NOESY spectrum of **4** 19
Figure S27. HSQC spectrum of **4** 19
Figure S28. EI-MS spectrum of **4** 20

Figure S29. ^{1}H NMR spectrum of **5** 21
Figure S30. ^{13}C NMR spectrum of **5** 21
Figure S31. ^{1}H-^{1}H COSY spectrum of **5** 22
Figure S32. HMBC spectrum of **5** 22
Figure S33. NOESY spectrum of **5** 23
Figure S34. HSQC spectrum of **5** 23
Figure S35. EI-MS spectrum of **5** 24

Figure S36. ^{1}H NMR spectrum of **6** 25
Figure S37. ^{13}C NMR/DEPT spectra of **6** 25
Figure S38. ^{1}H-^{1}H COSY spectrum of **6** 26
Figure S39. HMBC spectrum of **6** 26
Figure S40. NOESY spectrum of **6** 27
Figure S41. HSQC spectrum of **6** 27
Figure S42. EI-MS spectrum of **6** 28

Figure S43. ^{1}H NMR spectrum of **7** 29
Figure S44. ^{13}C NMR/DEPT spectra of **7** 29
Figure S45. ^{1}H-^{1}H COSY spectrum of **7** 30
Figure S46. HMBC spectrum of **7** 30

Figure S47. NOESY spectrum of **7** 31
Figure S48. HSQC spectrum of **7** 31
Figure S49. EI-MS spectrum of **7** 32

Figure S50. ^{1}H NMR spectrum of **8** 33
Figure S51. ^{13}C NMR/DEPT spectra of **8** 33
Figure S52. ^{1}H-^{1}H COSY spectrum of **8** 34
Figure S53. HMBC spectrum of **8** 34
Figure S54. NOESY spectrum of **8** 35
Figure S55. HSQC spectrum of **8** 35
Figure S56. EI-MS spectrum of **8** 36

Figure S57. ^{1}H NMR spectrum of **9** 37
Figure S58. ^{13}C NMR/DEPT spectra of **9** 37
Figure S59. ^{1}H-^{1}H COSY spectrum of **9** 38
Figure S60. HMBC spectrum of **9** 38
Figure S61. NOESY spectrum of **9** 39
Figure S62. HSQC spectrum of **9** 39
Figure S63. EI-MS spectrum of **9** 40

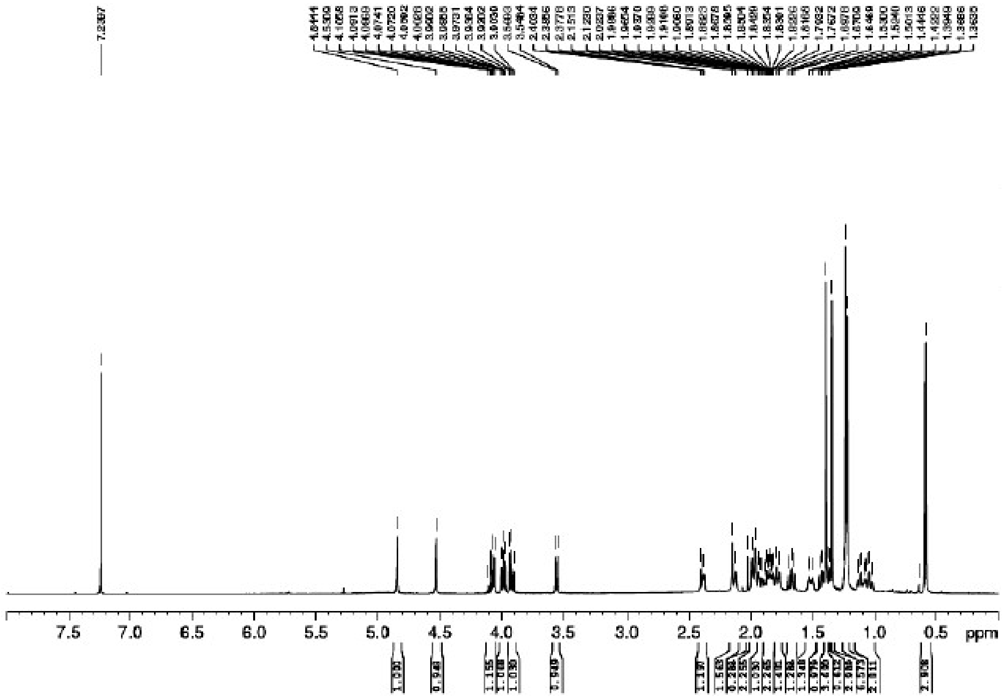

Figure S1. ^{1}H NMR spectrum of **1**

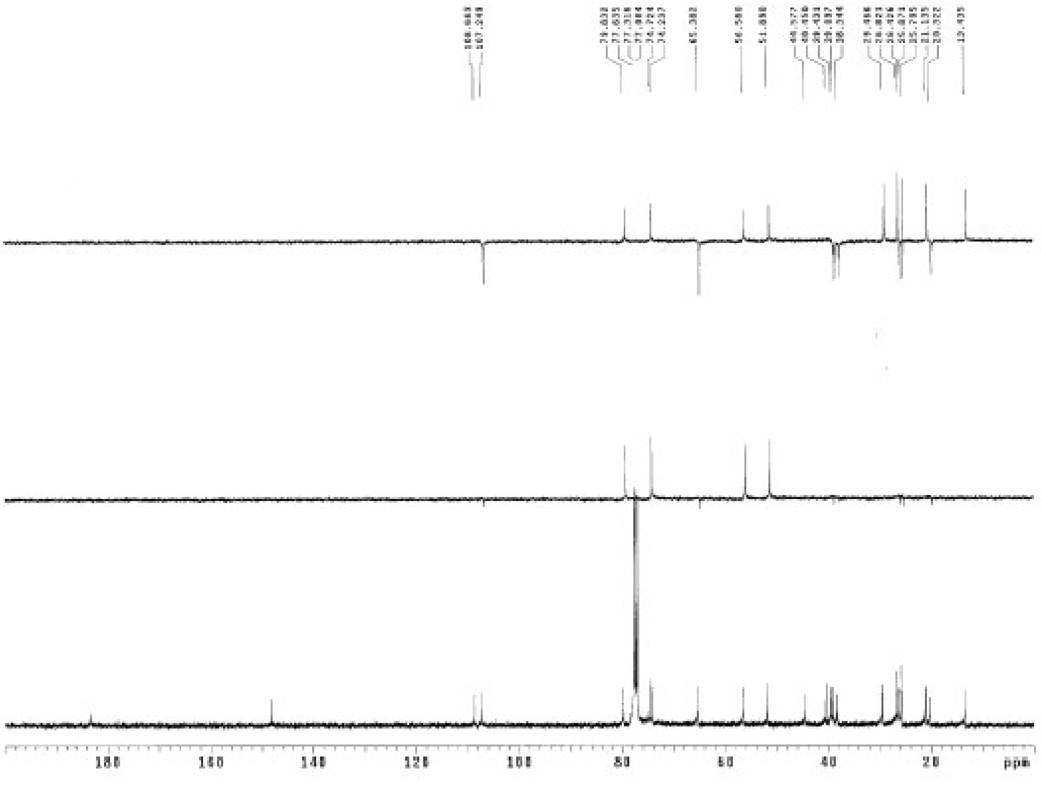

Figure S2. ^{13}C NMR spectrum of **1**

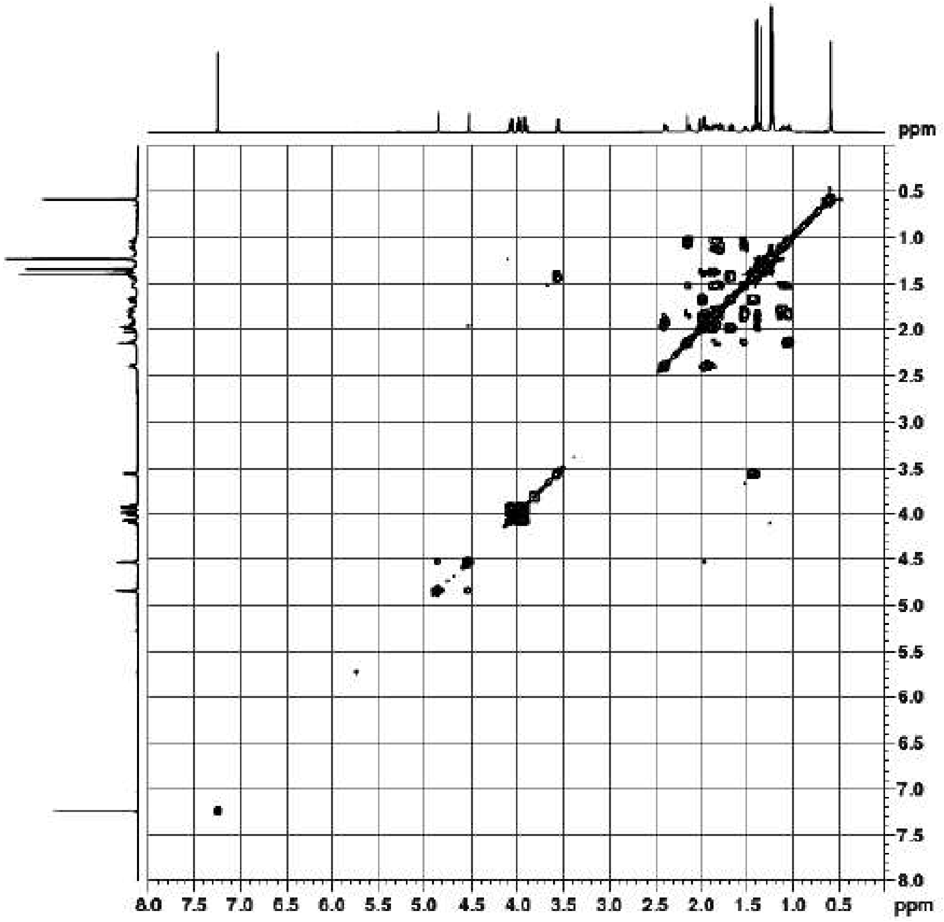

Figure S3. COSY spectrum of **1**

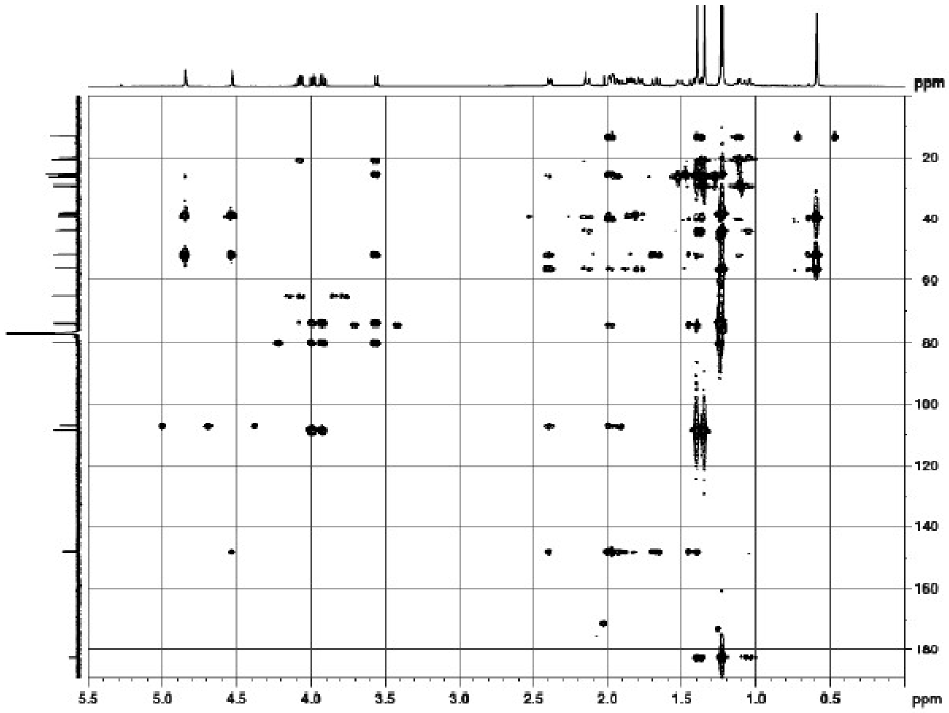

Figure S4. HMBC spectrum of **1**

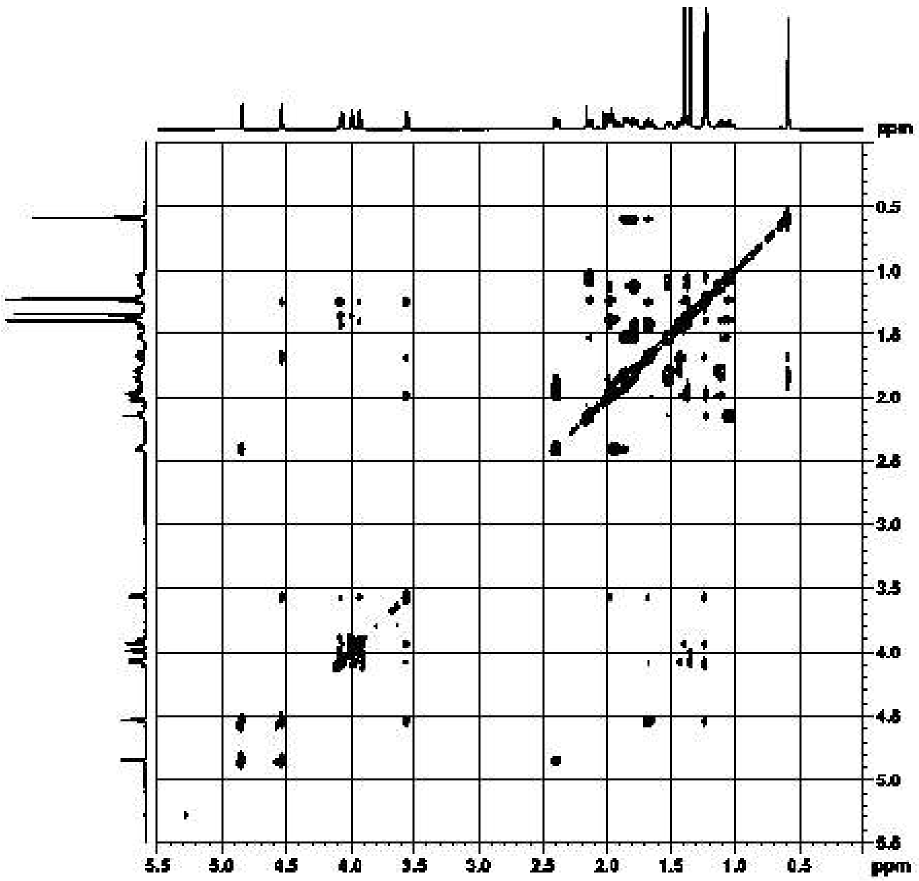

Figure S5. NOESY spectrum of **1**

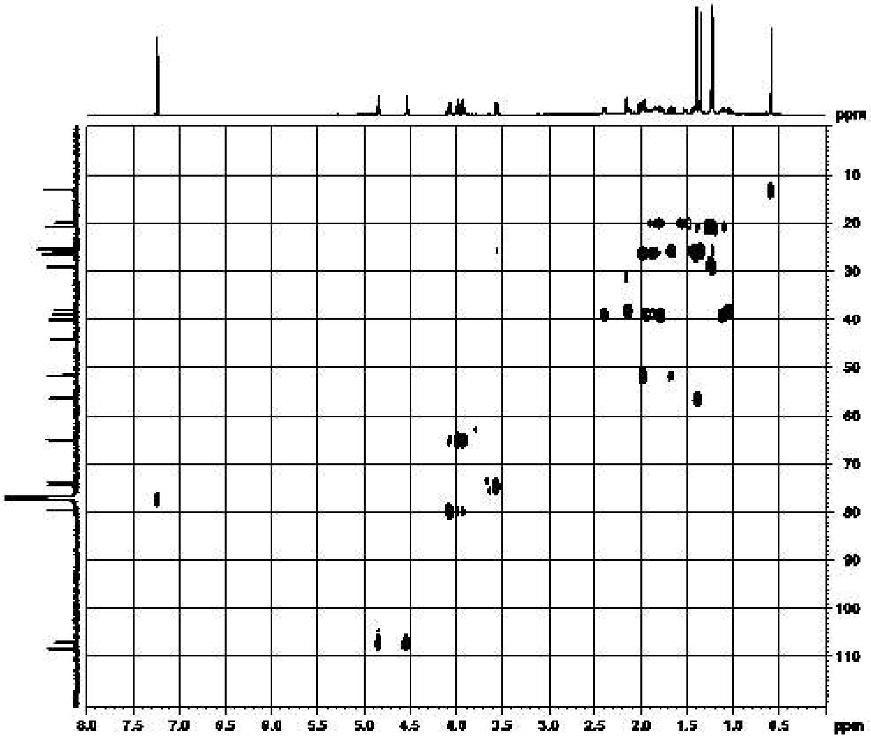

Figure S6. HSQC spectrum of **1**

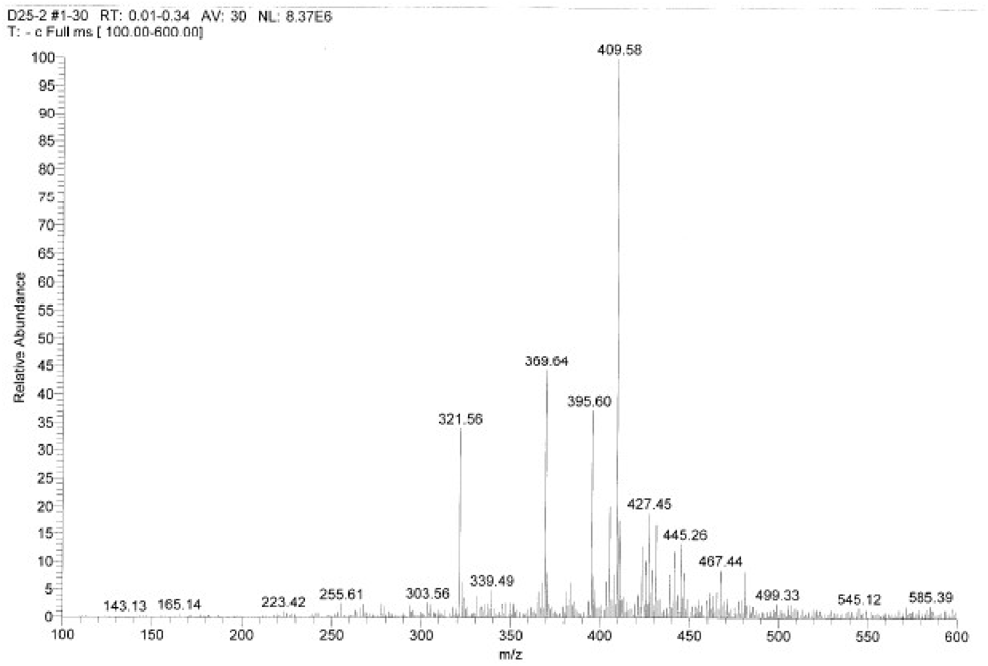

Figure S7. EIMS spectrum of **1**

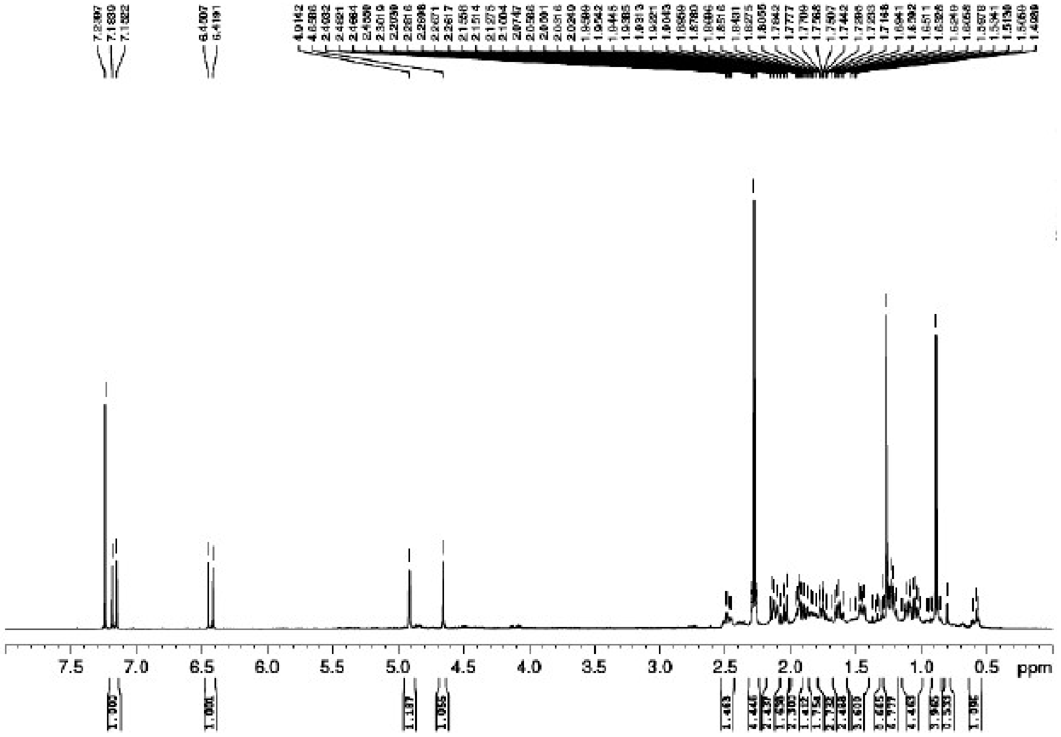

Figure S8. ^{1}H NMR spectrum of **2**

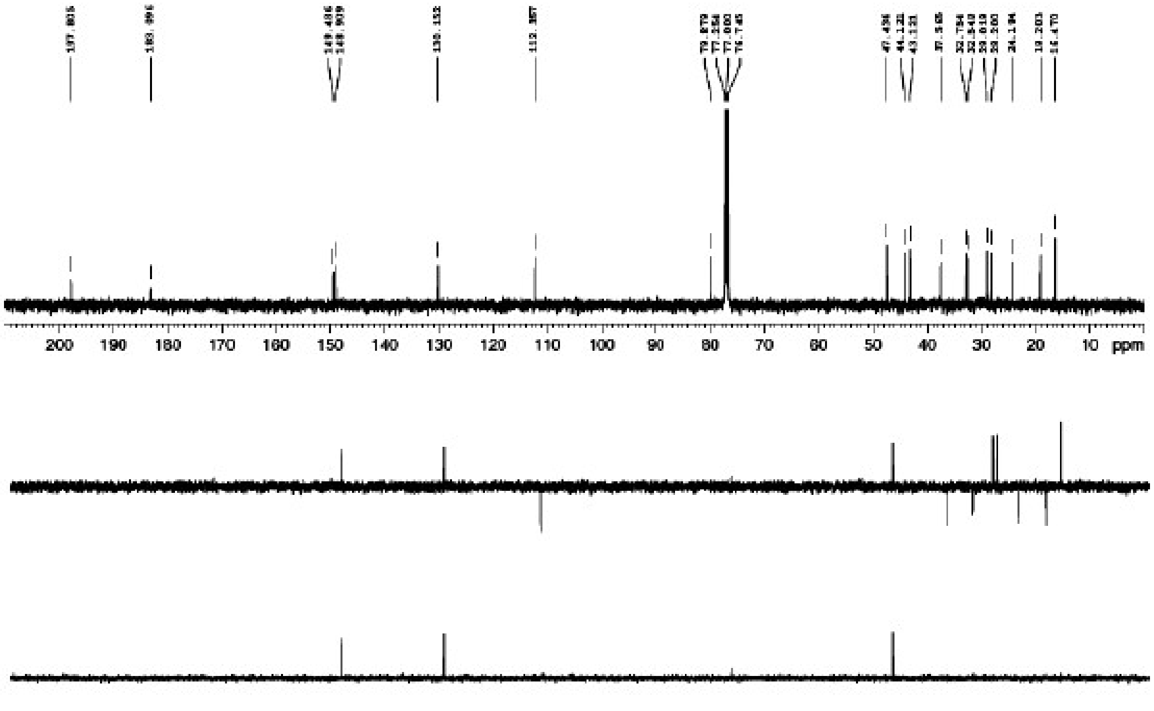

Figure S9. ^{13}C spectrum of **2**

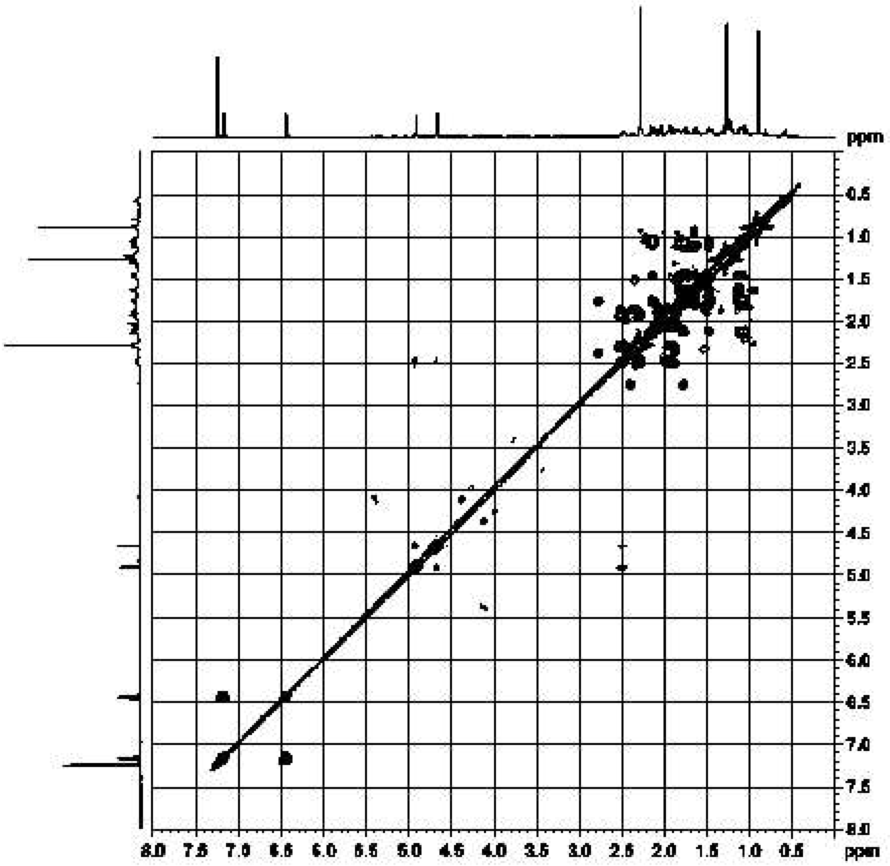

Figure S10. COSY spectrum of **2**

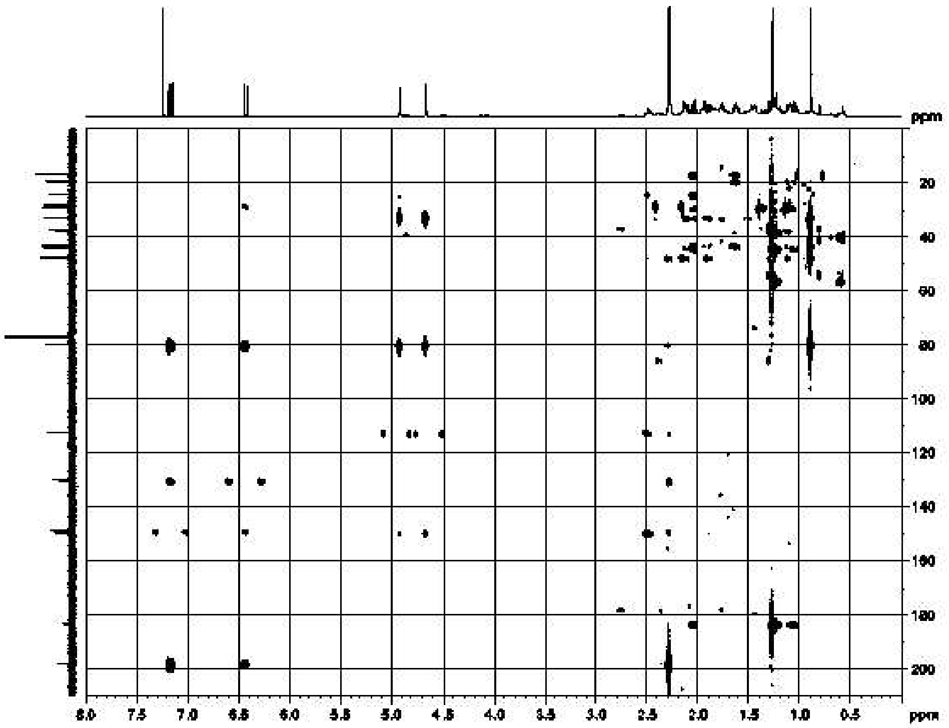

Figure S11. HMBC spectrum of **2**

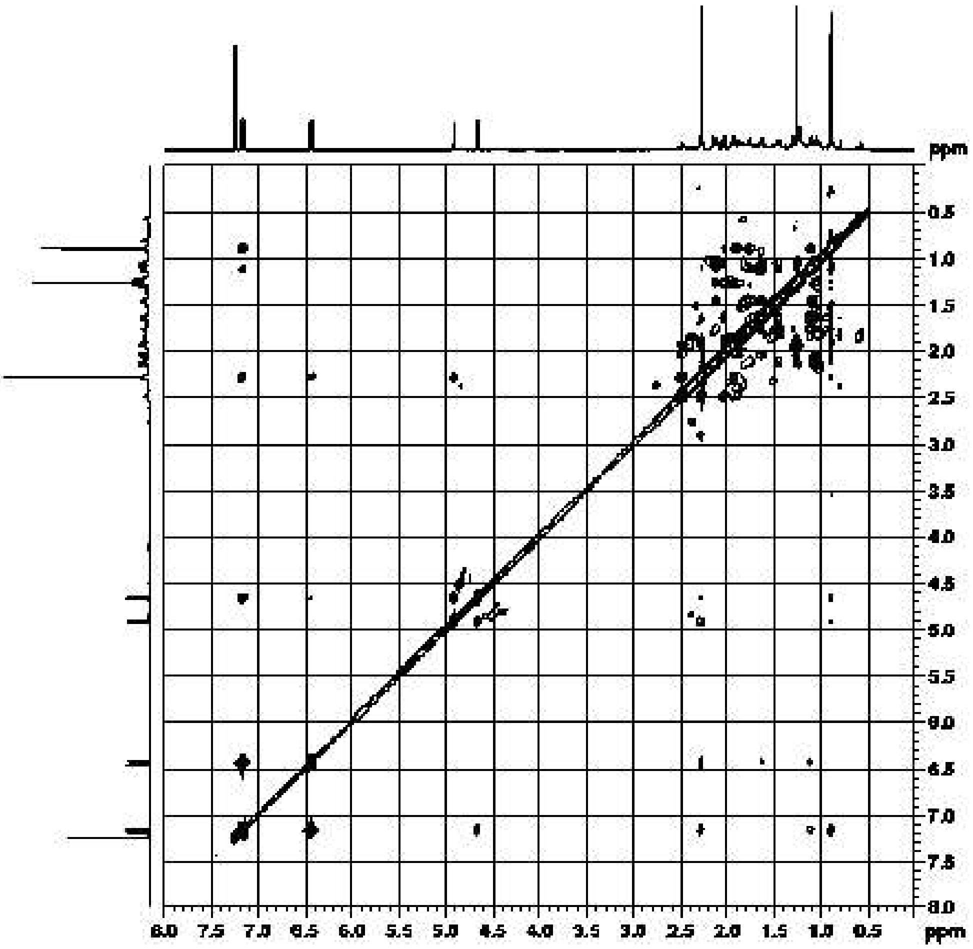

Figure S12. NOESY spectrum of **2**

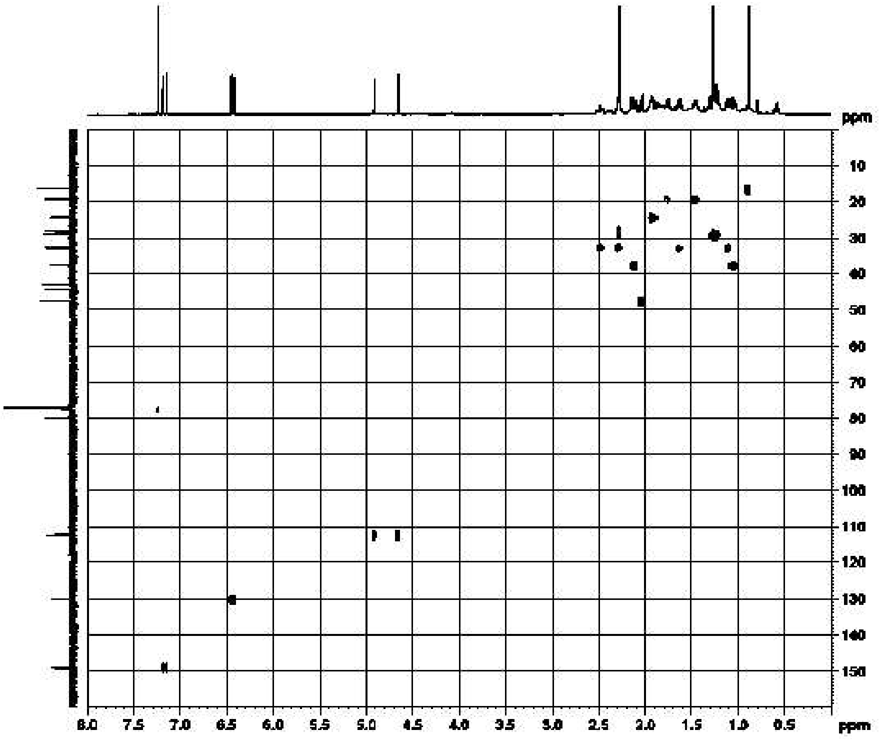

Figure S13. HSQC spectrum of **2**

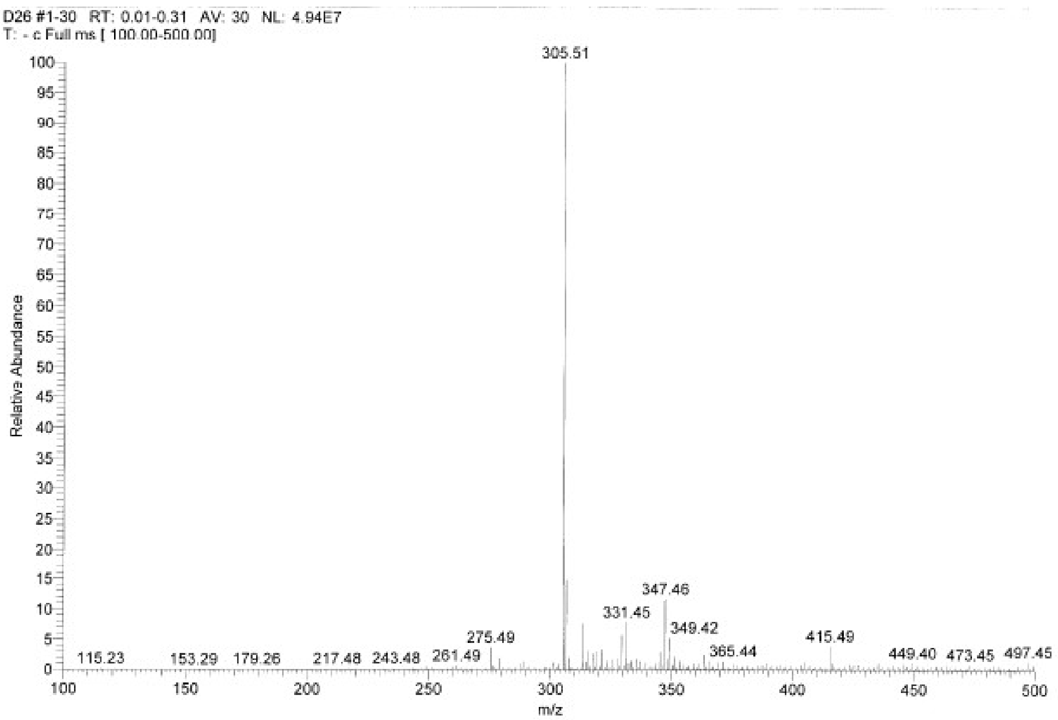

Figure S14. EI-MS spectrum of **2**

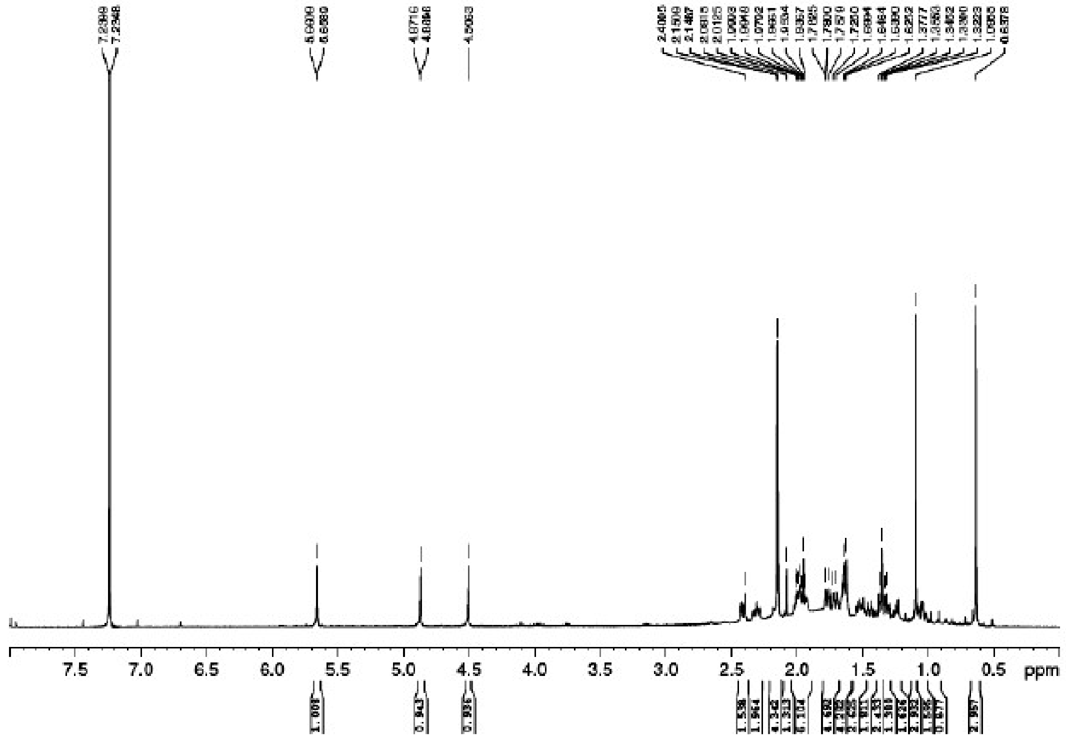

Figure S15. ^{1}H NMR spectrum of **3**

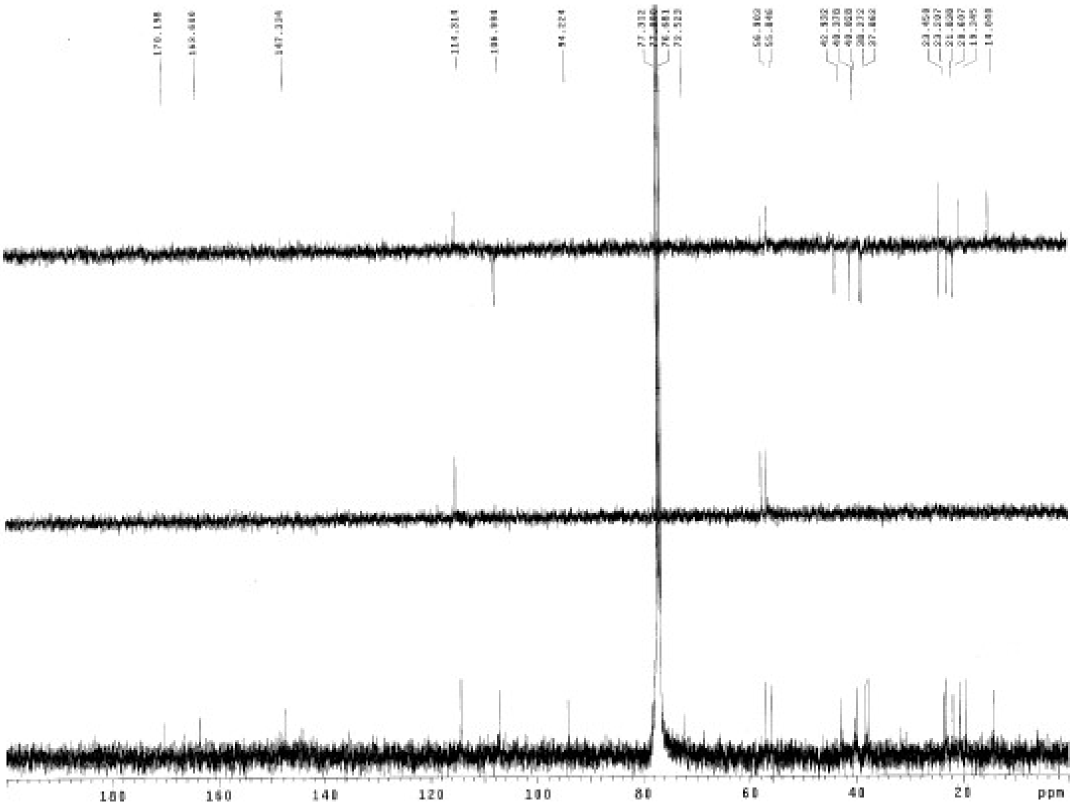

Figure S16. ^{13}C NMR spectrum of **3**

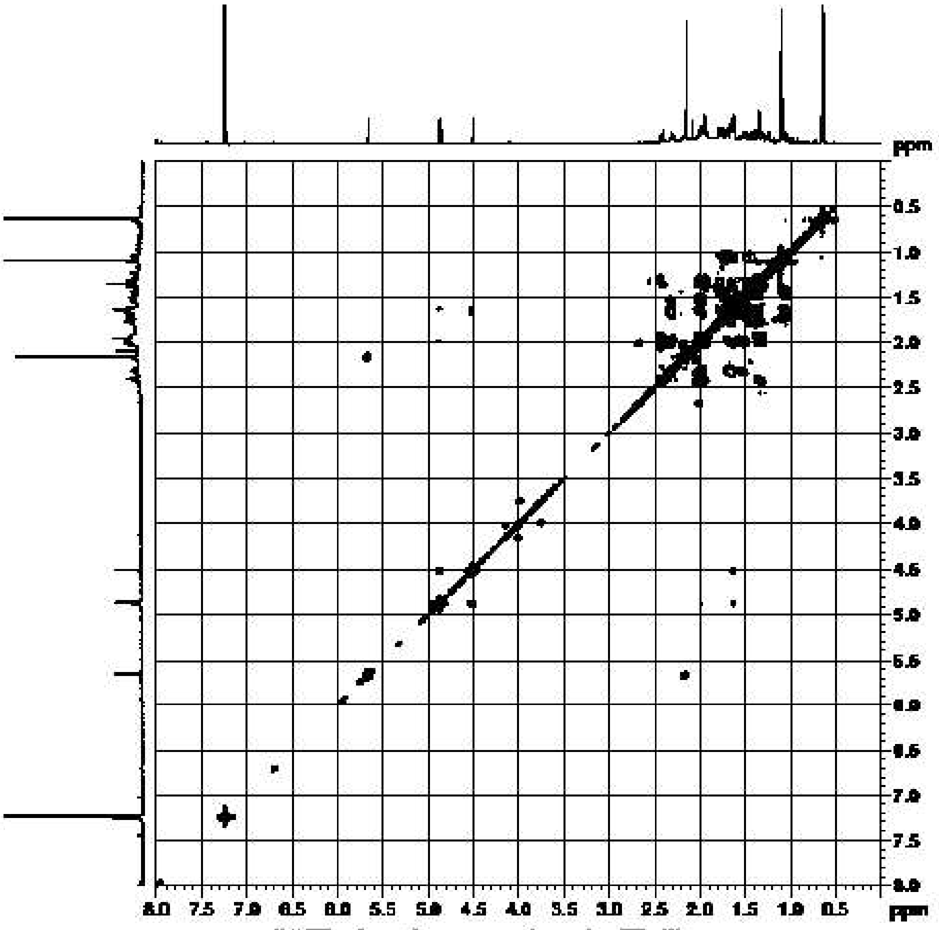

Figure S17. COSY spectrum of **3**

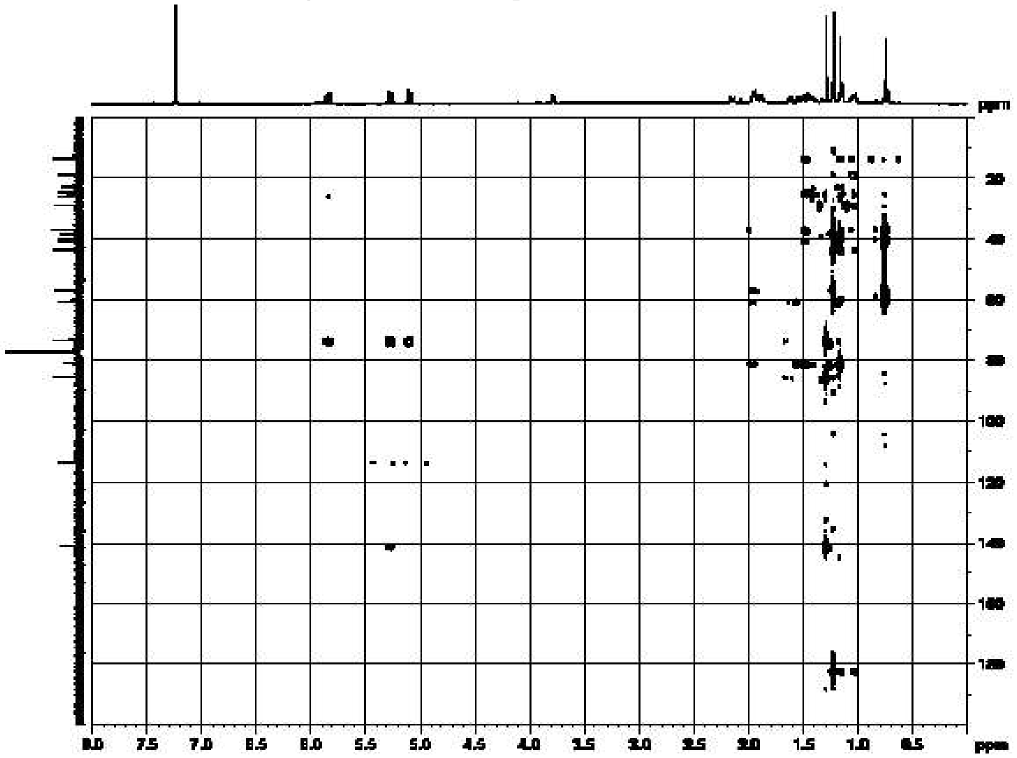

Figure S18. HMBC spectrum of **3**

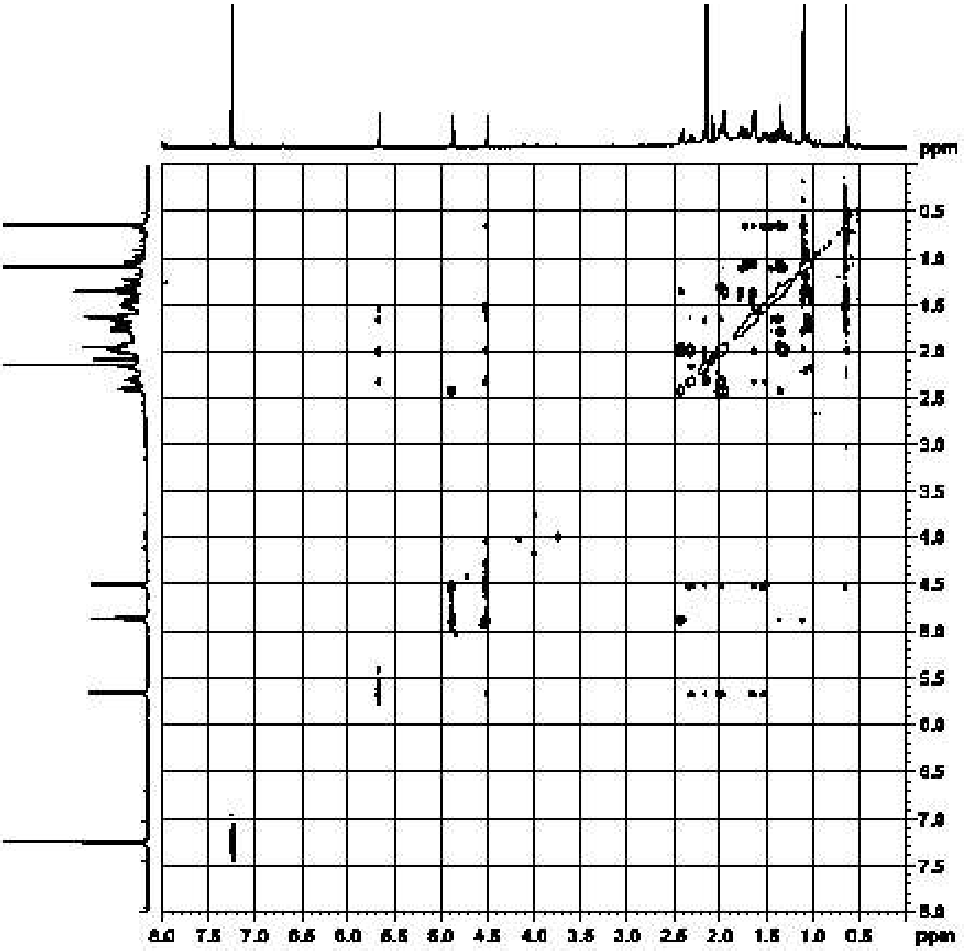

Figure S19. NOESY spectrum of **3**

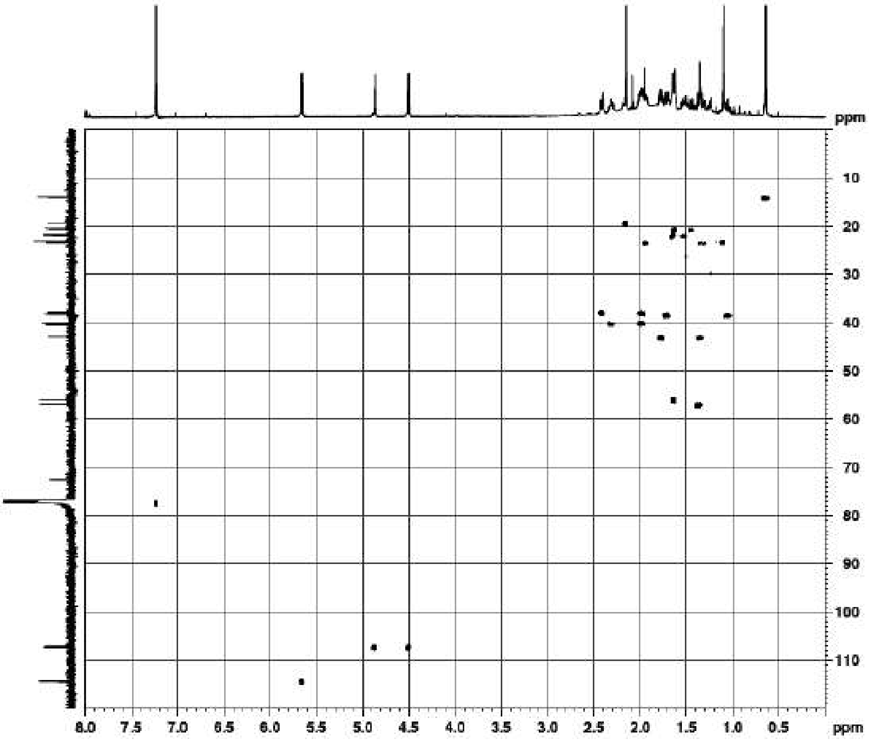

Figure S20. HSQC spectrum of **3**

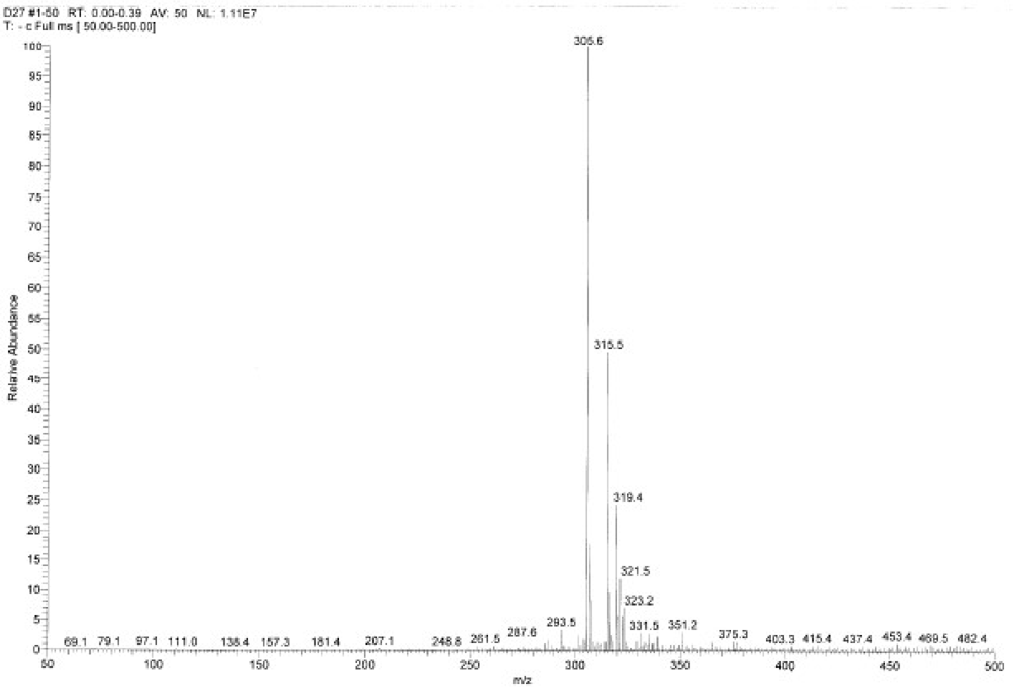

Figure S21. EI-MS spectrum of **3**

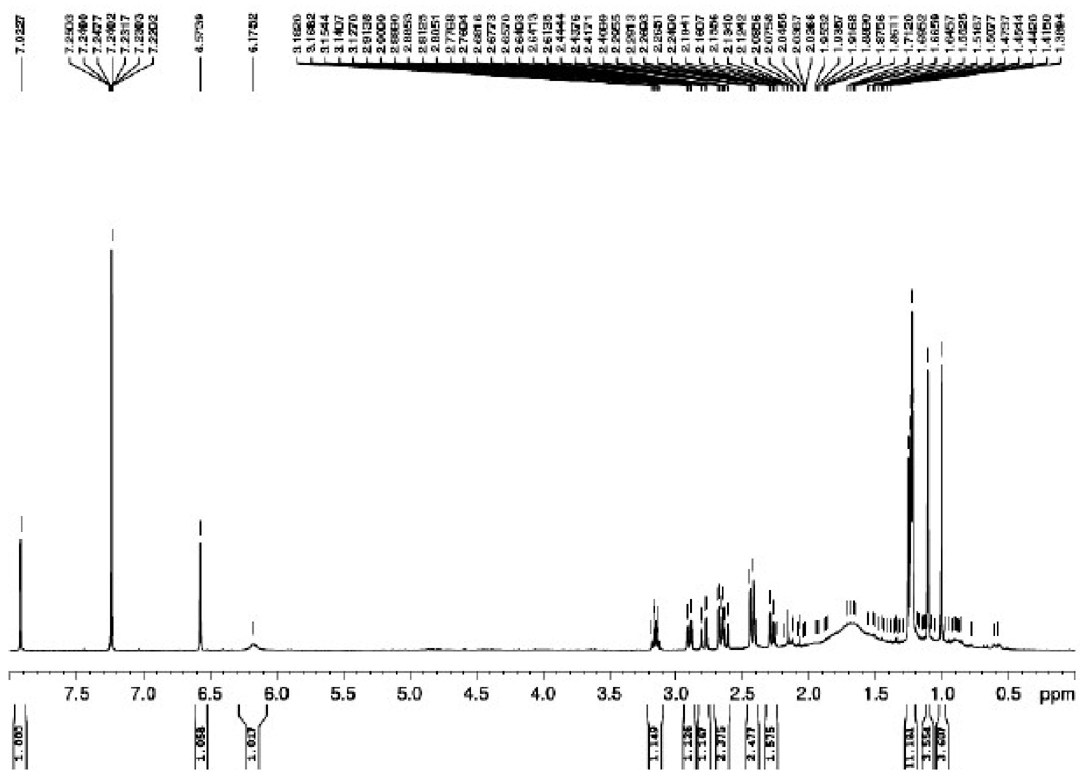

Figure S22. ^{1}H NMR spectrum of **4**

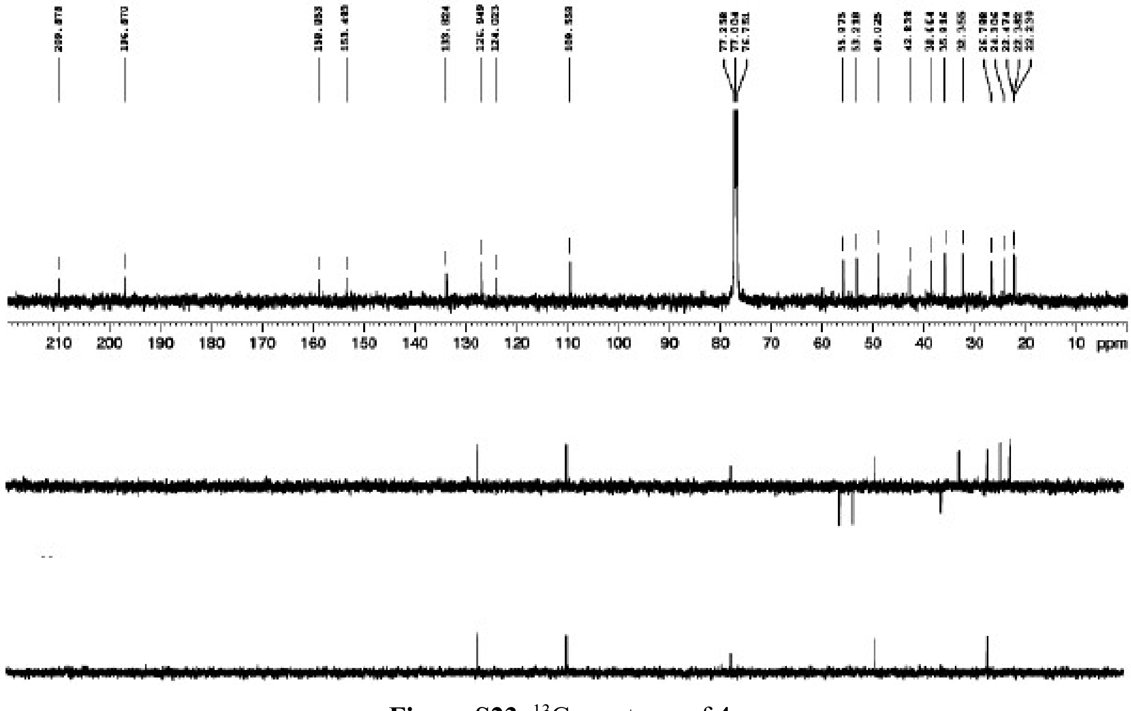

Figure S23. ^{13}C spectrum of **4**

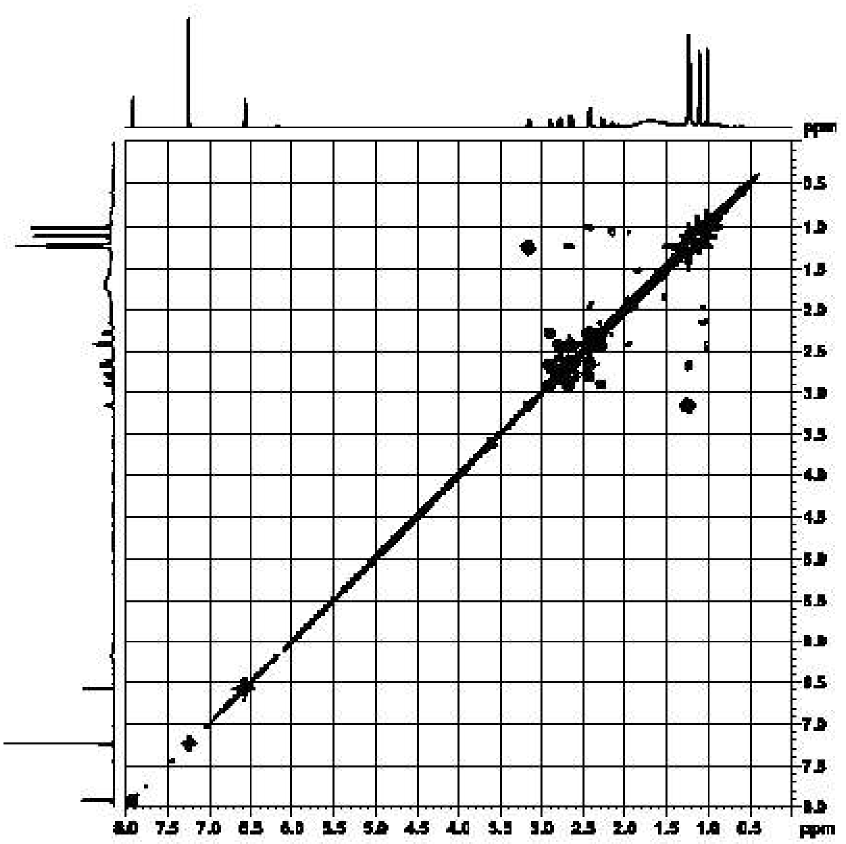

Figure S24. COSY spectrum of **4**

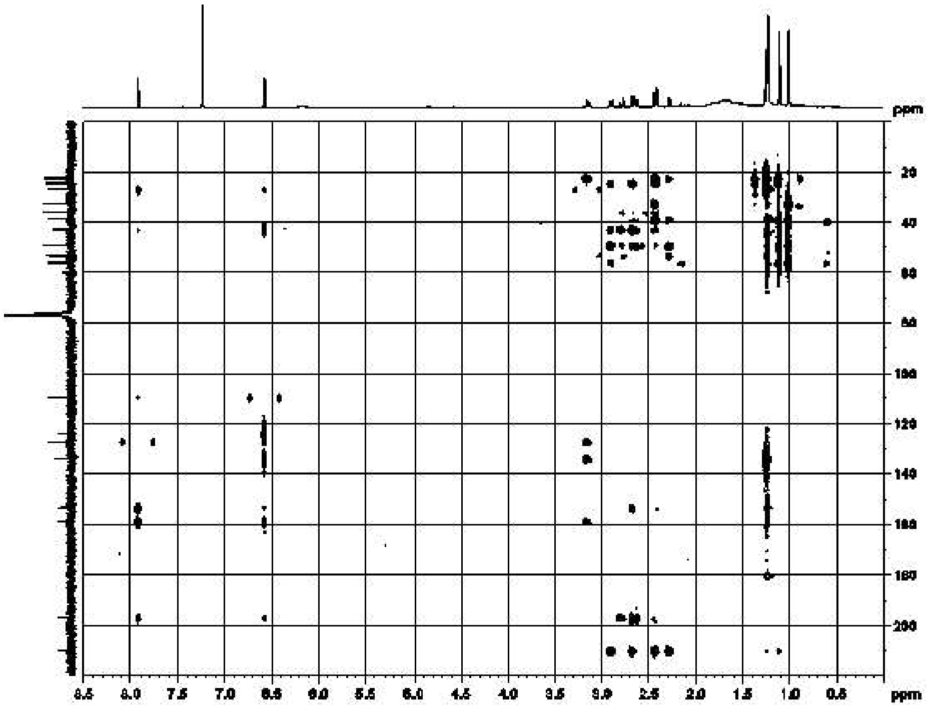

Figure S25. HMBC spectrum of **4**

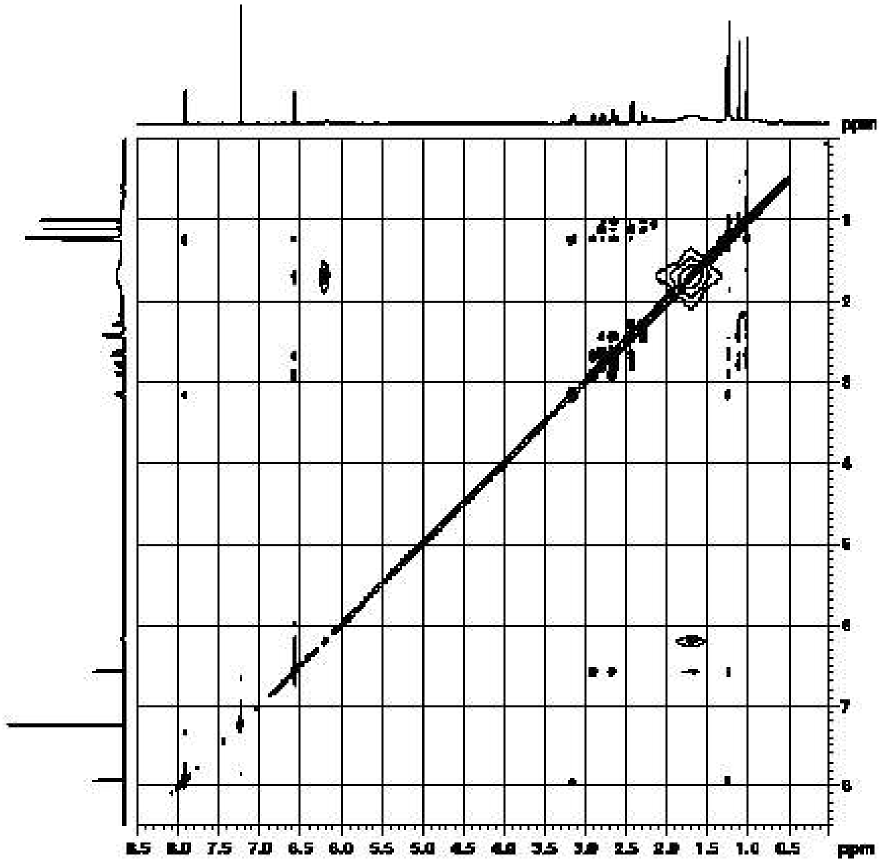

Figure S26. NOESY spectrum of 4

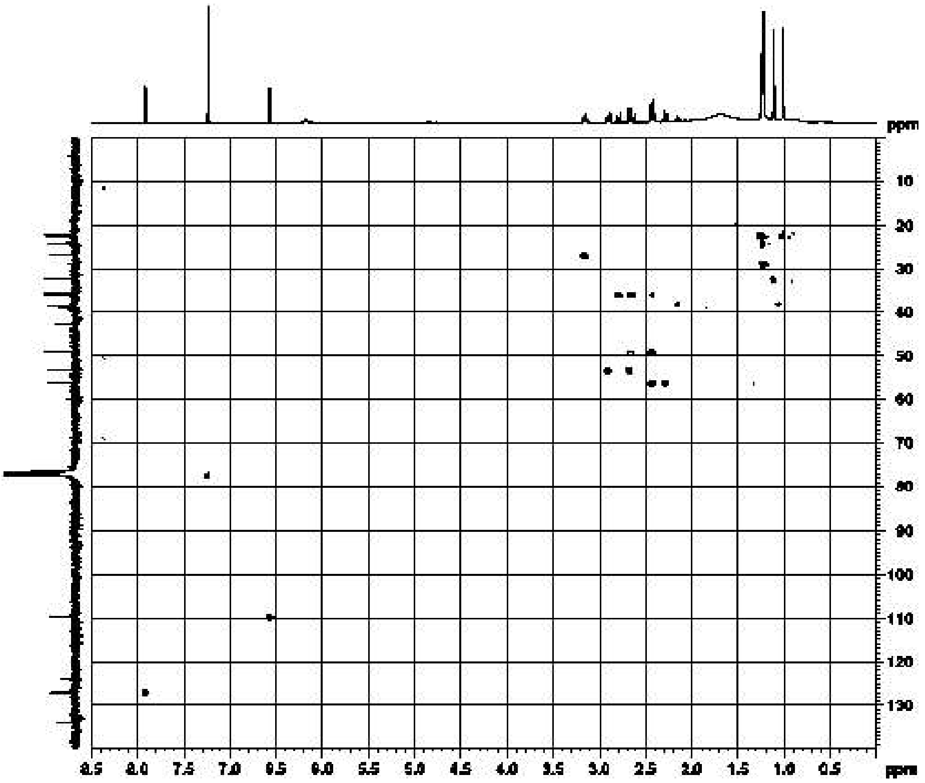

Figure S27. HSQC spectrum of 4

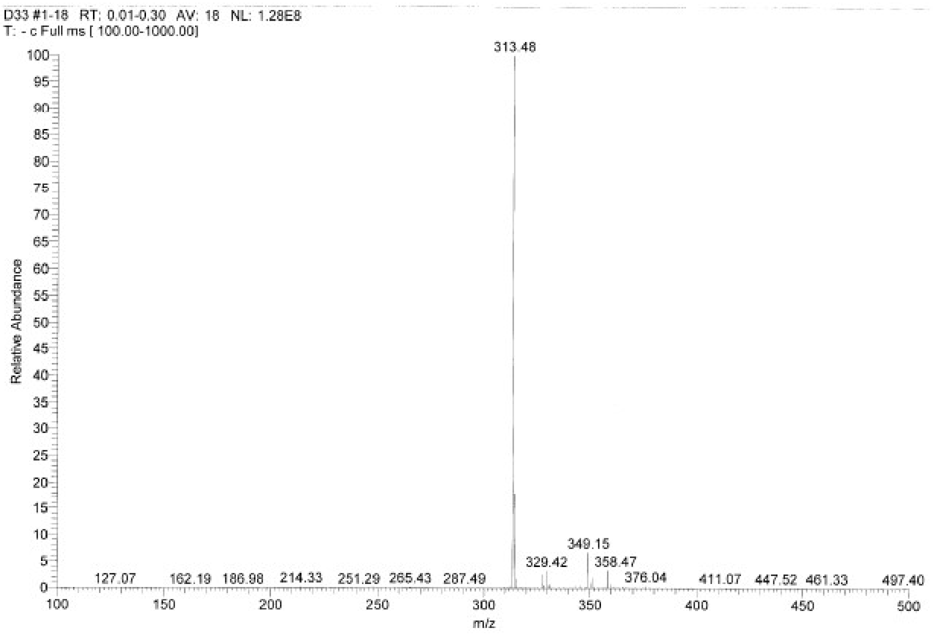

Figure S28. EI-MS spectrum of **4**

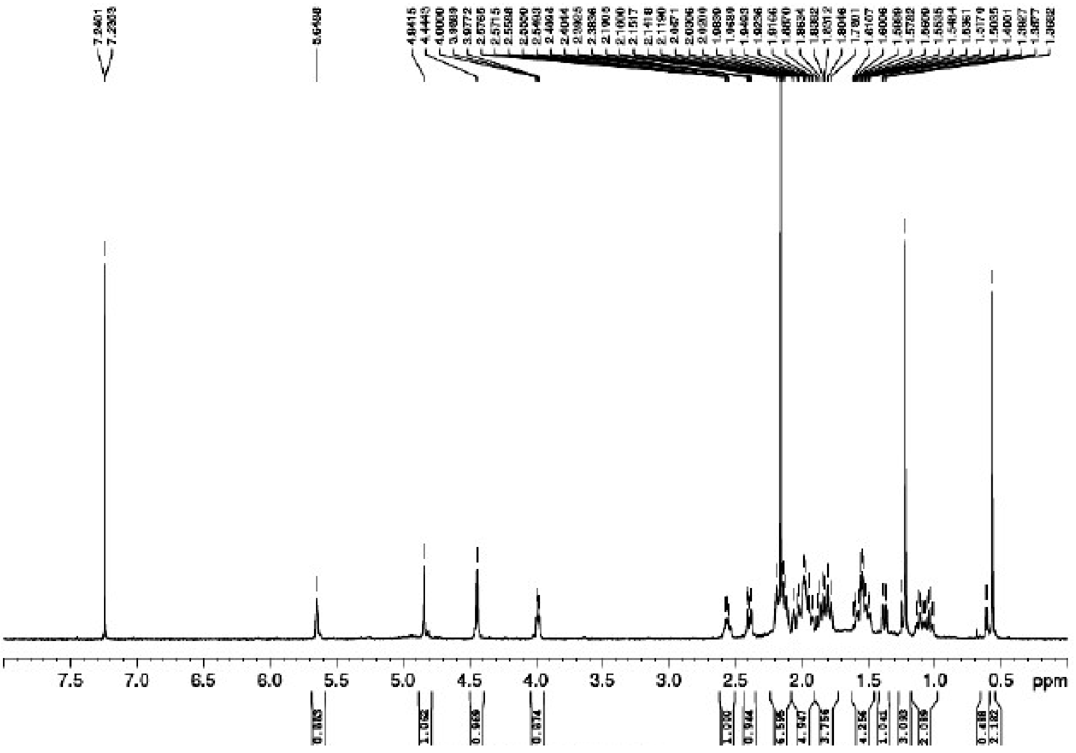

Figure S29. ^{1}H NMR spectrum of **5**

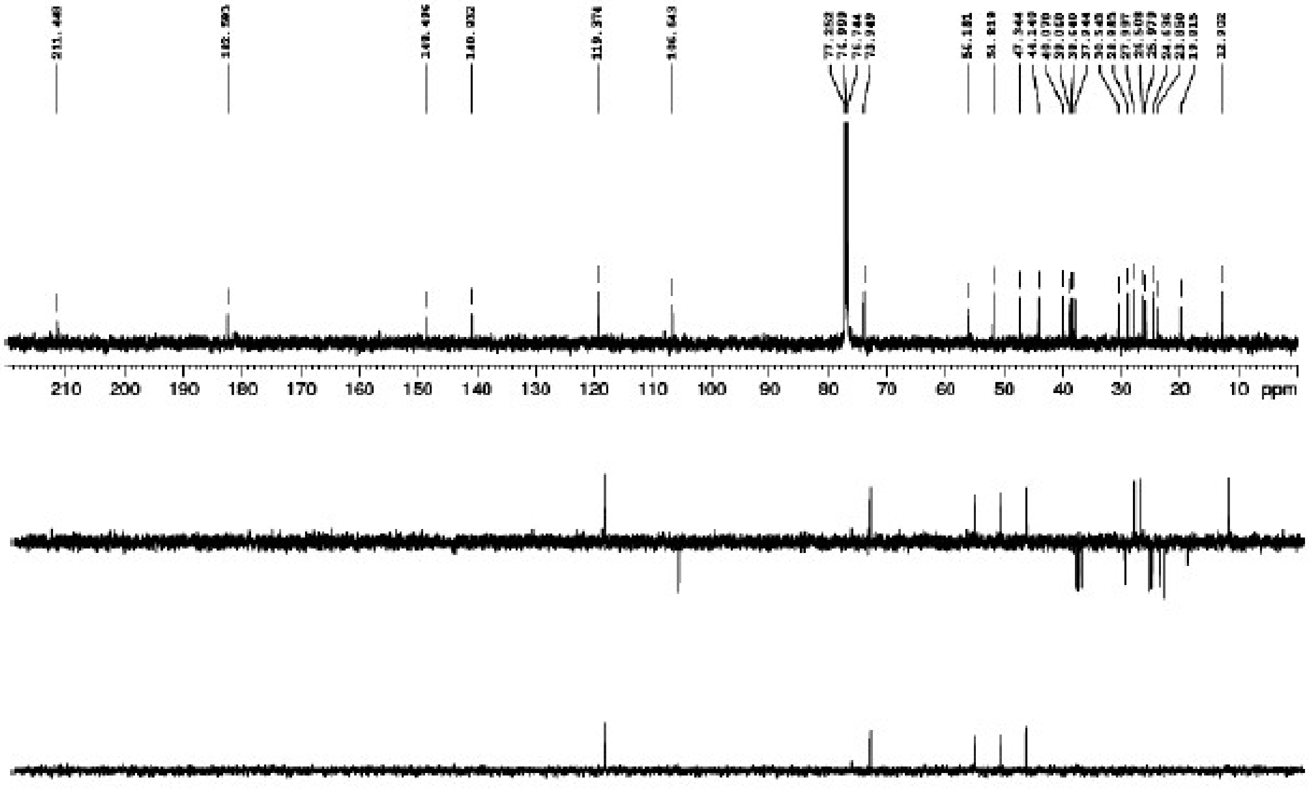

Figure 30. ^{13}C spectrum of **5**

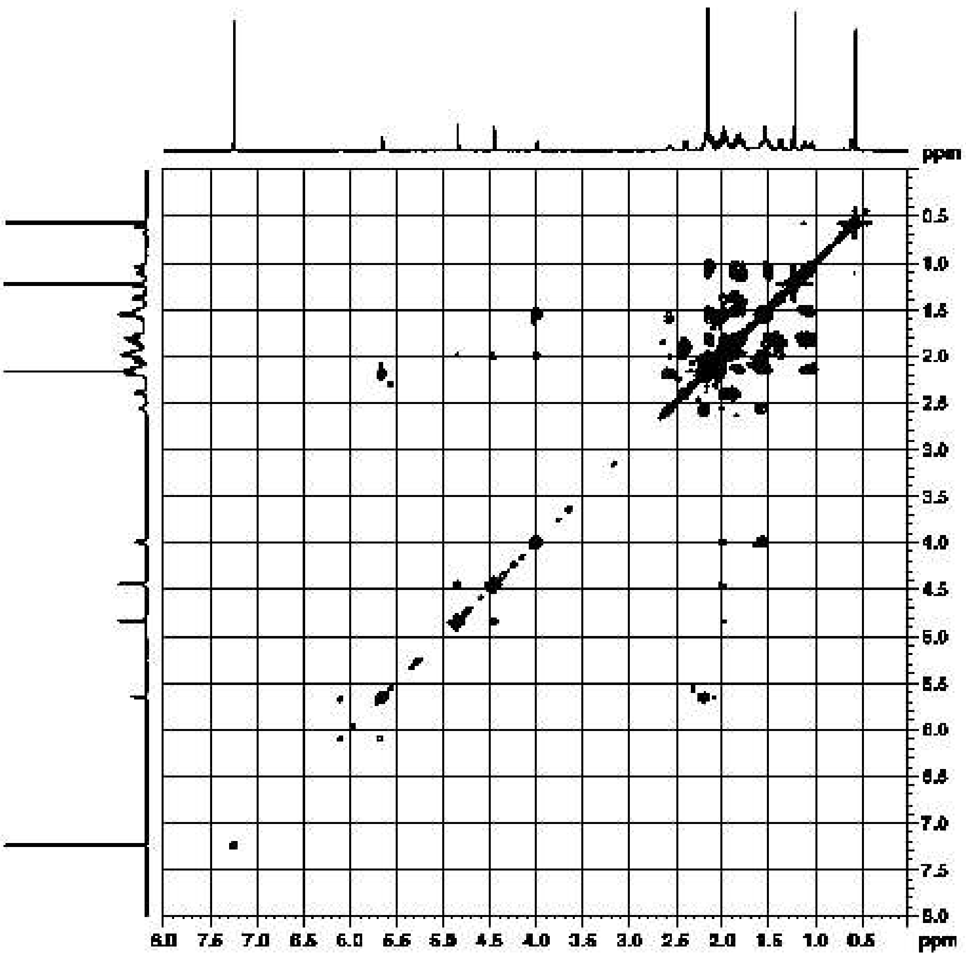

Figure S31. COSY spectrum of **5**

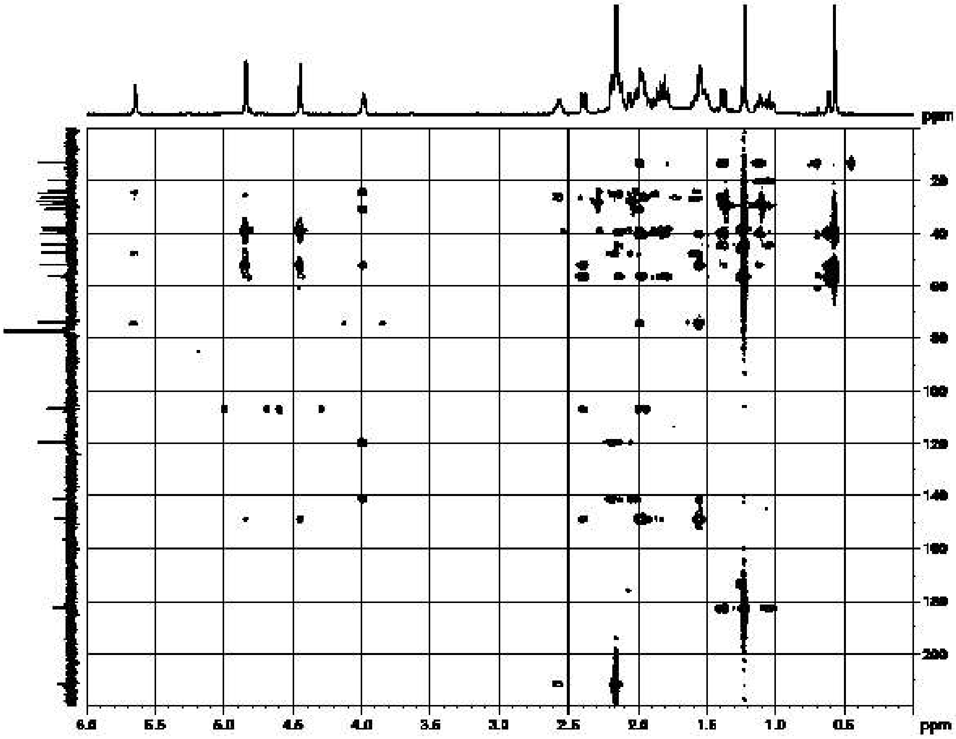

Figure S32. HMBC spectrum of **5**

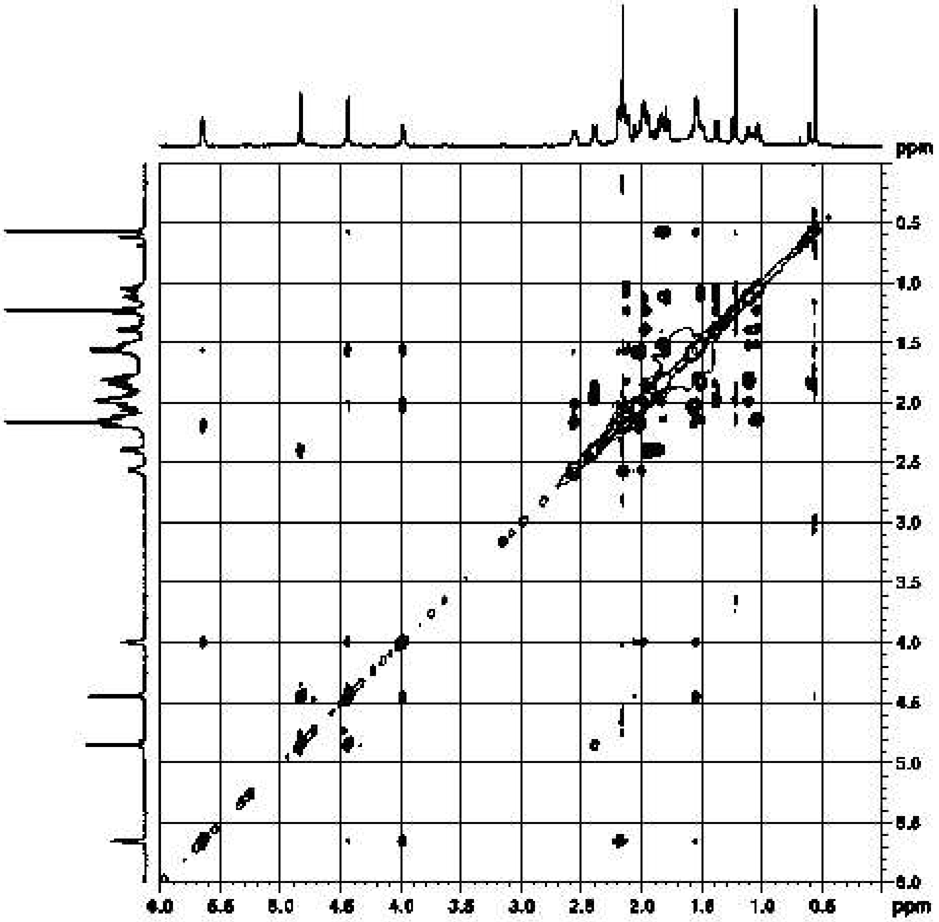

Figure S33. NOESY spectrum of **5**

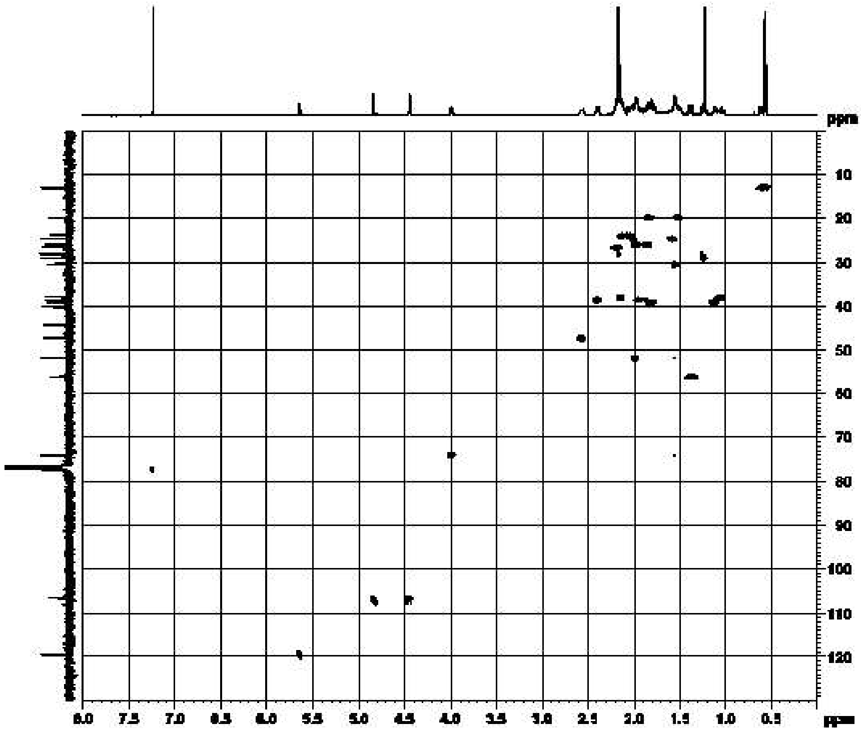

Figure S34. HSQC spectrum of **5**

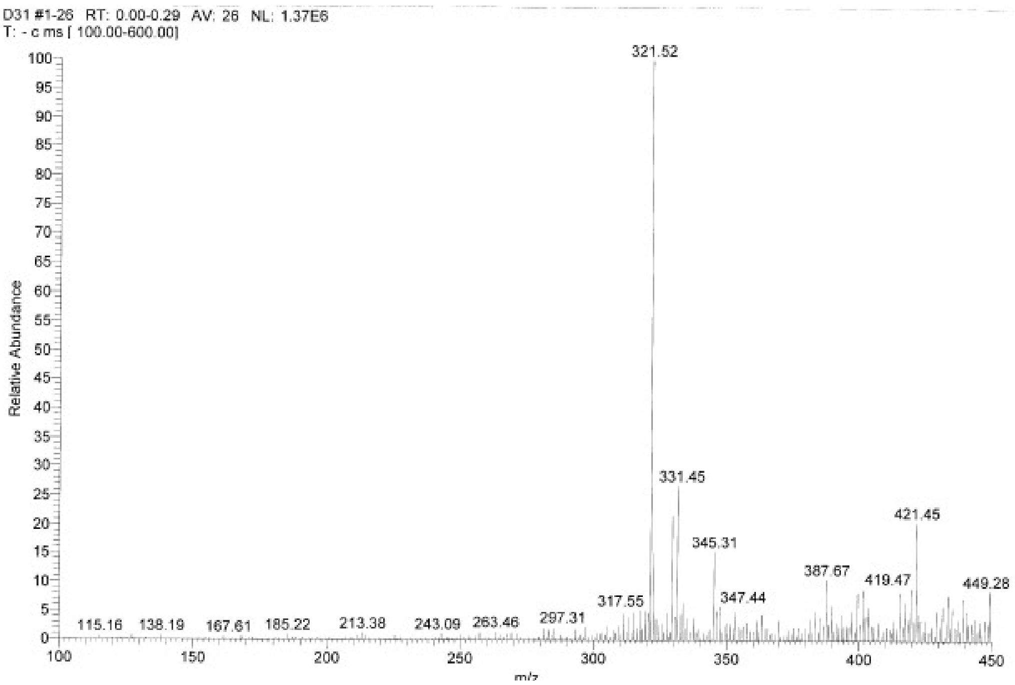

Figure S35. EI-MS spectrum of **5**

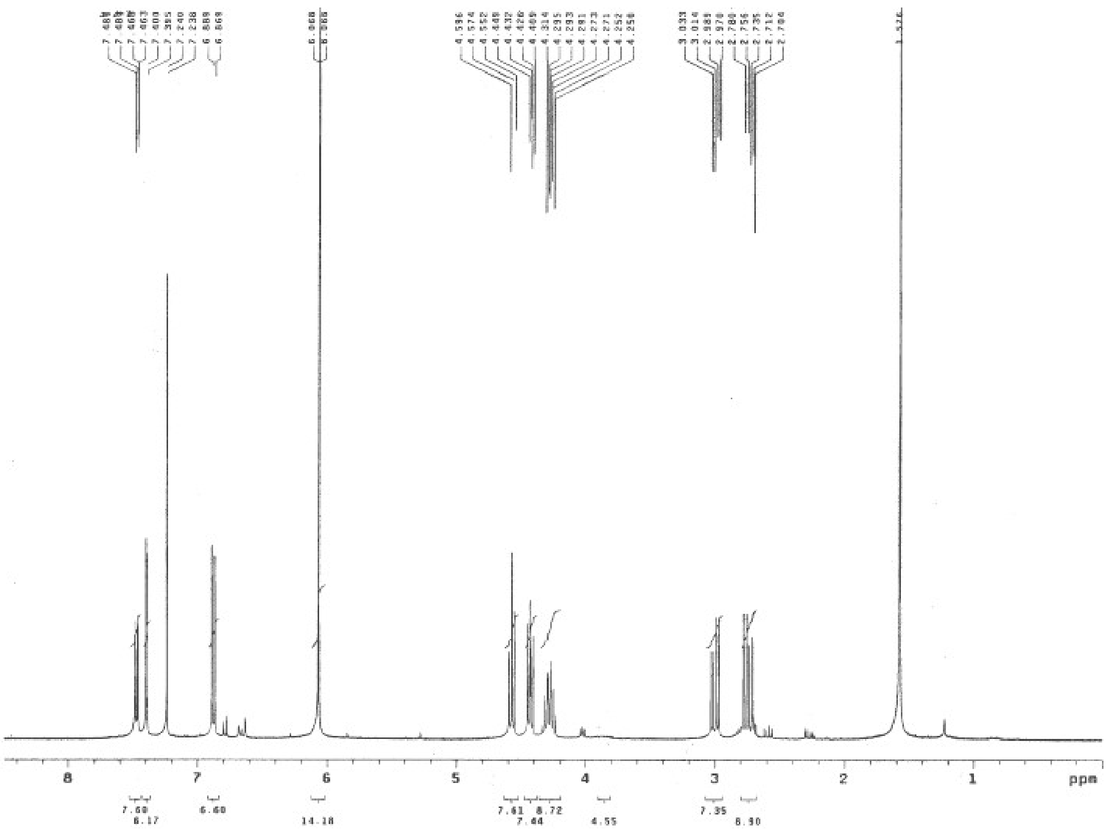

Figure S36. ^{1}H NMR spectrum of **6**

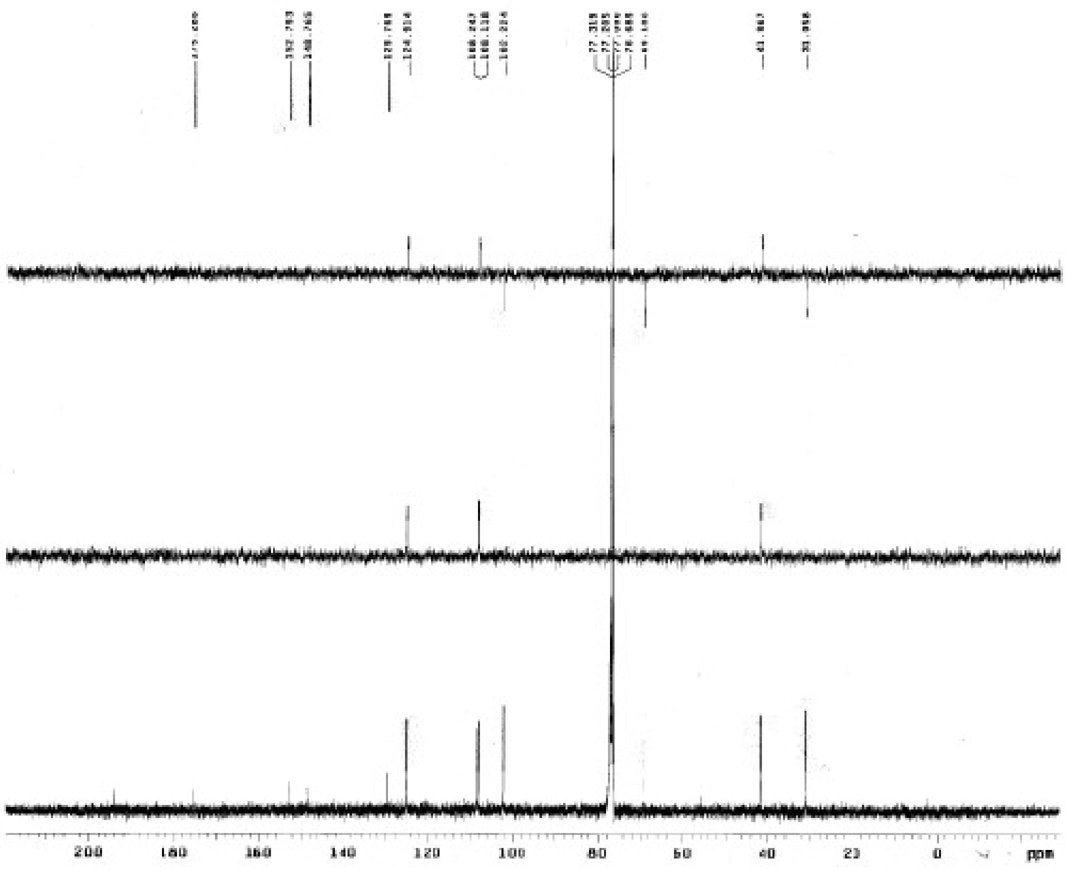

Figure S37. ^{13}C NMR/DEPT spectra of **6**

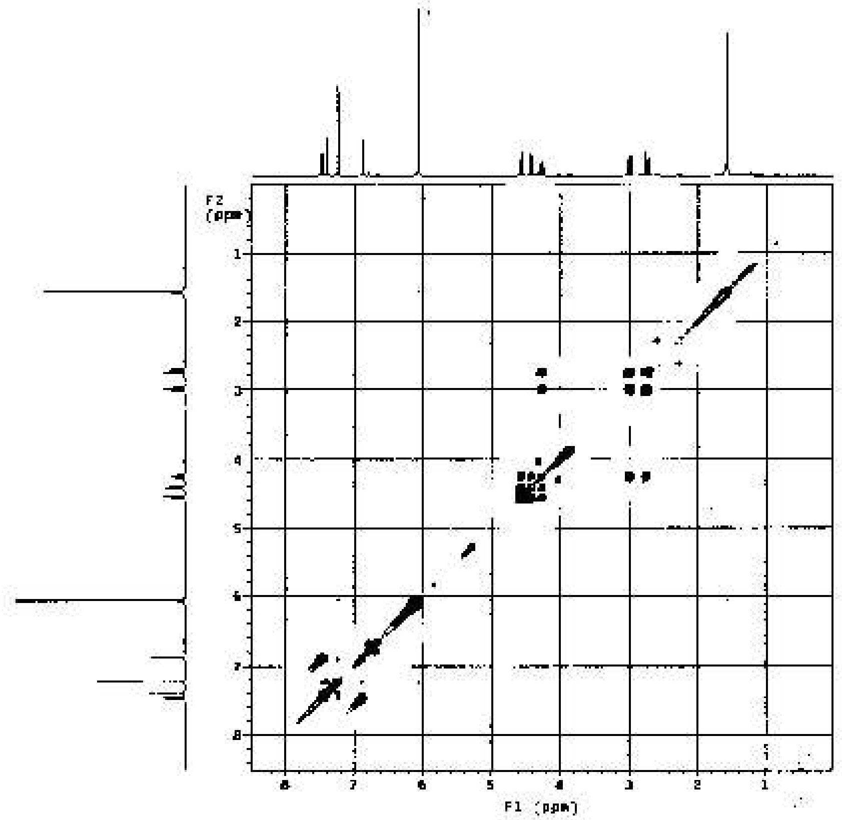

Figure S38. COSY spectrum of **6**

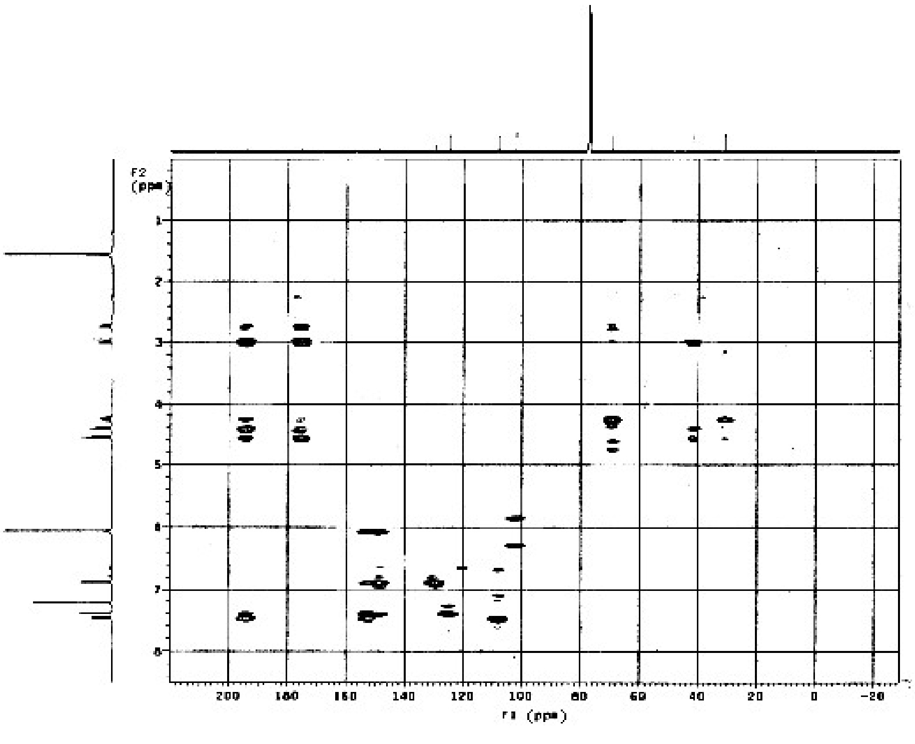

Figure S39. HMBC spectrum of **6**

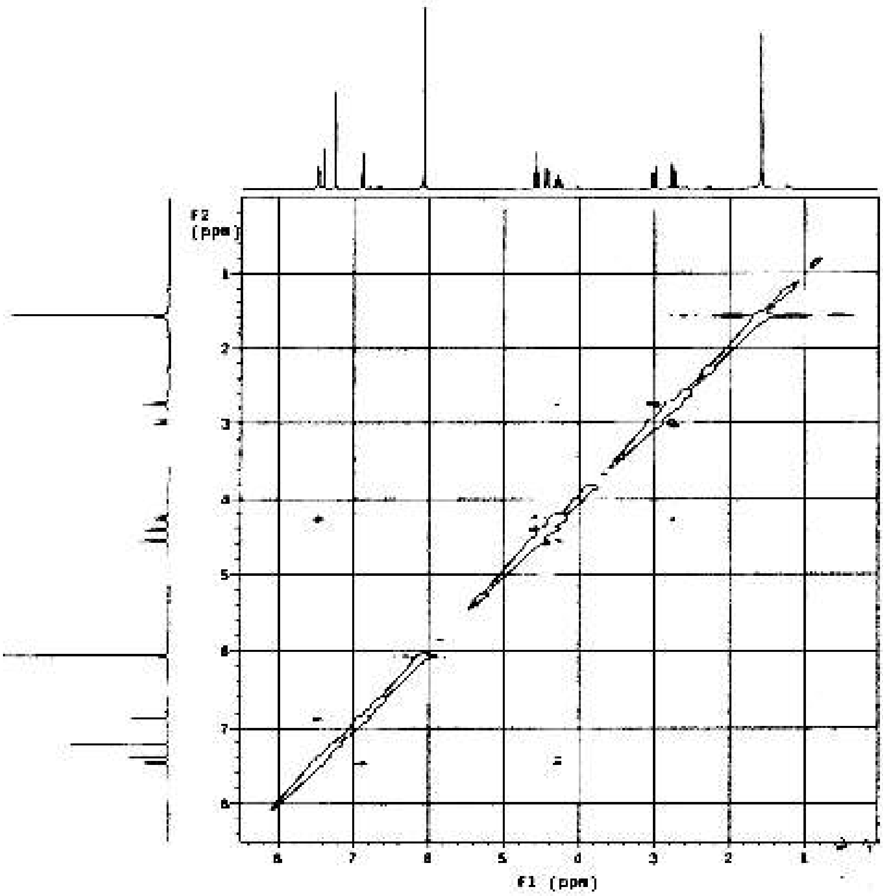

Figure S40. NOESY spectrum of **6**

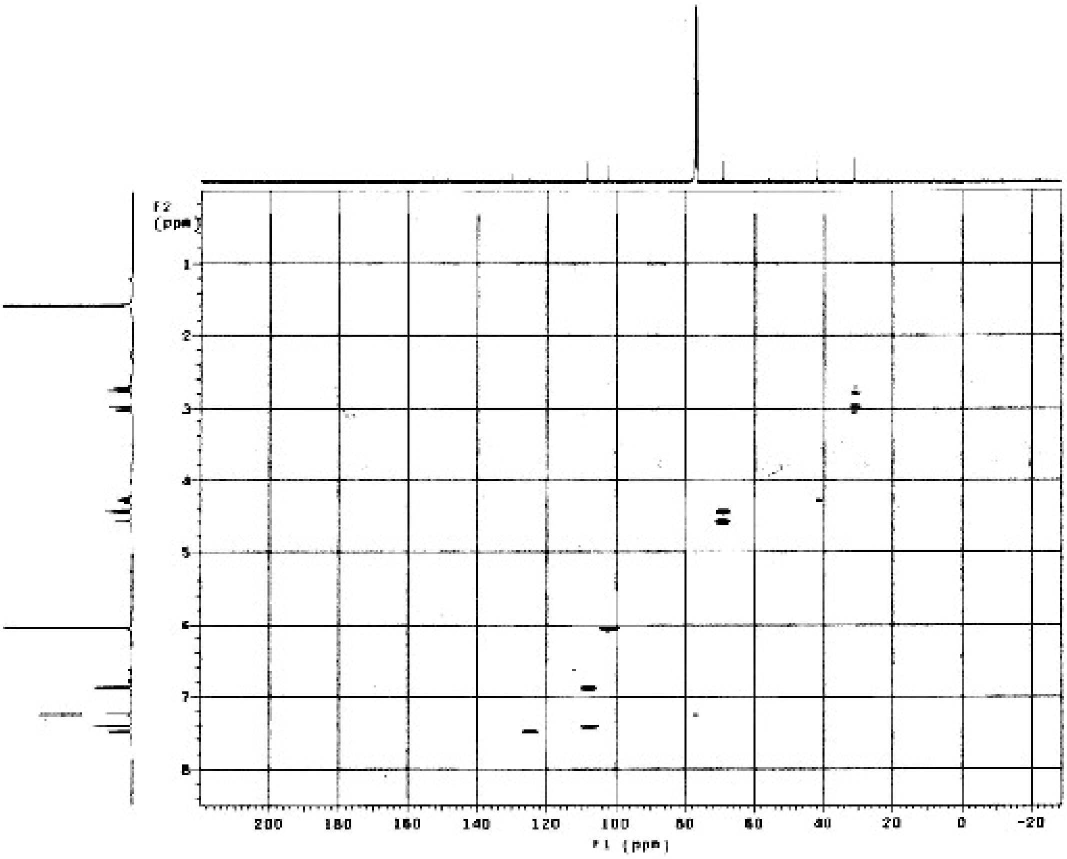

Figure S41. HSQC spectrum of **6**

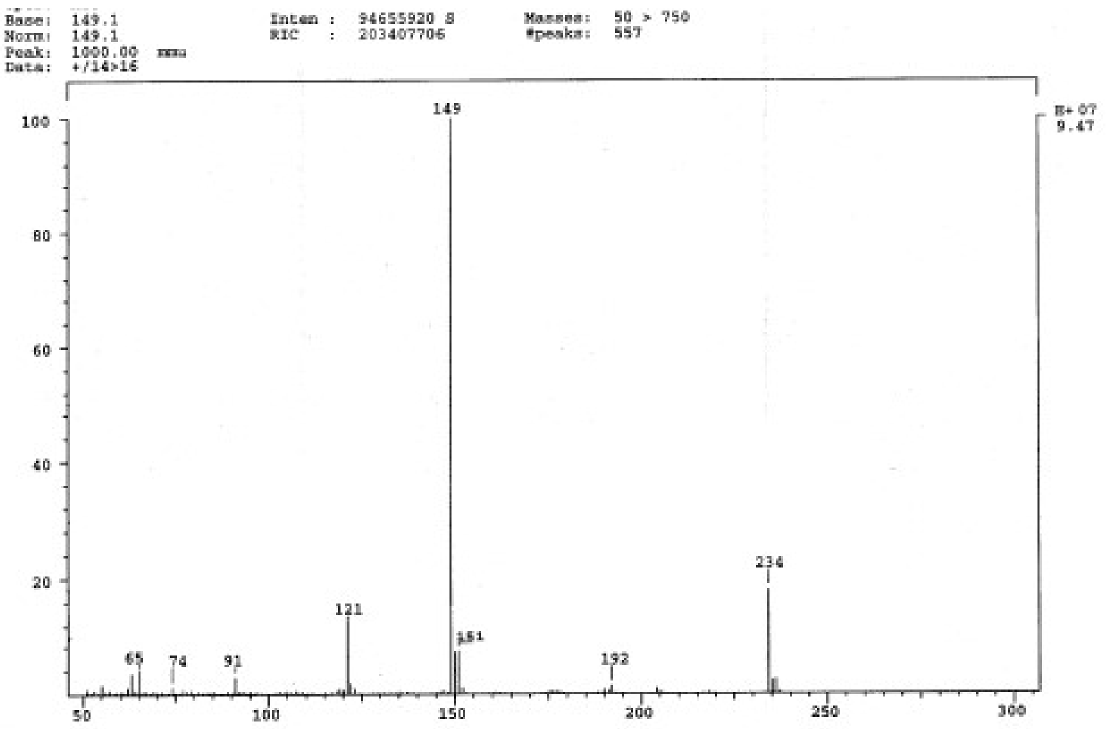

Figure S42. EI-MS spectrum of **6**

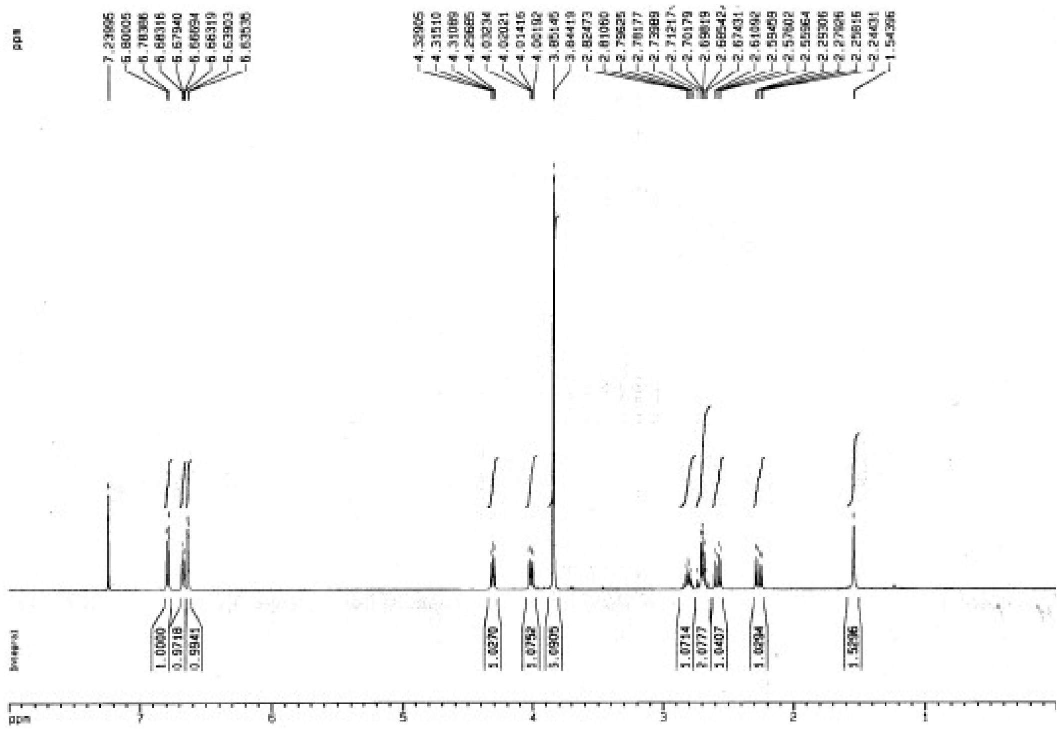

Figure S43. ^{1}H NMR spectrum of **7**

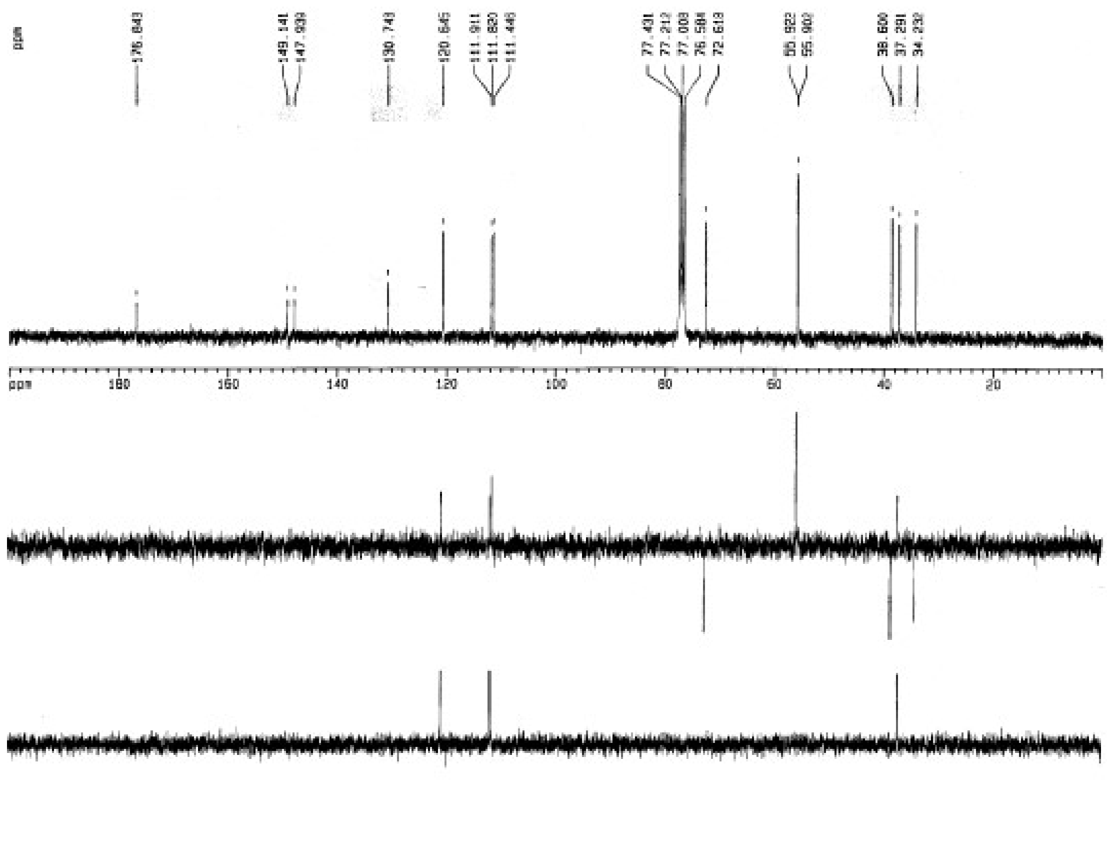

Figure S44. ^{13}C NMR/DEPT spectra of **7**

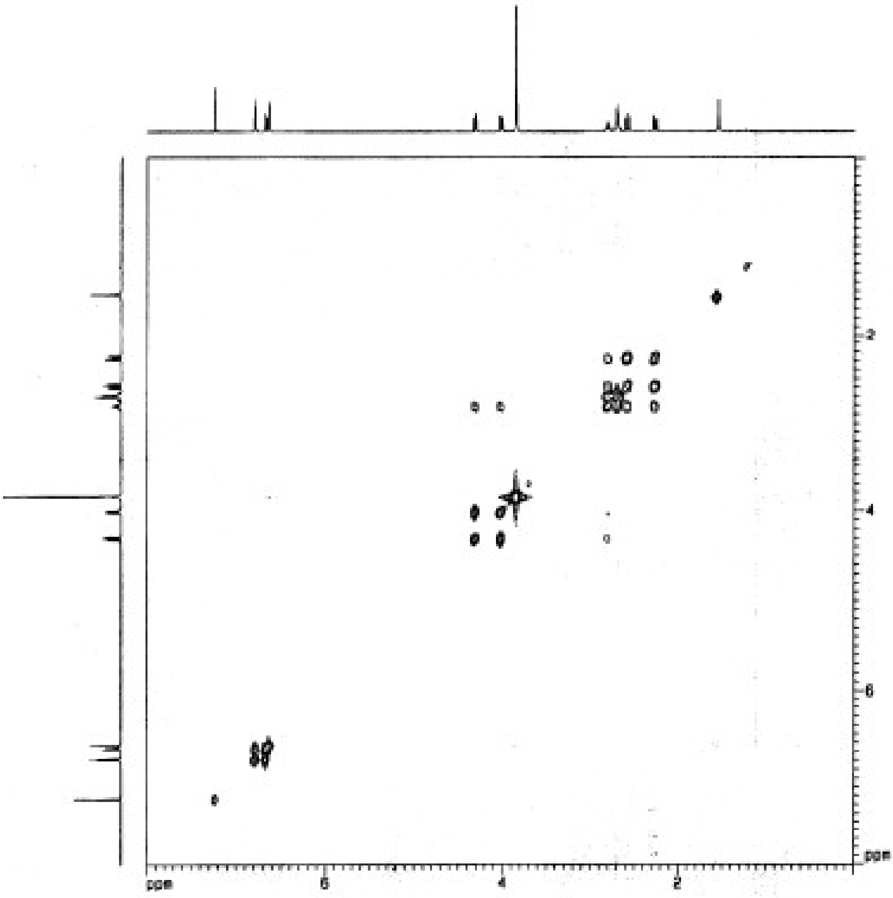

Figure S45. COSY spectrum of **7**

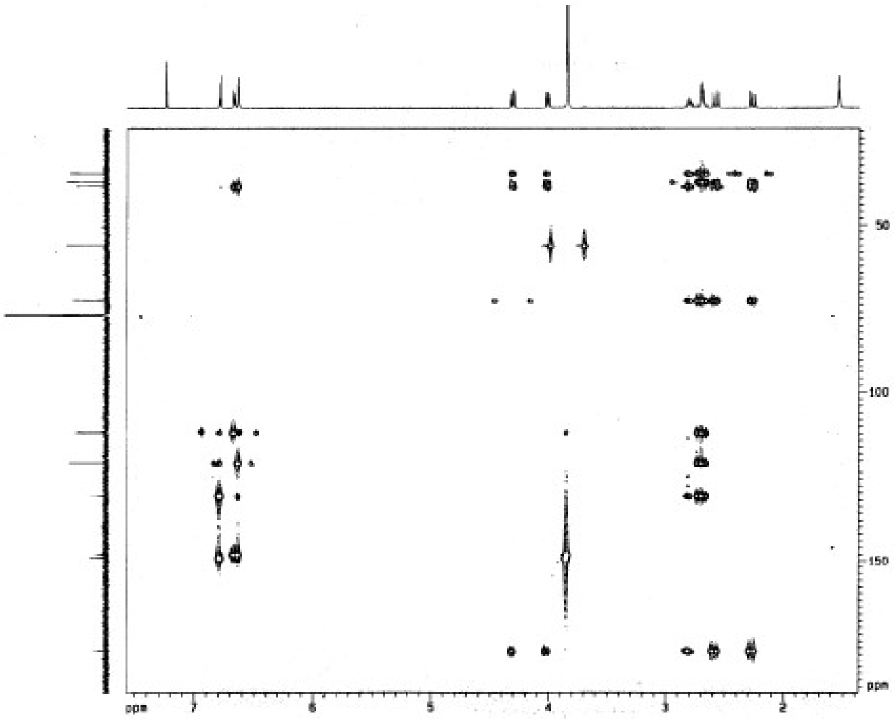

Figure S46. HMBC spectrum of **7**

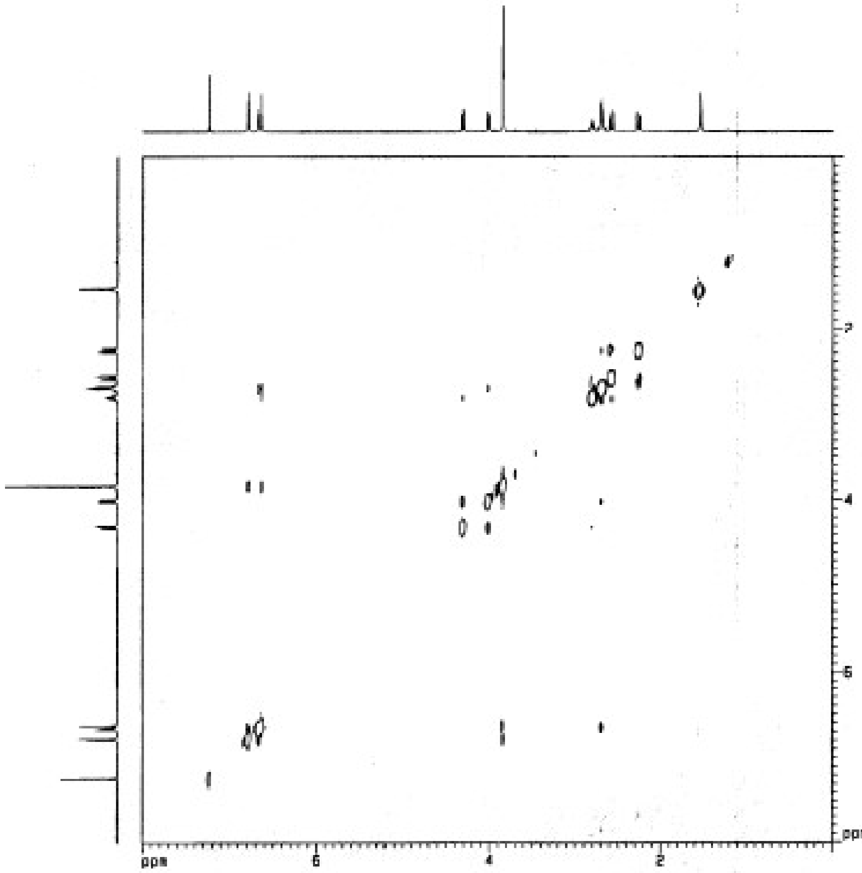

Figure S47. NOESY spectrum of **7**

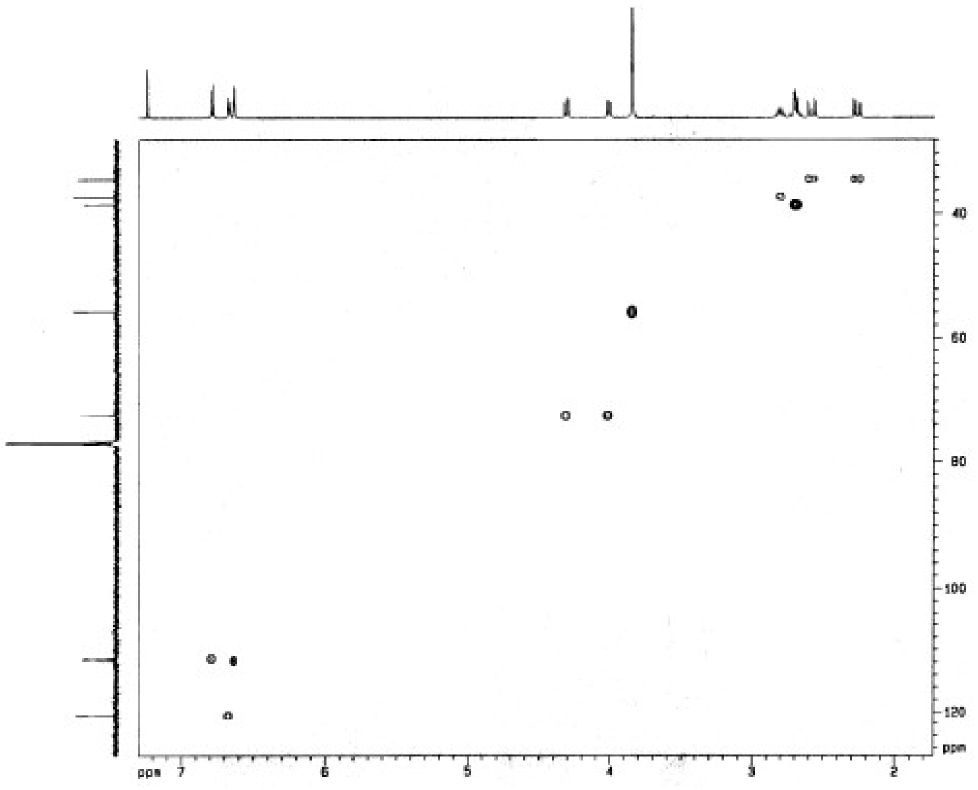

Figure S48. HSQC spectrum of **7**

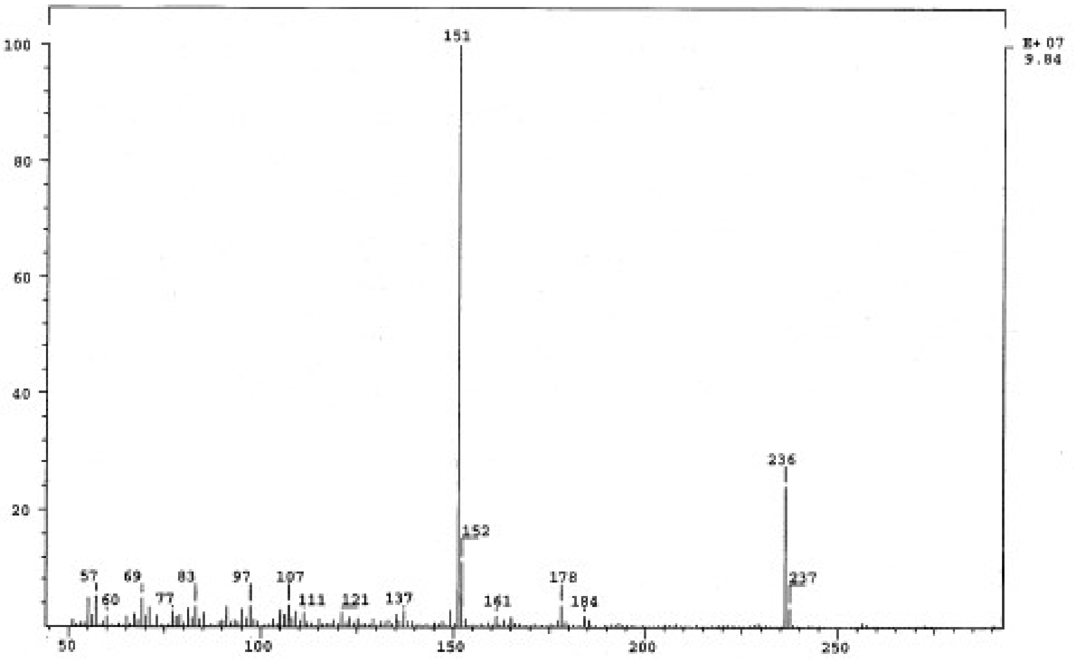

Figure S49. EI-MS spectrum of **7**

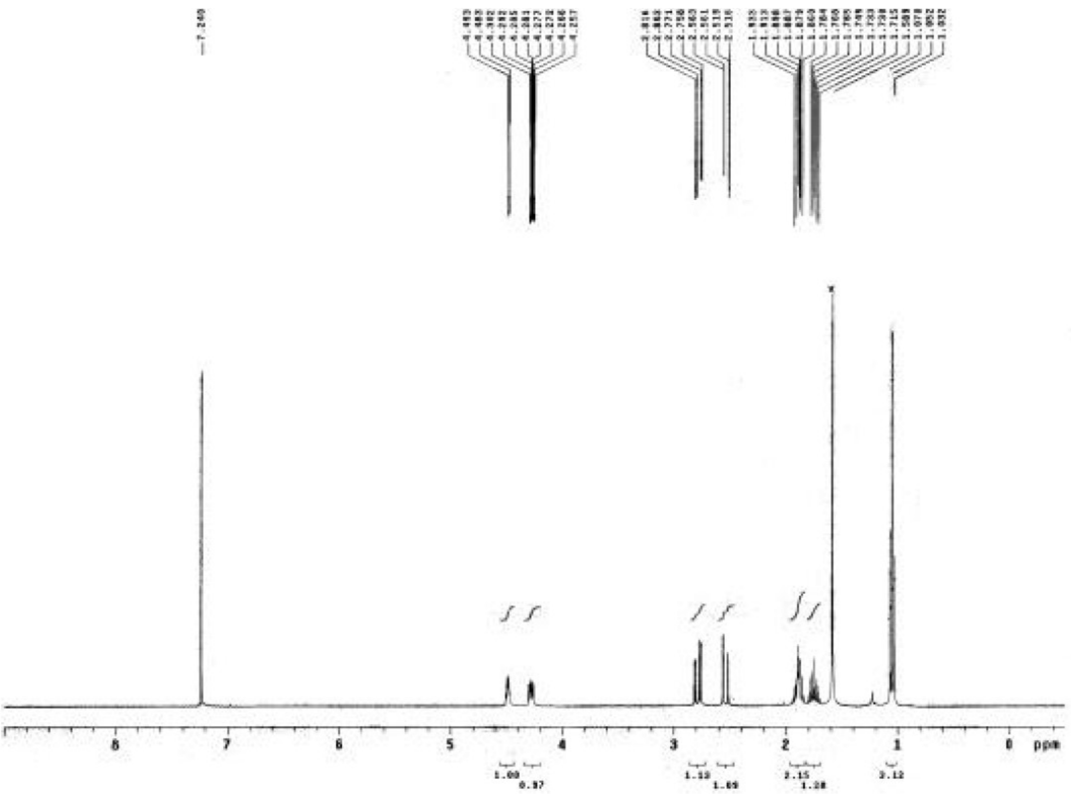

Figure S50. ^{1}H NMR spectrum of **8**

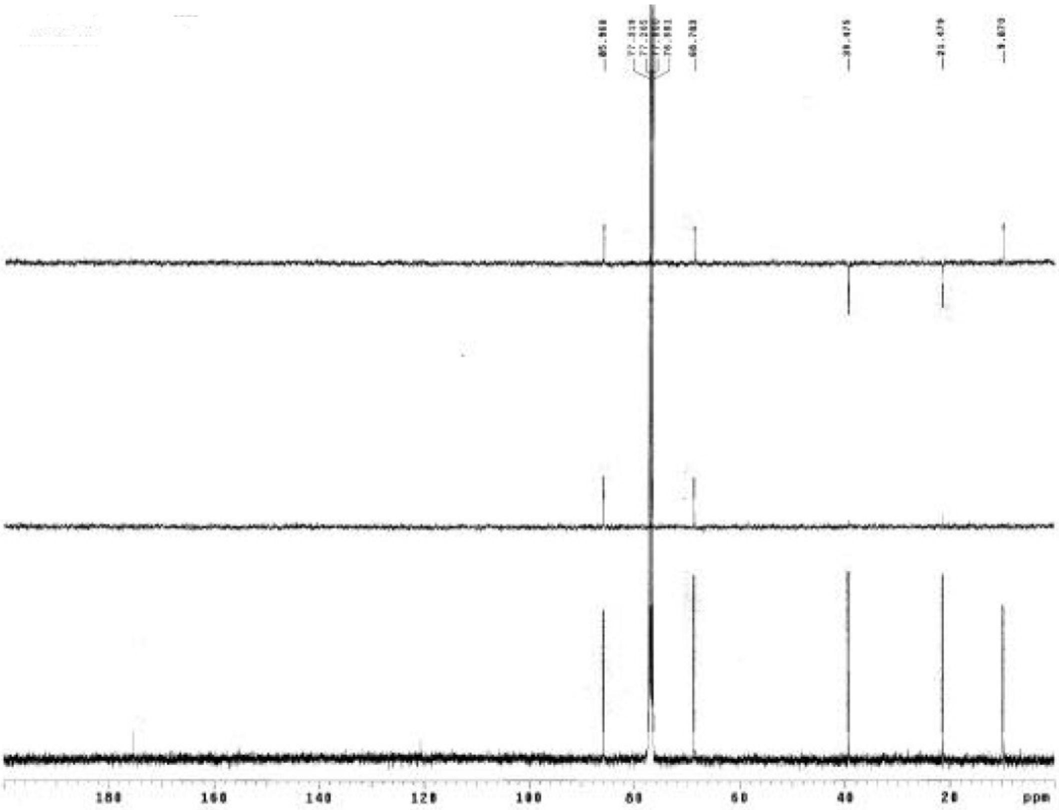

Figure S51. ^{13}C NMR/DEPT spectra of **8**

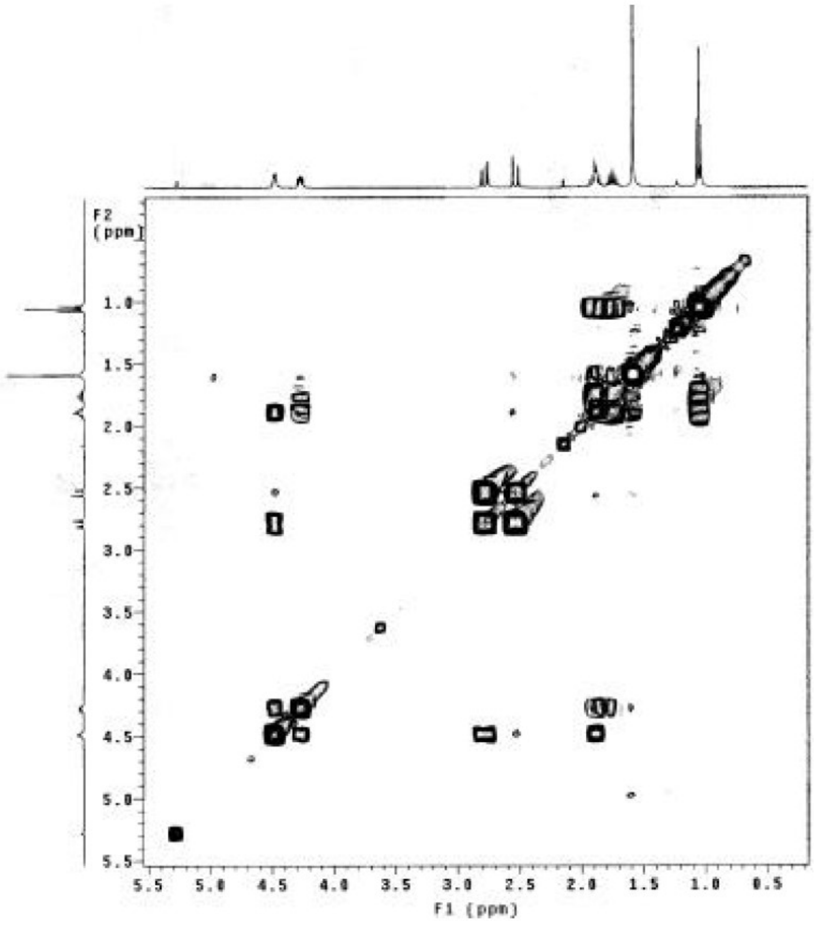

Figure S52. COSY spectrum of **8**

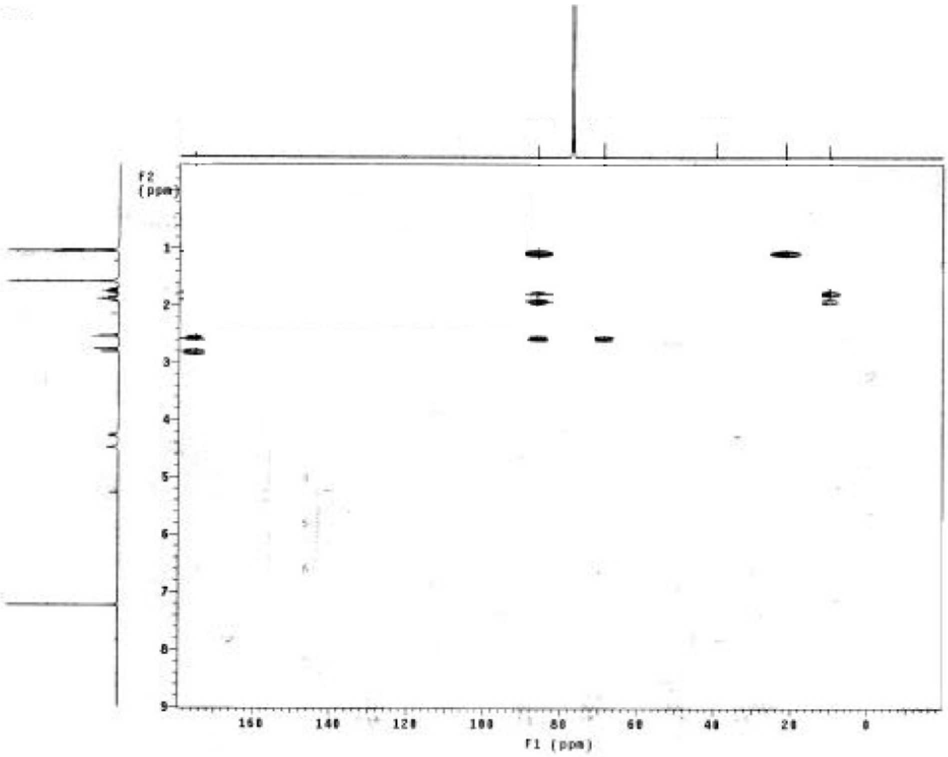

Figure S53. HMBC spectrum of **8**

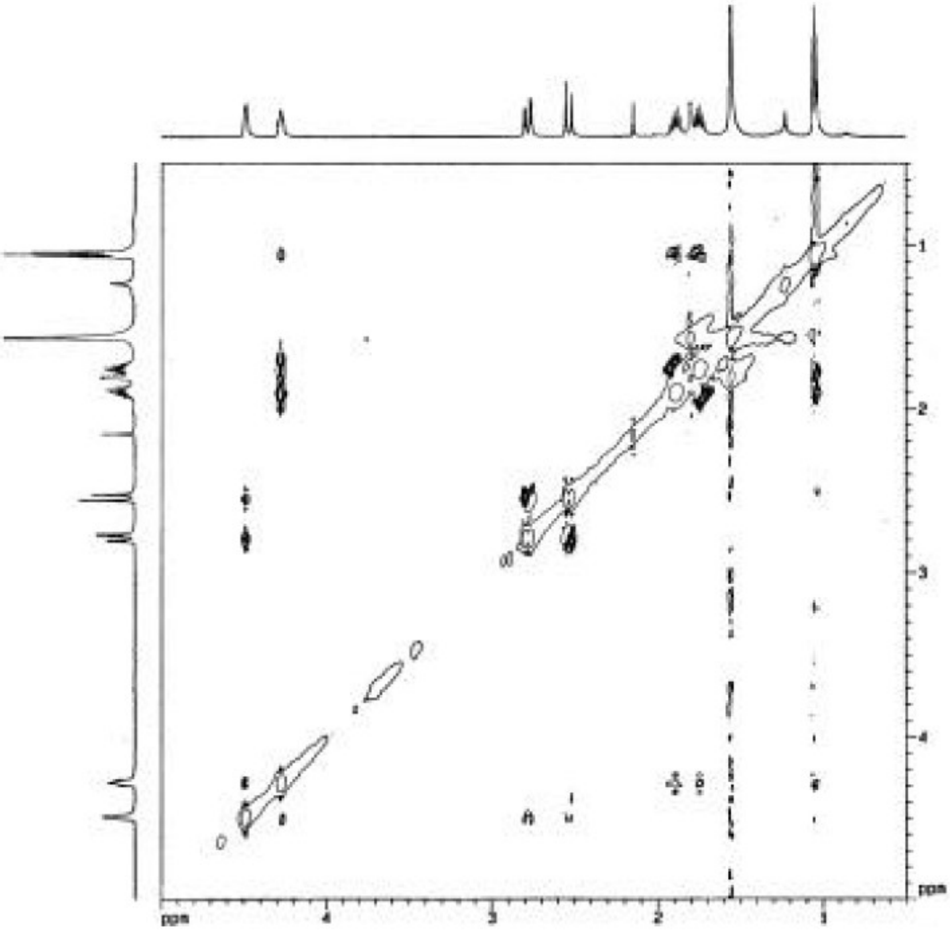

Figure S54. NOESY spectrum of **8**

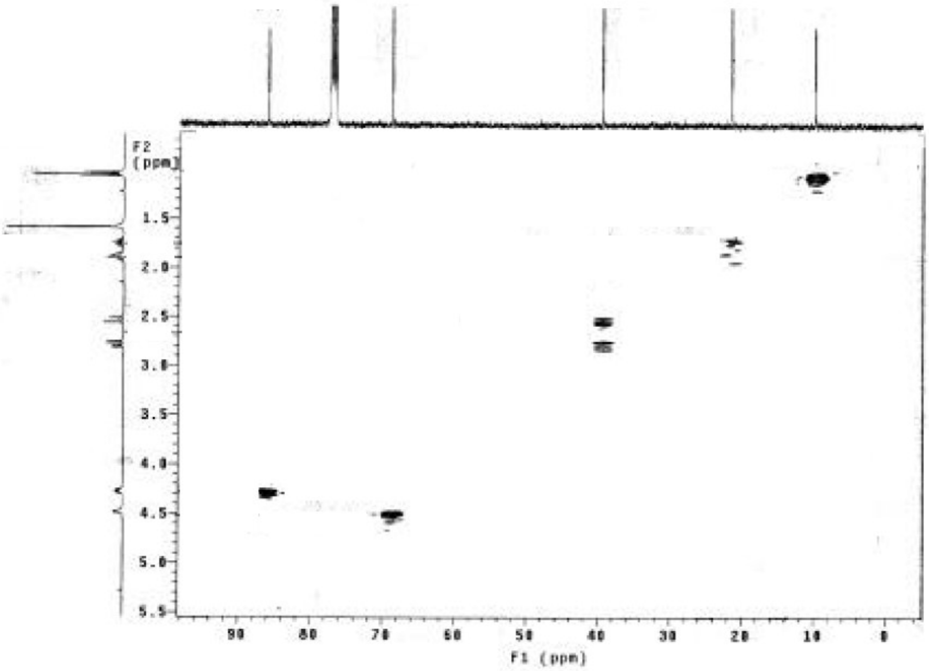

Figure S55. HSQC spectrum of **8**

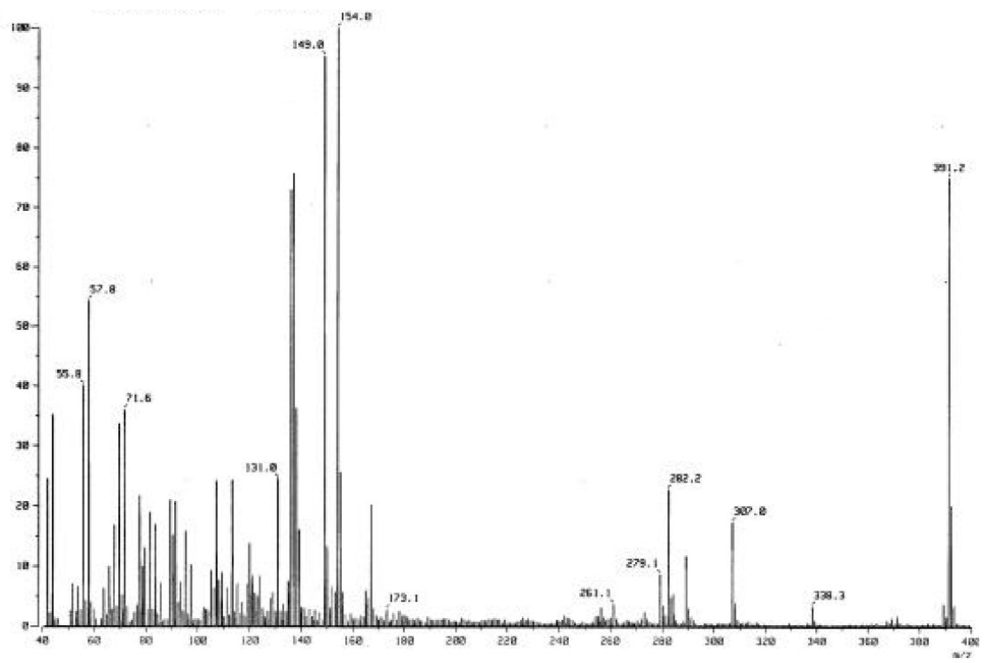

Figure S56. EI-MS spectrum of **8**

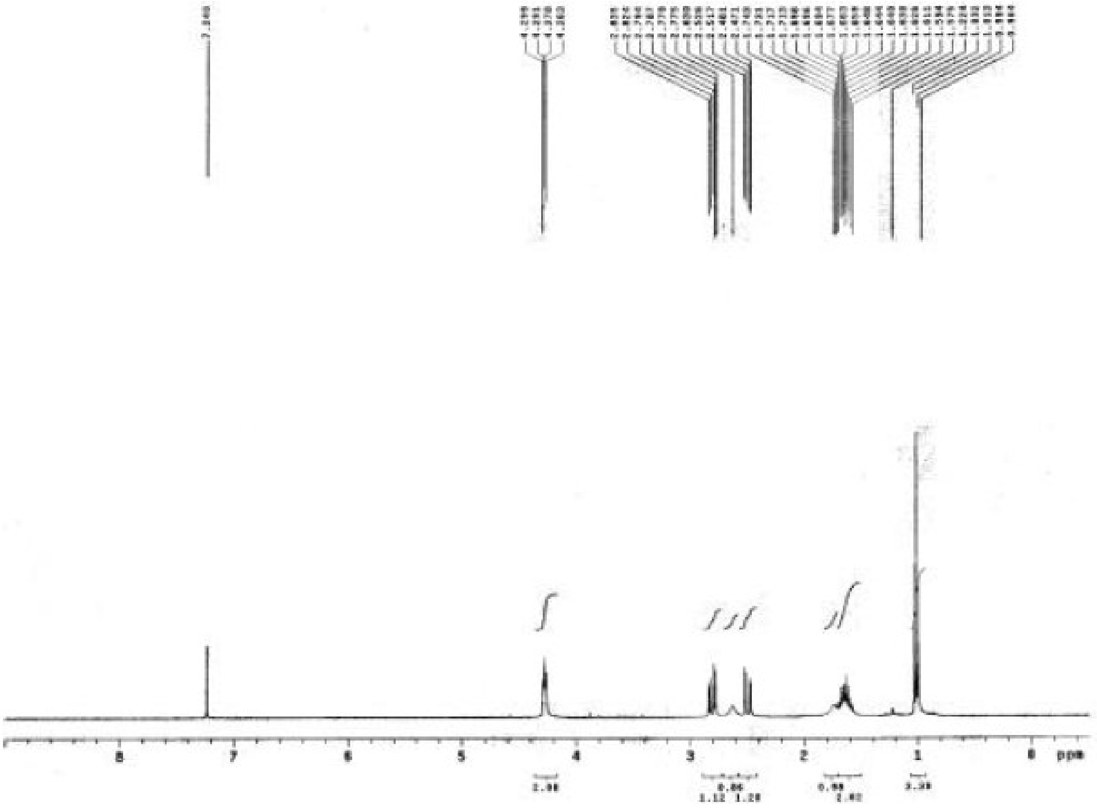

Figure S57. ^{1}H NMR spectrum of **9**

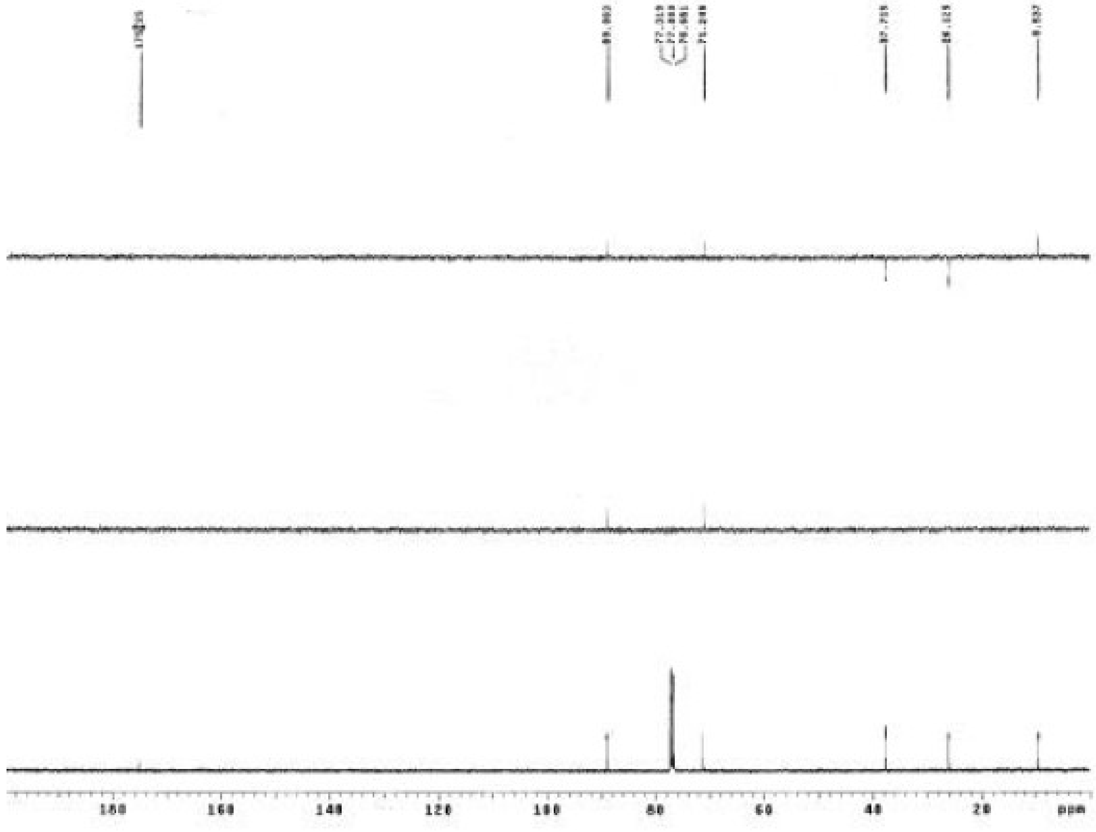

Figure S58. ^{13}C NMR/DEPT spectra of **9**

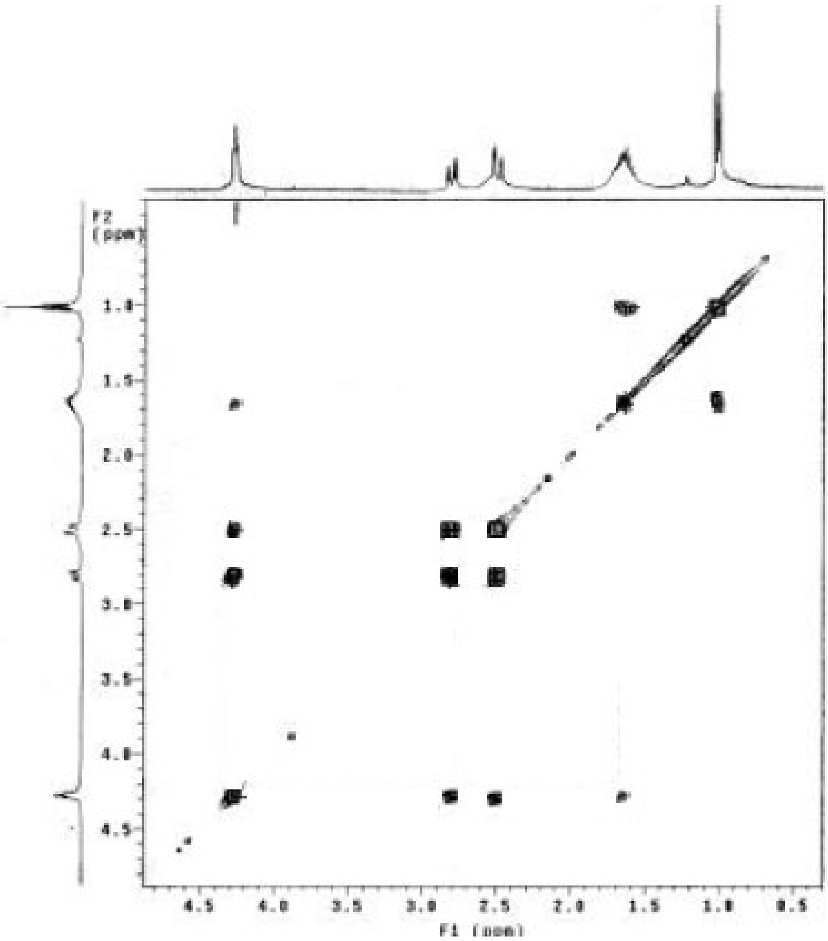

Figure S59. COSY spectrum of **9**

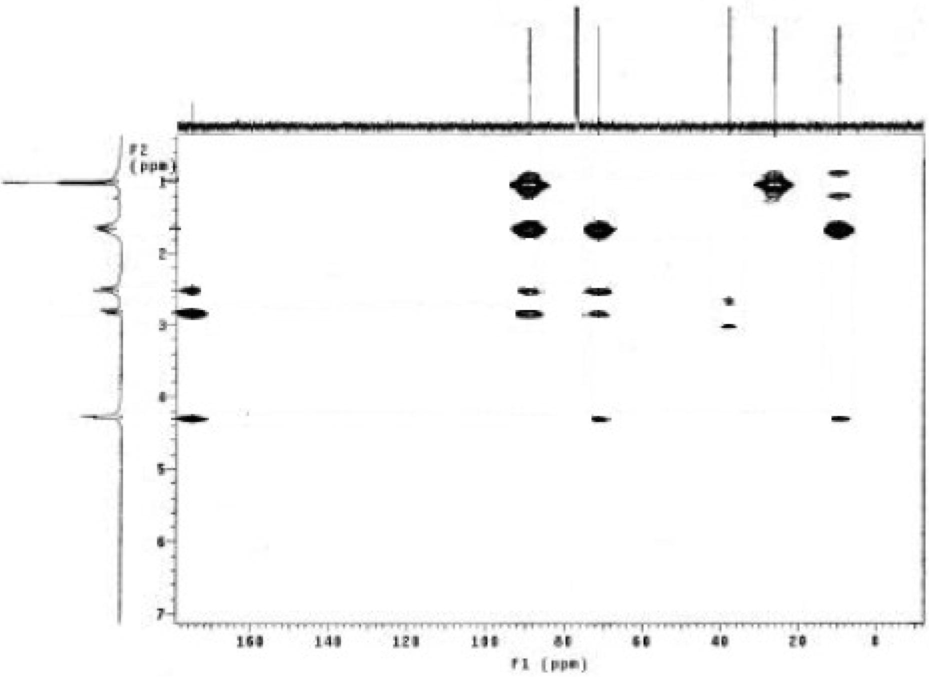

Figure S60. HMBC spectrum of **9**

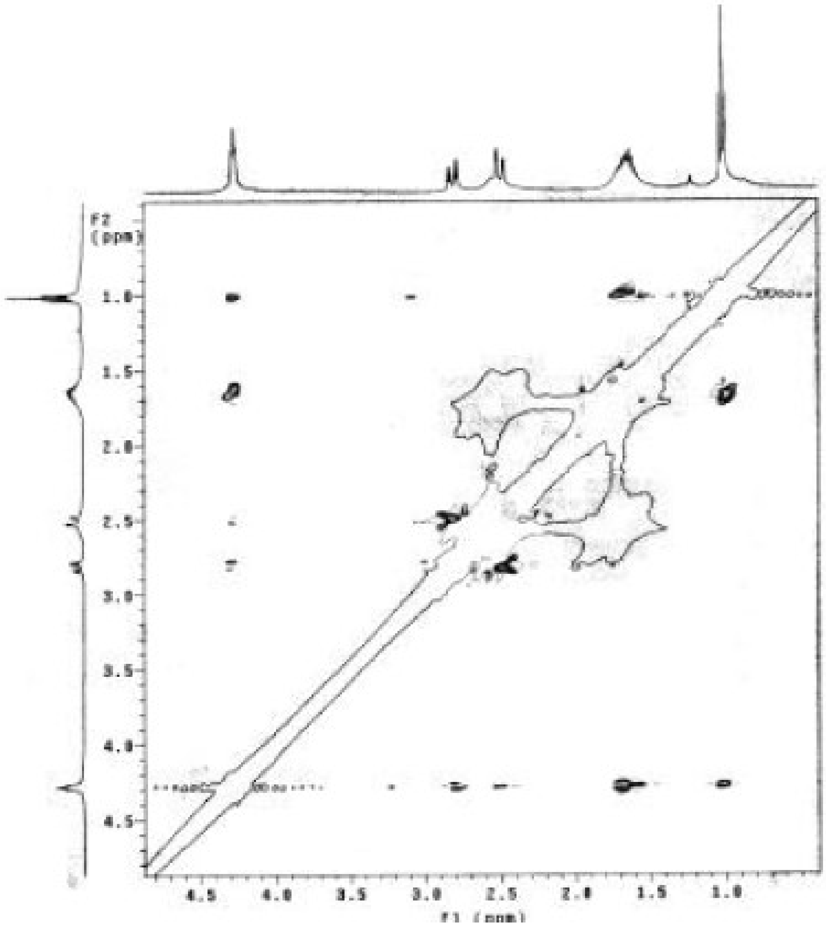

Figure S61. NOESY spectrum of **9**

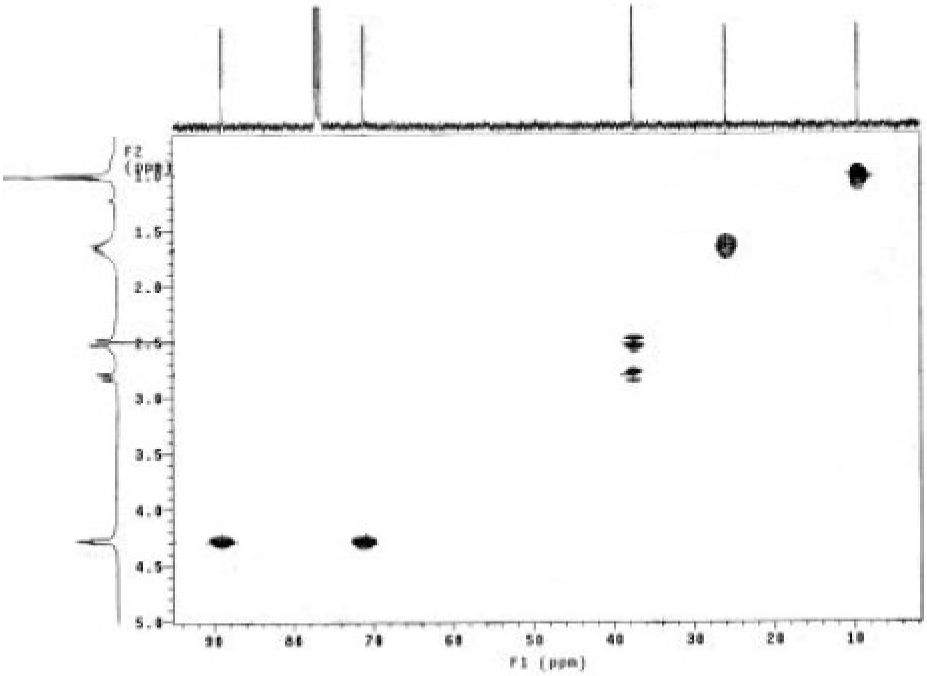

Figure S62. HSQC spectrum of **9**

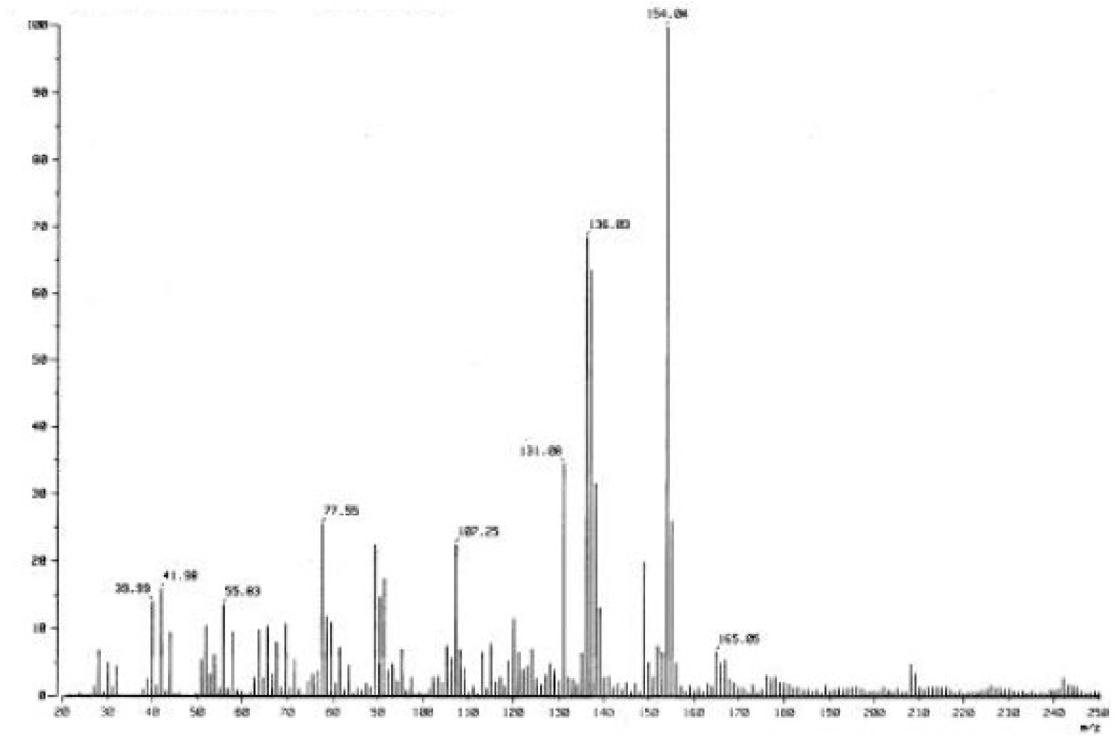

Figure S63. EI-MS spectrum of **9**
